# Green synthesis of (*R*)-3-hydroxy-decanoic acid and analogs from levoglucosenone: a novel access to the fatty acid moiety of rhamnolipids

**DOI:** 10.3389/fchem.2024.1362878

**Published:** 2024-04-19

**Authors:** Enzo Petracco, Amandine L. Flourat, Marie-Charlotte Belhomme, Stéphanie Castex, Fanny Brunissen, Fanny Brunois, Aurélien A. M. Peru, Florent Allais, Arnaud Haudrechy

**Affiliations:** ^1^ Institut de Chimie Moléculaire de Reims, UMR 7312, SFR Condorcet FR CNRS 3417, Université de Reims Champagne Ardenne, Reims, France; ^2^ URD Agro-Biotechnologies Industrielles (ABI), CEBB, AgroParisTech, Pomacle, France

**Keywords:** crop protection agent, cross-coupling, green chemistry, levoglucosenone, Michael addition

## Abstract

Rhamnolipids (RLs) are highly valuable molecules in the cosmetic, pharmaceutic, and agricultural sectors with outstanding biosurfactant properties. In agriculture, due to their potential to artificially stimulate the natural immune system of crops (also known as elicitation), they could represent a critical substitute to conventional pesticides. However, their current synthesis methods are complex and not aligned with green chemistry principles, posing a challenge for their industrial applications. In addition, their bioproduction is cumbersome with reproducibility issues and expensive downstream processing. This work offers a more straightforward and green access to RLs, crucial to decipher their mechanisms of action and design novel potent and eco-friendly elicitors. To achieve this, we propose an efficient seven-step synthetic pathway toward (*R*)-3-hydroxyfatty acid chains present in RLs, starting from cellulose-derived levoglucosenone, with Michael addition, Baeyer–Villiger oxidation, Bernet–Vasella reaction, and cross-metathesis homologation as key steps. This method allowed the production of (*R*)-3-hydroxyfatty acid chains and derivatives with an overall yield ranging from 24% to 36%.

## Introduction

**FIGURE 1:**
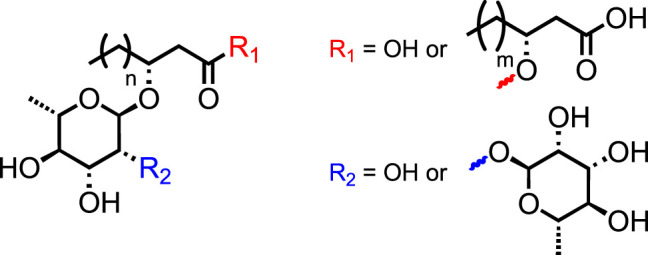
General structure of rhamnolipids.

Rhamnolipids (RLs) are glycolipidic biosurfactants composed of two key moieties: L-rhamnose unit(s) as hydrophilic head and a 3-(hydroxyalkanoyloxy)-alkanoic acid (HAA) fatty acid tail (Figure 1). The variations in this core structure allow the categorization of RLs into four main subgroups, called congeners: the mono-rhamno-mono-lipidic, the mono-rhamno-di-lipidic, the di-rhamno-mono-lipidic, and the di-rhamno-di-lipidic ones (Abdel-Mawgoud et al., 2010). Within these congeners, the RLs are described with regard to the length of the HAAs, providing a wide range of molecules with various highly valuable properties for many industrial applications. Indeed, the increasing interest in these molecules has made them serious competitors to synthetic surfactants in numerous industrial sectors, such as oil recovery (Rahman et al., 2003; Costa et al., 2010), therapeutics (Magalhães and Nitschke, 2013), cosmetics (Piljac and Piljac, 1999), cleaners (Parry et al., 2013), and agriculture (Sachdev and Cameotra, 2013; Miao et al., 2015; Dashtbozorg et al., 2016), with spectacular “eco-friendly” properties (*e.g.*, biosourced, biocompatibility, biodegradability, and biostimulant activity) (Randhawa and Rahman, 2014). In addition, RLs have shown tremendous potential for academic studies dealing with the activation of crop immunity, a novel alternative to pesticides (Schellenberger et al., 2021). They are commonly found in various bacterial species, which makes them recognizable by the plant as a pathogen signal, triggering an immune response *in planta*. This eliciting property, characteristic of microbe-associated molecular patterns (MAMPs) and pathogen-associated molecular patterns (PAMPs), has great potential for future crop management (Héloir et al., 2019). Nevertheless, the origin of this eliciting property is still unsettled and requires assays with analogous molecules to better unveil the chemical function responsible for these promising properties. Hence, the development of straightforward, high-yielding, and sustainable synthetic pathways toward RLs and analogs is not only a great tool for academic research purposes but also for the production of potential agrochemicals for pest control.

**SCHEME 1:**
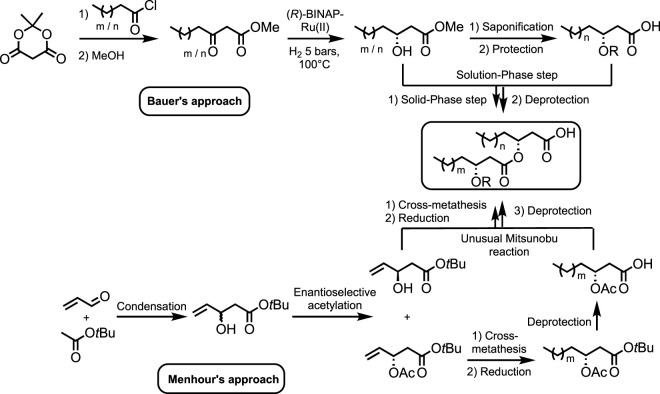
Synthetic strategies developed by Bauer et al. and Menhour et al. to synthesize HAAs (Bauer et al., 2006; Menhour et al., 2015).

The chemical synthesis of RLs is challenging, and synthetic routes reported in the literature lack diversity with only a few methods and starting materials (Bauer and Rademann, 2005; Bauer et al., 2006; Coss et al., 2012; De Vleeschouwer et al., 2014; Menhour et al., 2015; Menhour et al., 2016; Palos Pacheco et al., 2017; Compton et al., 2020; Cloutier et al., 2021). The method by Bauer et al., called hydrophobically assisted switching phase (HASP) synthesis, allows the production of a wide range of rhamnolipids but requires the independent total synthesis of each HAA, making the preparation of analogs cumbersome (Scheme 1). (Bauer and Rademann, 2005; Bauer et al., 2006) In addition, although elegant, this methodology suffers from important quantities of toxic solvents (*e.g.*, methanol and chloroform) and atom-consuming protection/deprotection sequences, although it remains the most widely used methodology (Coss et al., 2012; De Vleeschouwer et al., 2014; Palos Pacheco et al., 2017; Cloutier et al., 2021). The synthesis of (*R*)-3-hydroxy-alkanoic acids was also reported by Jaipuri et al. (2004). Although the overall yield was quite good (38%, six steps), synthetic steps required the use of toxic reagents and solvents, as well as a restrictive setup (*i.e.*, cooling at 0 or −78°C and microwave activation). In 2020, Compton et al. (2020) synthesized racemic 3-hydroxy decanoic acid using Reformatsky condensation.

We have previously published articles focusing on (di-)lipidic HAAs synthesis starting from an acetoxyester (Scheme 1) (Menhour et al., 2015; Menhour et al., 2016). This compound is generated in two steps: 1) the condensation of acrolein with *tert*-butyl acetate leading to the racemic *tert*-butyl 3-hydroxypent-4-enoate and 2) the subsequent PS Amano lipase-biocatalyzed enantioselective acetylation (Dulęba et al., 2020). Using this valuable starting material and cross-metathesis, a library of HAAs has been synthesized from this unique precursor. Although an unusual Mitsunobu reaction has allowed the creation of di-lipidic structures, this synthesis requires further development to comply with the green chemistry principles. For instance, it would be more attractive if a biosourced starting material was used.

**FIGURE 2:**
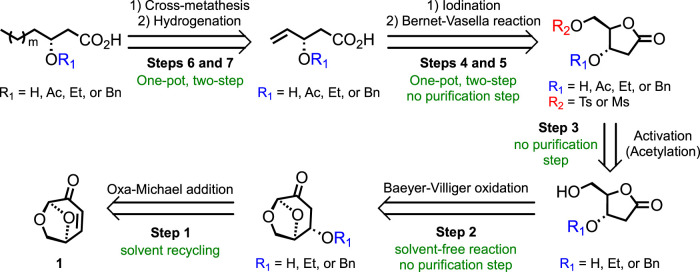
Retrosynthetic strategy designed and optimized in this work.

To address the aforementioned considerations, the work reported herein aims to develop a novel green synthetic pathway toward the HAA of RLs and analogs starting from levoglucosenone (**LGO 1**, Figure 2), a chiral biosourced synthon that can be obtained from cellulosic wastes through a catalytic aerobic fast pyrolysis approach (CFP), such as the Furacell™ process developed by Circa Group^®^ (Court et al., 2012). **LGO** is recognized as a highly valuable chemical due to 1) the preservation of some natural chiral centers of cellulose and 2) the α,β-unsaturated ketone and masked aldehyde functionalities that open the way to a wide range of synthetic strategies. The proposed seven-step retrosynthetic pathway is described in Figure 2. The flexibility of this methodology will efficiently provide a library of analogs that could be involved in the HASP process and, thus, the possibility of establishing structure/activity relationships (SARs).

Advantageously, the natural chirality of **LGO** (chiral pool) allows obtaining 3-hydroxy-fatty acids with the desired (*R*)-configuration without the use of enantioselective reactions (Figure 2). Carefully chosen strategies will be explored to achieve good yields and limit protection/deprotection steps.

## Results and discussion

### Step 1: oxa-Michael addition

**SCHEME 2:**
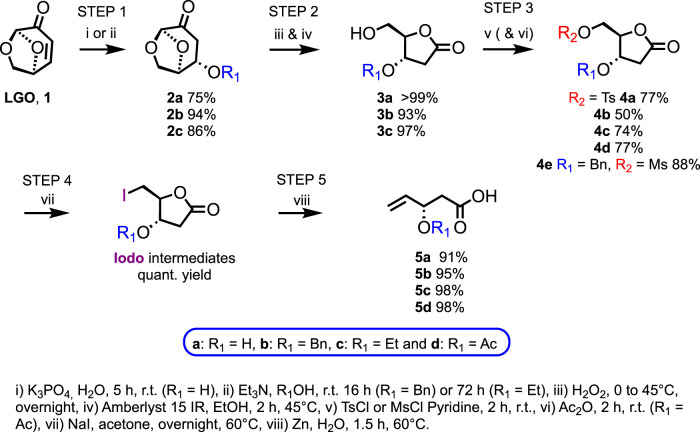
Synthesis of the **5** series from **LGO**.

As previously reported by Diot et al., the oxa-Michael addition of water onto **LGO** occurred in *exo* with total selectivity to form hydrated levoglucosenone **2a** in 75% yield (estimated with ^1^H NMR experiments) (Diot-néant et al., 2021). Approximately 10% of **LGO** remains in the crude mixture, along with 10% of a side product coming from **LGO** cross-coupling. Compound **2a** can be purified by flash chromatography over silica gel, leading to a 62% isolated yield. Alternatively, the two by-products can also be partially removed through extraction. Indeed, these undesired components are more soluble in an organic solvent, herein ethyl acetate, than in water. In contrast, **2a,** being more hydrophilic, remains mainly in the aqueous layer. Through this extraction, **2a** was recovered in a 75% yield, and its purity was increased from 75% to 94% (determined by ^1^H NMR), which was high enough to avoid the purification step (Scheme 2).

For benzyl series **b**, the oxa-Michael addition of benzyl alcohol onto **LGO** following the conditions described by Kawai et al. gave **2b** in a 94% yield (Kawai et al., 1995). As this step requires a large excess of benzyl alcohol (38 equiv.), the recyclability of benzyl alcohol was investigated to limit the ecological footprint. Interestingly, more than 98% of unreacted benzyl alcohol could be recovered through a short-path distillation (also known as molecular distillation).

The addition of ethanol to **LGO** was reported by Sharipov et al. (2019). However, we did not succeed in reproducing the 70% yield announced by Sharipov and co-workers using their conditions (0.5 equiv. Et_3_N, [**LGO**] = 77 mmol.L^−1^ in ethanol, 36 h, r.t.). To increase the yield further, a Design of Experiments (DoE) was performed on two factors: 1) concentration ranging from 0.08 to 0.5 mol.L^−1^, and 2) equivalence of Et_3_N varying between 0.5 and 2, over 72 h (Supplementary Section S3). To achieve the best HPLC yield (86%), the most diluted conditions (0.08 mol.L^−1^) with the highest amount of Et_3_N (2 equiv.) were required. Purification over silica gel was performed to fully recover **2c** in high yield (86%) and purity (Scheme 2).

### Step 2: Baeyer–Villiger oxidation

An organic solvent-free H_2_O_2_-mediated Baeyer–Villiger oxidation followed by an Amberlyst-catalyzed ethanolysis was performed on the **2** series (Allais et al., 2018; Bonneau et al., 2018) (Scheme 2).

In this step, **2a**, **2b**, or **2c** and hydrogen peroxide were fully consumed, and Amberlyst beads were filtered off. Moreover, due to their low boiling point, formic acid by-products and solvents (*i.e.*, ethanol and water) were readily removed under vacuum. Consequently, purification over silica gel was not required, and the desired **3a**, **3b,** and **3c** were obtained in excellent yields (>99%, 93%, and 97%, respectively).

### Step 3: activation (and protection)

The primary and secondary alcohols of **3a** were then activated and protected by tosylation and acetylation, respectively, through a one-pot two-step procedure. A small excess of tosyl chloride was required to warrant the total conversion of **3a** and limit residual reagent in the medium after extraction of **4a** (77% overall yield without requiring purification over silica gel). To achieve **4d**, the direct addition of acetic anhydride into the medium was performed, resulting in a global yield of 77%, meaning a quantitative acetylation of the tosylated intermediate (Scheme 2).

Activation of the primary alcohol of the lactone **3b** proved to be more difficult than activation of the unprotected **3a**. Indeed, the tosylation step never reached completion even with a longer reaction time (24 h instead of 2 h), higher dilution (0.5 mol.L^−1^ instead of 1 mol.L^−1^), or with the use of a co-solvent (1 mol.L^−1^ in Cyrene^®^, with 3 equiv. of pyridine). The tosylated product **4b** was isolated after purification over silica gel with a maximum of 50% yield. As mechanochemical tosylation was described in the literature as an alternative sustainable methodology (Kazemi et al., 2007), an assay in a mortar led to a slight increase in conversion from 50% to 77%, as determined by ^1^H NMR. However, although trials performed in a ball grinder showed complete conversion of the starting material, the degradation of the desired product was observed. Thus, mesylation was attempted under solvent conditions, and TLC monitoring of the reaction revealed a total conversion of the starting material after only 2 h. After treatment, an 88% yield of the desired product **4e** was isolated without the need for a specific purification step (Scheme 2). Successful activation of the primary alcohol of the lactone **3c** was achieved by tosylation under the same conditions as previously described, leading to **4c** in a 74% yield (Scheme 2).

### Steps 4 and 5: iodination and Bernet–Vasella reaction

Tosylated **4d** was then substituted by an iodine in the presence of sodium iodide, and subsequently, a Bernet–Vasella reaction was performed to provide the fully deprotected carboxylic acid with excellent yields (Bernet and Vasella, 1979) (Scheme 2). Interestingly, Wang and Nugent (2007) have previously reported a one-pot, two-step iodination and Bernet–Vasella procedure. Following their strategy, the one-pot two-step procedure led to an even higher global crude yield (*e.g.*, 98% vs. 78% for the two steps). The Bernet–Vasella reaction was commonly performed in THF, a highly toxic solvent (Florent et al., 1987). At least one example reported using green *n-*propanol as the solvent (Aspinall et al., 1984). Unfortunately, compounds of the **4** series proved poorly soluble in *n-*propanol. Alternatively, the bio-based cyclopentyl methyl ether (CPME), a green substituent of THF, has been used successfully for the conversion of **4d**. However, the final procedure used acetone as the solvent instead of CPME, as the latter proved to be a poor solvent for **4b**. Excellent crude yields were achieved for **5a**, **5b,** and **5c** (91%, 95%, and 98%, respectively). Note that the workup of the Bernet–Vasella reaction, consisting of filtration over a Celite^®^ pad and extraction, led to the **5** series with very few impurities. Nevertheless, purification over silica gel can be performed to obtain pure **5a**, **5c,** and **5d** in 55%, 78%, and 89% yields, respectively (Scheme 2). The highly hydrophilic **5a** would require alternative purification technology than flash chromatography over silica gel.

### Step 6: cross-metathesis homologation

With the different compounds in hand, the elongation of the fatty acid chain was investigated using cross-metathesis. This methodology allows the introduction of various chain lengths starting from the same precursor. However, because (*R*)-3-hydroxydecanoic acid has recently been reported as a very powerful elicitor, we will focus on the C-10 chain length (Schellenberger et al., 2021).

**SCHEME 3:**
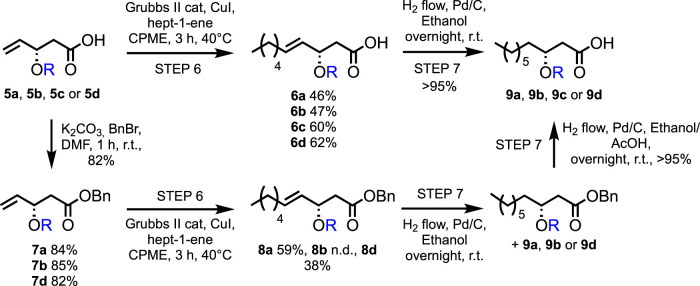
Synthesis of the **9** series through a cross-metathesis reaction and hydrogenation.

**FIGURE 3:**
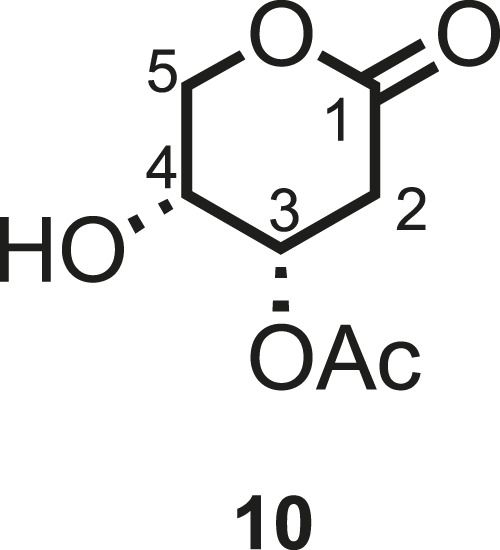
Hypothetical structure for the by-product **10** isolated with **5d**.

Various conditions (catalyst loading and addition of catalyst by portions or through syringe pump, in dry CPME or methylene chloride, with different concentrations) have been tested for Grubbs II-catalyzed cross-metathesis reactions between pure **5d** and hept-1-ene (Scheme 3; Table 1). Grubbs II catalyst has been chosen due to its high stability to air and humidity, allowing simpler handling, in addition to its high efficacy in catalyzing the metathesis reaction. In all cases, a catalytic amount of copper(I) iodide was added to the medium as it was proved to have a beneficial effect on the yield of olefin cross-metathesis (Voigtritter et al., 2011). The continuous addition of 4.8 mol% of Grubbs II catalyst through a syringe pump has led to the best yield in **6d** (62%, Table 1, entry 6), similar to what was reported by Menhour et al. (65%) (Menhour et al., 2016). Note that the starting material was not fully consumed. Unfortunately, its recovery was not possible due to the concomitant elution of a contaminating by-product. A tentative structure elucidation based on the ^1^H, ^13^C, and 2D spectra led to compound **10** (Figure 3).

**TABLE 1:** Summary of the cross-metathesis reactions on **5d** with hept-1-ene.

Entry	Concentration (mol.L^−1^)	Grubbs II cat. equiv. (mol%)	Addition mode for Grubbs II cat.	Yield (%)
1^a^	0.1	4.8	1 portion	28
2^a^	0.1	2.9	3 portions	45
3^a^	0.1	4.8	10 portions	34
4^a^	0.1	2,4	Continuous addition^c^	30
5^b^	0.1	2,4	Continuous addition^c^	38
6	0.1	4.8	Continuous addition^c^	62
7	0.25	2.4	Continuous addition^c^	48
8	0.25	4.8	Continuous addition^c^	56

^a^ Hydrogenation was directly performed after cross-metathesis homologation. Reported yield for compound **6d**.

^b^ Reaction was performed in methylene chloride.

^c^ Grubbs catalyst was dissolved in 2 mL of CPME and added through a syringe pump over 1 h (flow: 2 mL.h^−1^).

Cross-metathesis between **5b** and hept-1-ene was performed following the best conditions obtained for **5d** (Table 1, entry 6), leading to **6b** in a 47% yield (Scheme 3). Decreasing the catalyst loading by two-fold caused only a slight decrease in yield (41%), but increasing the concentration to 0.25 mol.L^−1^ was more influential with a drop of 12 points and 15 points in yield with Grubbs II catalyst loading of 4.8 mol% and 2.4 mol%, respectively. It is worth mentioning that the hydrogenation was not performed directly after the cross-metathesis to allow the recovery of starting material **5b**. This was possible for the diluted media (*i.e.*, 0.1 mol.L^−1^), and *ca.* 40% of the starting material was recovered without any detectable by-product, whereas in more concentrated experiments, a small amount of the same kind of impurity as for **5d** was observed by ^1^H NMR analysis. When using **5a**, contrary to what was observed from studies on **5d** and **5b**, reducing the amount of catalyst led to a higher yield of **6a** (from 24% to 46%). After purification, 60% of the desired compound **6c** and 38% of starting material **5c** were recovered.

To investigate the influence of the presence of the free carboxylic acid on the cross-metathesis reaction, benzylation of the **5** series was performed, leading to the **7** series, followed by cross-metathesis with hept-1-ene (Scheme 3). Cross-metathesis of the protected ester **7a** provided a higher yield (**8a**, 59%) than the free carboxylic acid **5a** (**6a**, 46%). In contrast, **8d** was only obtained in a 38% yield, and 53% of the starting material **7d** was recovered, whereas **6d** reached a 62% yield from **5d**.

The cross-metathesis yields reported in this article are consistent with values reported in the literature for similar reactions (Menhour et al., 2016). The presence of a free carboxylic acid group does not seem to highly impact the reactivity of the Grubbs II catalyst, as no clear trend was observed by comparison with unprotected *versus* benzylated esters. Increasing the concentration from 0.1 to 0.25 mol.L^−1^ also has a predominantly positive effect on yields. The presence of an alcohol moiety in position 3 and a bulky group (benzyl) have a negative impact on the yields, which did not exceed 50%. In comparison, smaller substituents (*i.e.*, acetyl and ethyl) allowed 60% yields to be achieved.

### Step 7: hydrogenation

Finally, a palladium-catalyzed hydrogenation is required to achieve the synthesis of HAAs. Whereas the reduction of the double bond was completed overnight in all cases, removing the 3-*O*-benzyl moiety proved more challenging. However, this drawback allowed achieving the unexpected analog **9b** in a 72% yield. Acetic acid was added to the ethanolic reaction medium to successfully achieve complete benzyl cleavage. All reactions proceeded with yields higher than 95%.

## Compliance with green chemistry

First, **LGO** was chosen as the starting material not only because it is a biosourced chemical but also because it is obtained through flash-pyrolysis of cellulosic wastes through the Furacell process™, an energy-neutral approach (Circa group et al., 2023). Second, particular attention was focused on the nature of the solvents and their quantity. Indeed, no halogen-containing solvent was used, and toxic diethyl ether was replaced by CPME, which is characterized by a low formation of peroxide, a high boiling point (106°C), and renewable resource sourcing (Merck, 2019). In addition, acetone, ethyl acetate, benzyl alcohol, and ethanol can be produced from renewable feedstocks (Weizmann and Rosenfeld, 1937; Pattanaik and Mandalia, 2011; Redl et al., 2017; Liu et al., 2020; Flaiz et al., 2021; Melendez et al. 2022). Whenever possible, organic solvents were avoided (*e.g.*, STEP 2), or the substrate concentration was as high as 1 mol.L^−1^ to reduce their consumption (STEPS 3–5). It is worth mentioning that, in the case of benzyl alcohol, its recyclability was successfully achieved through a short-path distillation. To further comply with the principles of green chemistry, toxic reagents were avoided whenever possible. For example, a two-step procedure (tosylation followed by iodination) was preferred to the more direct Appel reaction to avoid the use of the toxic triphenylphosphine and limit waste (*i.e.*, triphenylphosphine oxide). Finally, the synthetic pathway described herein requires a minimum of purification steps (*i.e.*, chromatography over silica) to access the desired compounds. Indeed, over seven steps (*i.e.*, oxa-Michael addition, Baeyer–Villiger oxidation, activation, iodination, Bernet–Vasella reaction, cross-metathesis, and hydrogenation), only two to three purifications over silica gel were required depending on the targeted product. The key purification was systematically performed after the cross-metathesis homologation to remove by-products and recover both the desired product and the starting material.

The ecological performance of each step has been calculated using green metrics to assess their impact. Several methods exist to determine such metrics (Van Aken et al., 2006). Among them, a very simple one is the process mass intensity (PMI) ratio, which establishes a ratio between the summed masses of all products entering into a reaction (water excepted) and the mass of recovered target(s). EcoScale, another approach designed by Van Aken et al., evaluates six criteria (*i.e.*, yield, price, safety, technical setup, temperature/time, and workup/purification) and gives penalty points deduced from a starting score of 100 (Van Aken et al., 2006). This method has been specifically designed for laboratory scale and allows ranking the reactions in three categories: 1) >75, excellent; 2) >50, acceptable; and 3) <50, inadequate. Combining these two methods led to an overview in terms of waste generation and the safety/difficulty of each reaction without performing complex calculations, as in the case of an in-depth life cycle assessment (LCA). PMI and EcoScale scores were calculated for each step of the process to evaluate which pathway was the eco-friendliest. It is important to mention that the PMI calculations did not consider treatment after the reaction due to the lack of information in the literature on the volume of solvents used during extraction and purification procedures.

For our process (Supplementary Table S6.1), all PMI ratios were significantly low (<15), except for the cross-metathesis step (between 31 and 72), particularly due to the high dilution (C = 0.1 mol.L^−1^). Indeed, when the concentration was increased from 0.1 to 0.25 mol.L^−1^ for the conversion of **7b** into **8b**, the PMI ratio was logically divided 2.5-fold, leading to a very acceptable value. Moreover, recyclability through distillation of CPME (bp 106°C) and unreacted hept-1-ene (bp 93°C) was not addressed here but could be considered an appreciable improvement. Whatever the synthetic strategy chosen, the average PMI ratio is less than 20, which is very good for the synthesis of a fine chemical. Generally speaking, our approach results in high to excellent EcoScale values for most steps thanks to the safety of reactants, the simplicity of treatments, the few purifications, and the good to excellent yields. One notable exception is the cross-metathesis homologation. Indeed, the moderate yields of these reactions, in addition to the use of an expensive catalyst and toxic hept-1-ene, led to inadequate reaction conditions following Van Aken’s definition. Overall, our seven-step synthetic pathway reaches acceptable conditions with average EcoScale scores above 60 (Table 2).

**TABLE 2:** Average PMI and EcoScale scores for the various pathways explored in this work compared with Bauer’s and Menhour’s procedures.

This work	EcoScale	PMI
Average score for **9a** (acidic route)	72.1	18
Average score for **9a** (benzyl route)	64.9	9.4
Average score for **9d** (acidic route)	73.5	16.9/11.3
Average score for **9d** (benzyl route)	71.2	10.5
Average score for **9b**	70.1	16.9
Average score for **9c**	71.4	9.8
Bauer’s procedure		
Average score for free carboxylic acid and free alcohol	57	12.2
Average score for free carboxylic acid and protected alcohol	61.1	14.5
Average score for protected carboxylic acid and free alcohol	53.8	48.2
Menhour’s procedure		
Average score for free carboxylic acid and protected alcohol (1 to 5)	43.2	90.6
Average score for protected carboxylic acid and alcohol (1 to 4)	36.1	31.4

Surprisingly, pathways involving the benzyl protection achieved better PMI ratios than the direct route. That can be mainly explained by the increase in yield for the cross-metathesis homologation in the alcohol series (**7a** compared to **5a**) and the high recovery of the starting material in the acetyl series (**7d** compared to **5d**). However, the impact on the average EcoScale score is slightly negative for the acetyl derivatives; the score is more impacted for the alcohol derivatives (Table 2).

In order to assess the ecological performance of the developed process, the aforementioned EcoScale and PMI results were benchmarked against those of the already published research works.

Even though Bauer and co-workers have used toxic solvents such as chloroform, methylene chloride, or methanol (Bauer et al., 2006), their EcoScale score remains acceptable to good due to the high yields of each reaction step (>78%), few purification steps, and easy setup (except for the selective hydrogenation) and workups. However, the PMI ratio shows that this synthetic methodology generates an unreasonable amount of waste, especially during the desilylation, although the latter was carried out at a multigram scale (Table 2).

With Menhour et al.’s strategy (Supplementary Table S6.3) (Menhour et al., 2016), the PMI ratio remains acceptable as long as the reactions were performed on a “convenient” scale but drastically increases when performed on 1 mmol (cross-metathesis followed by hydrogenation), and even more on 0.1 mmol (deprotection). This shows the importance of working in concentrated conditions to limit the environmental footprint, which becomes more and more difficult when the scale is reduced. In contrast, the EcoScale score was poor for the three first steps due to toxic solvents and reagents (*e.g.*, diethyl ether and acrolein) and low yield (*i.e.*, enantioselective acetylation), whereas good values were achieved for the two last steps.

To summarize, considering PMI and EcoScale scores, even with more steps, our synthetic procedure remains more sustainable than the previously reported procedures. The desired HAAs were obtained with global yields ranging from 24% to 36%. The implementation of this new pathway is very easy (common setup and materials, no need for anhydrous solvent, etc.) and has been conducted on a (multi)gram scale. The synthetized HAAs could be used directly in biological assays to evaluate their impact on stimulating plant immune systems. They could also be used in the HASP developed by Bauer et al. to achieve functionalized rhamnolipids.

## Data Availability

The original contributions presented in the study are included in the article/Supplementary Material, further inquiries can be directed to the corresponding authors. In addition, NMR FID raw files can be found at DOI: 10.57745/D1G0IK.
