# Supplementary material for: Green synthesis of (R)-3-hydroxy-decanoic acid and analogs from levoglucosenone: a novel access to the fatty acid moiety of rhamnolipids
Source: Front Chem. 2024 Apr 19;12:1362878. doi: 10.3389/fchem.2024.1362878 (PMC11066284; doi:10.3389/fchem.2024.1362878)
Supplement: Supplementary file 1 [file DataSheet1.PDF]

Green synthesis of (*R*)-3-hydroxy-decanoic acid and analogues from Levoglucosenone: A novel access to the fatty acid moiety of rhamnolipids

Enzo Petracco, Amandine L. Flourat,* Marie-Charlotte Belhomme, Stéphanie Castex, Fanny Brunissen, Fanny Brunois, Aurélien A. M. Peru, Florent Allais* and Arnaud Haudrechy*

Corresponding authors: amandine.flourat@agroparistech.fr, florent.allais@agroparistech.fr, arnaud.haudrechy@univ-reims.fr

Number of pages: 93

Number of figures: 68

Number of tables: 27

Number of schemes: 1

Summary

1. Materials 3
2. Experimental Procedures 3
2.1. Oxa-Michael addition 3
2.1.1. Addition of water[1] 3
2.1.2. Addition of benzyl alcohol[2] 3
2.1.3. Addition of ethanol 4
2.2. General procedure for Baeyer-Villiger oxidation[3] 11
2.3. OH protection 18
2.3.1. General procedure for tosylation or mesylation of HBO-OH, HBO-OBn and HBO-OEt 18
2.3.2. Tosylation of HBO-OH and acetylation of secondary alcohol 18
2.4. General procedure for iodination 30
2.5. General procedure for Bernet-Vasella reaction 39
2.6. One-pot procedure for iodination and Bernet-Vasella reaction. 39
2.7. Carboxylic acid protection 48
2.8. General procedure for cross-metathesis 55
2.9. General procedure for hydrogenation 71
3. Design of experiment for optimization of ethanol addition on LGO 80
4. Structure elucidation of the unknown compound 84
5. EcoScale calculations 85
5.1. EcoScale of this work 85
5.2. EcoScale of Bauer's procedure 87
5.3. EcoScale of Menhour's procedure 89
6. Summary of PMI and EcoScale 91
References 93

1. Materials

Levoglucosenone was kindly provided for free by Circa Group. MilliQ grade water was produced by an Integral 5, Merck-Millipore. Potassium phosphate tribasic (K_3PO_4), sulfuric acid (H_2SO_4), anhydrous magnesium sulfate ($MgSO_4$), triethylamine, pyridine, hydrogen peroxide (H_2O_2) and potassium carbonate (K_2CO_3) were purchased from Fischer Scientific. Amberlyst 15 IR wet, mesyl chloride, sodium iodide (NaI), sodium thiosulfate ($Na_2S_2O_3$), zinc, benzyl bromide, Grubbs II catalyst M204, copper iodide (CuI), cyclopentyl methyl ether (CPME), palladium on charcoal were purchased from Sigma Aldrich. Benzyl alcohol, tosyl chloride and hept-1-ene were purchased from TCI. Celite® 345R, acetic anhydride and solvents were purchased from VWR-Avantor sciences. Deuterated solvents were purchased from Eurisotop. DMF was dried on a mBraun SPS 800 system. Flash purifications were performed on a Flash XS, Interchim, equipped with prepacked PF-30SI-HP (30 µm silica gel) columns, Interchim. NMR analyses were recorded on a Bruker Fourier 300. ^{1}H NMR spectra of samples were recorded at 300 MHz; chemical shifts were reported in parts per million relative to the residual solvent peak (δ = 7.26 for $CDCl_3$ and 2.50 for DMSO-d_6). ^{13}C NMR spectra of samples were recorded at 75 MHz, chemical shifts were reported in parts per million relative to the residual solvent peak (δ = 77.16 for $CDCl_3$ and 39.52 for $(CD_3)_2SO$). $[\alpha]_D$ were recorded on a polarimeter ADP410, Bellingham Stanley. High-resolution mass spectrometries were performed on an Agilent 1290 system, equipped with a 6545 Q-ToF mass spectrometer and a PDA UV detector. The source was equipped with a JetStream ESI probe operating at atmospheric pressure. Melting points were measured on a MP50, Mettler Toledo.

2. Experimental Procedures

2.1. Oxa-Michael addition

2.1.1. *Addition of water*[1]

Levoglucosenone (12.6 g, 100 mmol) was diluted in water (1.25 L), then K_3PO_4 (1.06 g, 5 mol%) was added. This solution was stirred at room temperature during 5 h. The reaction was neutralized to pH 7 with HCl 3M before concentration till approximatively 0.30 L. Aqueous layer was extracted with EtOAc (2*150 mL) to remove unreacted LGO and dimer by-product. Aqueous layer was concentrated till approximatively 50 mL, diluted in 200 mL of acetone and filtered over a pad of Celite® to remove salt. The filtrate was concentrated to dryness.

Levoglucosenone hydrate 2a, pale yellow oil (10.8 g, purity of 95% determined by ^{1}H NMR)

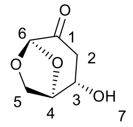

$[\alpha]_D^{23.6}$ = -196 (c 0.1, Ethanol). **^{1}H NMR** 5.40 (d, $J_{7/3}$ = 3.9 Hz, 1H, H7), 5.05 (d, $J_{6/5b}$ = 1.3 Hz, 1H, 6), 4.63 – 4.48 (m, 1H, H3), 4.16-4.06 (m, 1H, H5a), 3.97 (dd, $J_{5b/4}$ = 7.9 Hz, 1H, H5b), 3.75 (ddd, $J_{4/3}$ = 5.6 Hz, $J_{4/5a}$ = 0.5 Hz, 1H, H4), 2.92 (dd, $J_{2a/2b}$ = 16.9 Hz, $J_{2a/3}$ = 5.8 Hz, 1H, H2a), 2.22 – 2.09 (m, 1H, H2b) ppm. **^{13}C NMR** ((75 MHz, DMSO) δ 200.7 (C1), 100.4 (C6), 77.2 (C4), 68.9 (C3), 64.6 (C5), 40.5 (C2) ppm. No ionization was recorded from HRMS analysis.

2.1.2. *Addition of benzyl alcohol*[2]

Levoglucosenone (1.00 g, 7.9 mmol) and triethylamine (550 µL, 3.95 mmol, 0.5 equiv.) in benzyl alcohol (32 mL, 38 equiv.), were stirred overnight at room temperature. The reaction was stopped by evaporation of the triethylamine. Excess of benzyl alcohol was recovered by short path distillation (125 C, 15 mbar) (98% recovering). The crude product was purified over silica gel (Eluent: Cyclohexane / EtOAc: 90/10 to 70/30) to give **2b** (94 % yield).

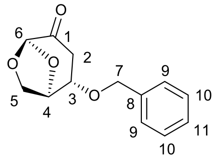

***O*-Benzyl Levoglucosenone 2b**, yellow oil (1.75 g, 94% yield)

[α]$_D^{23.8}$ = -164 (c 0.050, EtOAc). **^{1}H NMR** (300 MHz, $CDCl_3$) δ 7.55-7.09 (m, 3H, H9, H10, H11), 5.16 (s, 1H, H6), 4.77 (d, $J_{3/2\&3/4}$ = 5.3 Hz, 1H, H3), 4.68 – 4.49 (m, 2H, H7), 4.01 – 3.78 (m, 3H, H4, H5), 2.73 (dd, $J_{2a/2b}$ = 17.1 Hz, $J_{2a/3}$ = 5.7 Hz, 1H, H2a), 2.59 (d, 1H, H2b) ppm. **^{13}C NMR** (75 MHz, $CDCl_3$) δ 198.5 (C1), 137.3 (C8), 128.6 (C10), 127.8 (C9), 127.0 (C11), 101.3 (C6), 76.0 (C4), 75.0 (C3), 70.8 (C7), 65.2 (C5), 37.4 (C2) ppm. No ionization was recorded from HRMS analysis.

2.1.3. Addition of ethanol

Levoglucosenone (1.0 g, 7.9 mmol) and triethylamine (2.2 mL, 15.8 mmol, 2 equiv.) in ethanol (100 mL) were stirred during 72 h at room temperature. The reaction medium was concentrated. Then the crude product was purified over silica gel (cyclohexane:ethyl acetate 80/20 to 70/30).

***O*-Ethyl Levoglucosenone 2c**, colorless oil (1.16 g, 86% yield)

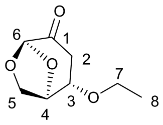

[α]$_D^{23.6}$ = -203 (c 0.020, EtOAc).**^{1}H NMR** (300 MHz, $CDCl_3$) δ 5.15 (s, 1H, H6), 4.78 (dq, $J_{3/4\&3/2a}$ = 5.6 Hz, $J_{3/2b}$= 1.8 Hz, 1H, H3), 4.07 – 3.71 (m, 3H, H4, H5), 3.56 (q, $J_{7/8}$ = 7.0 Hz, 2H, H7), 2.73 (dd, $J_{2a/2b}$ = 17.1 Hz, 1H, H2a), 2.55 (d, 1H, H2), 1.24 (t, 3H, H8) ppm. **^{13}C NMR** (75 MHz, $CDCl_3$) δ 198.6 (C1), 101.4 (C6), 77.4 (C4), 75.2 (C3), 65.3 (C5), 64.8 (C7), 37.6 (C2), 15.4 (C8) ppm. **HRMS** [M+H]$^+$ predicted 173.0809, found 173.0890.

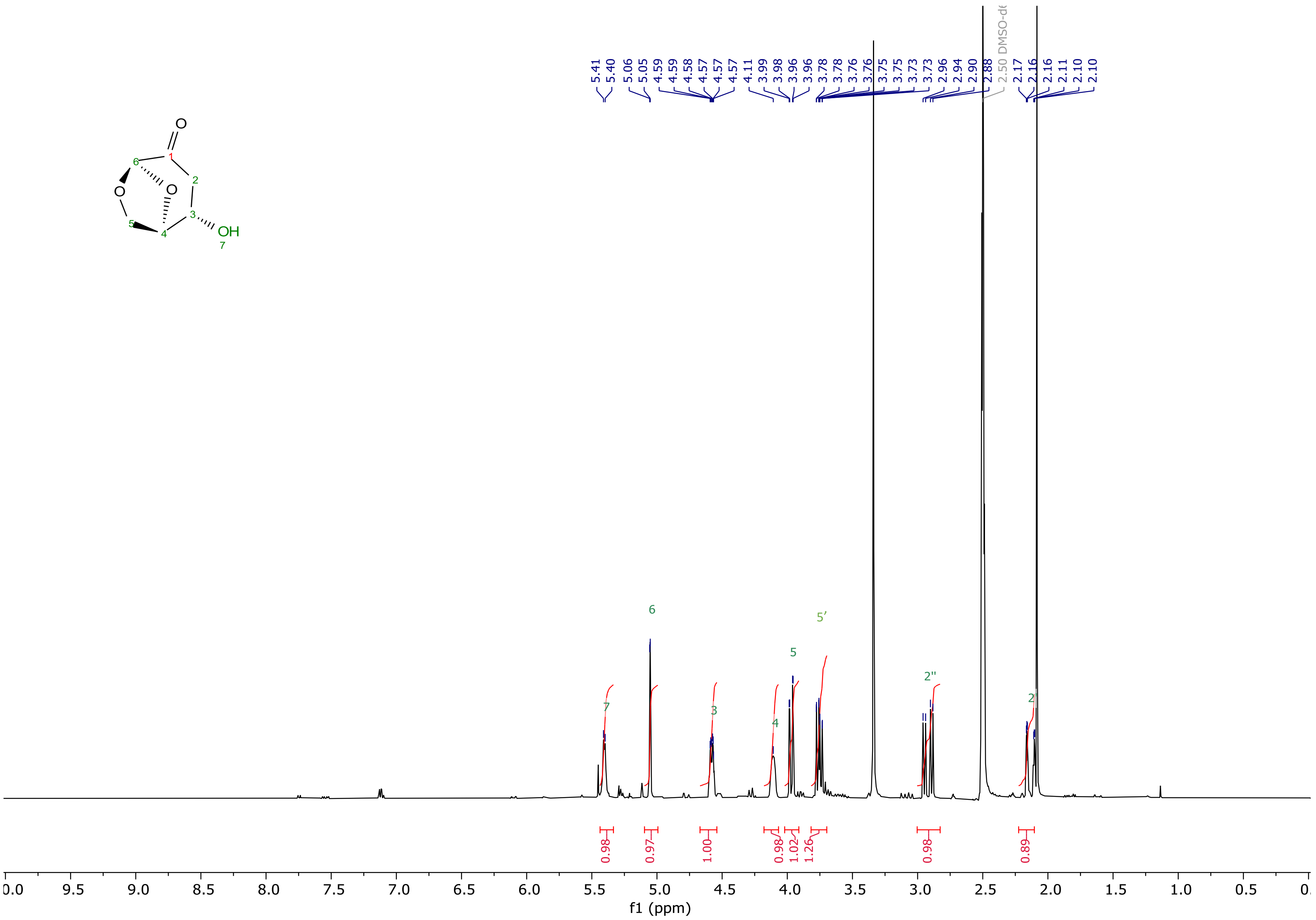

Figure 1: ^{1}H spectrum of compound ***2a***

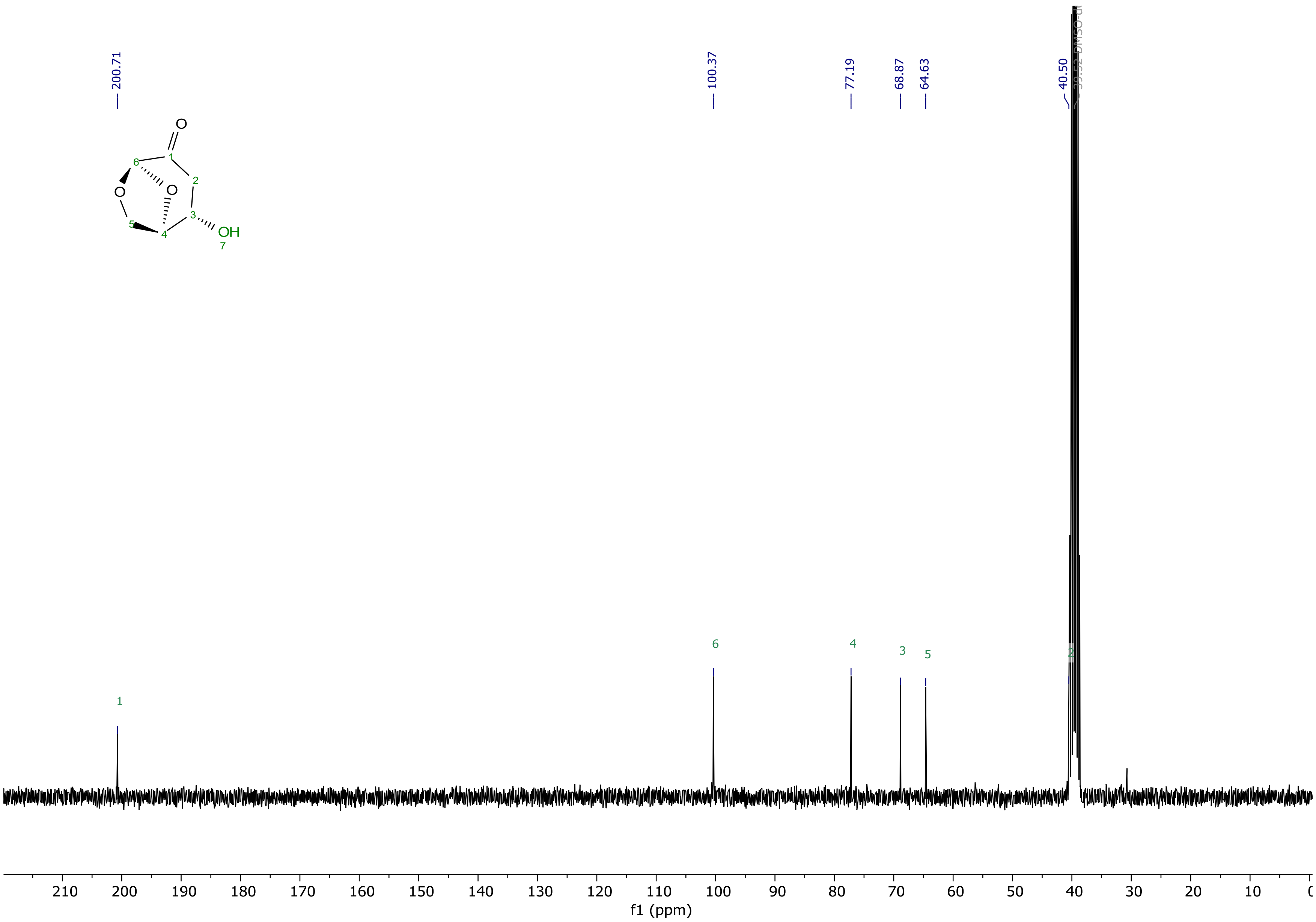

*Figure 2: ^{13}C NMR spectrum of compound **2a***

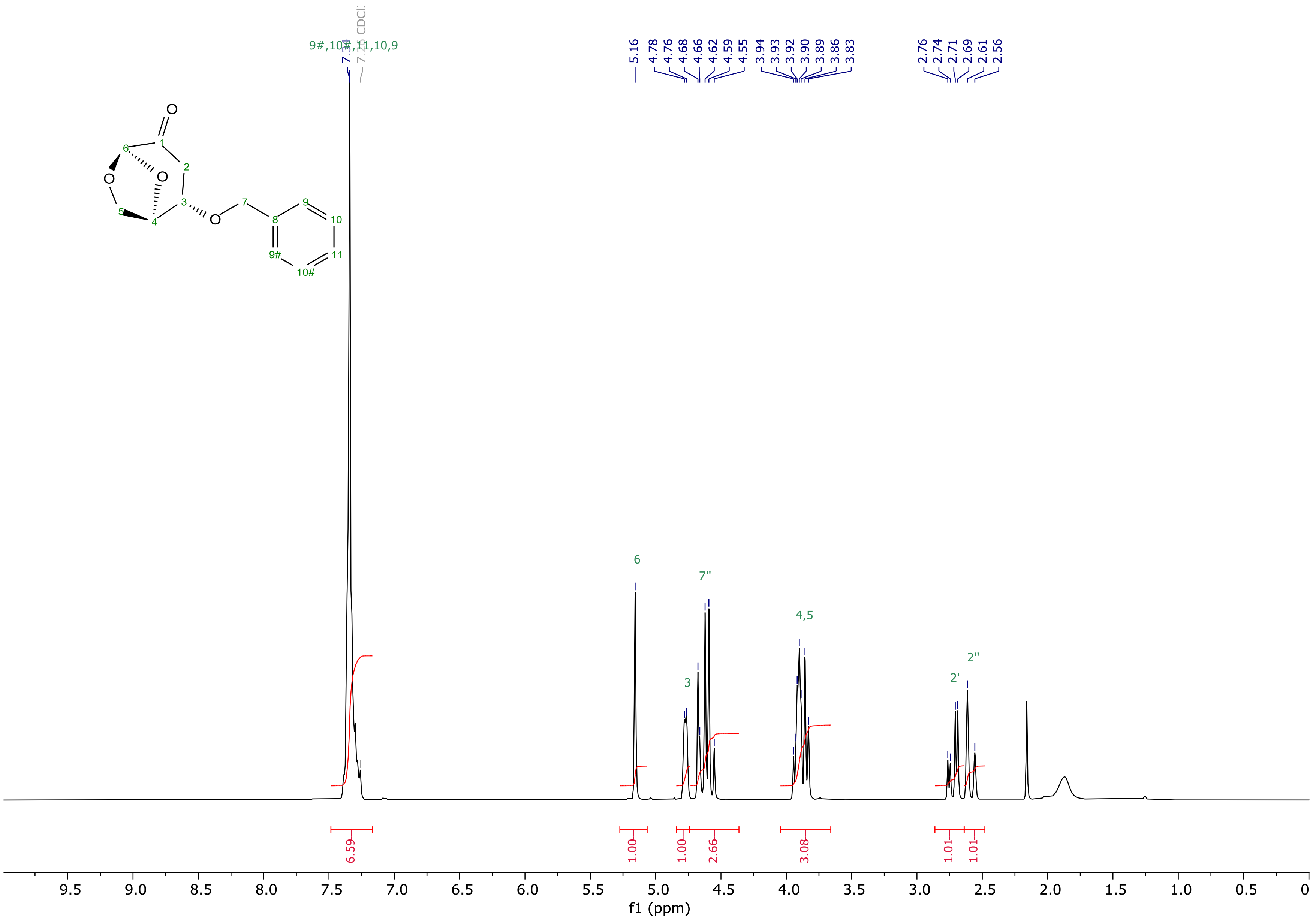

Figure 3: ^{1}H spectrum of compound ***2b***

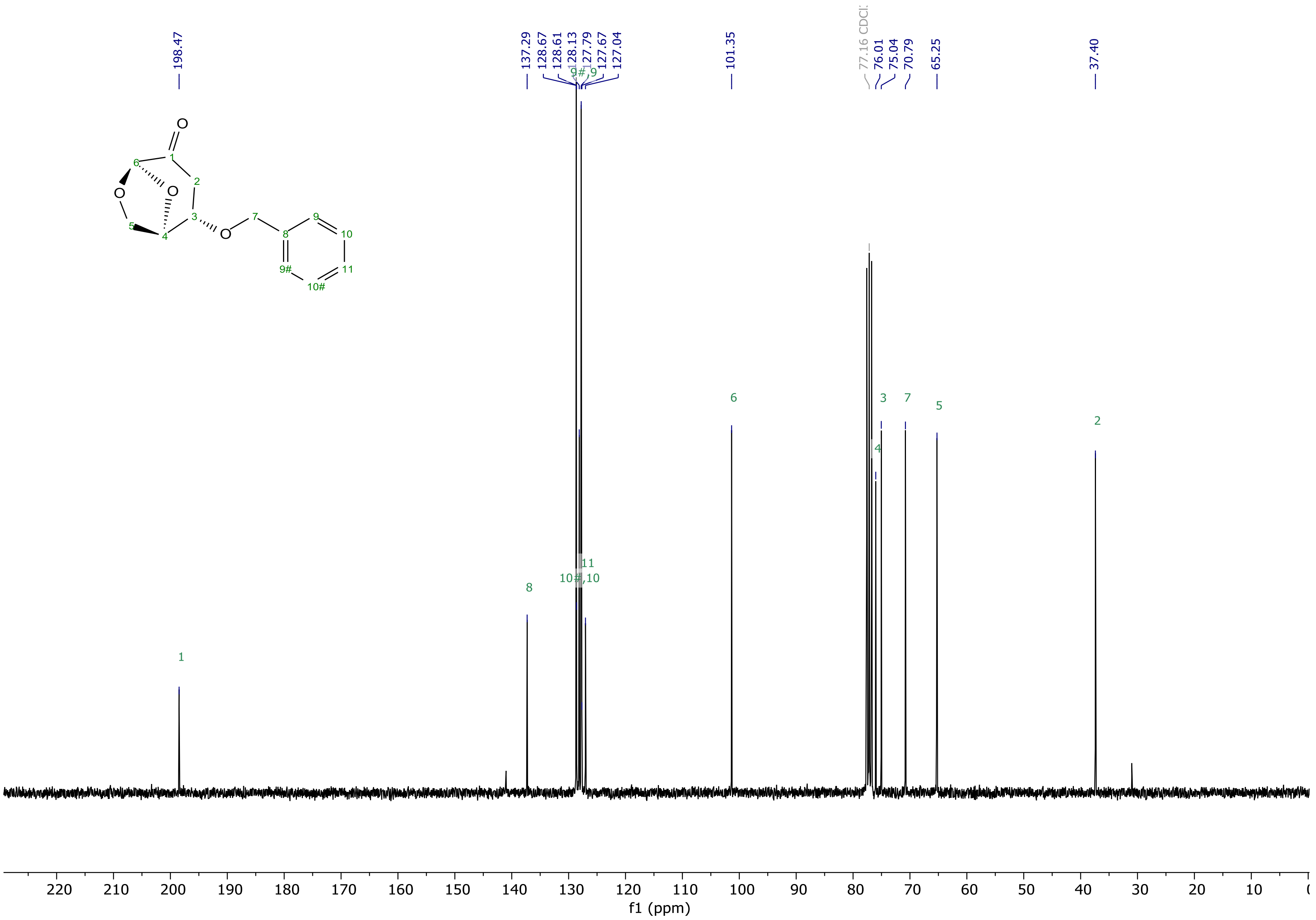

*Figure 4: ^{13}C NMR spectrum of compound **2b***

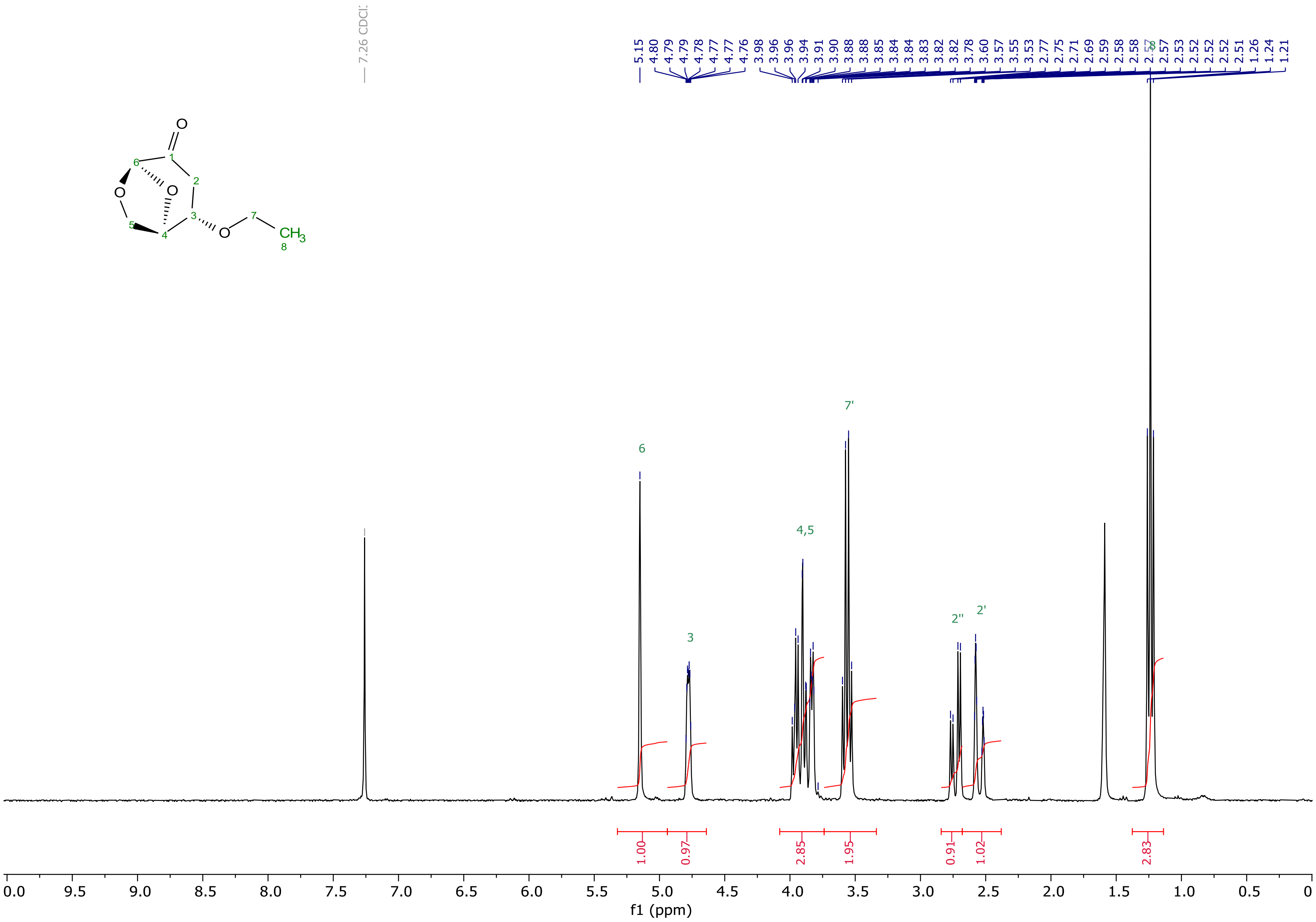

Figure 5: ^{1}H spectrum of compound **2c**

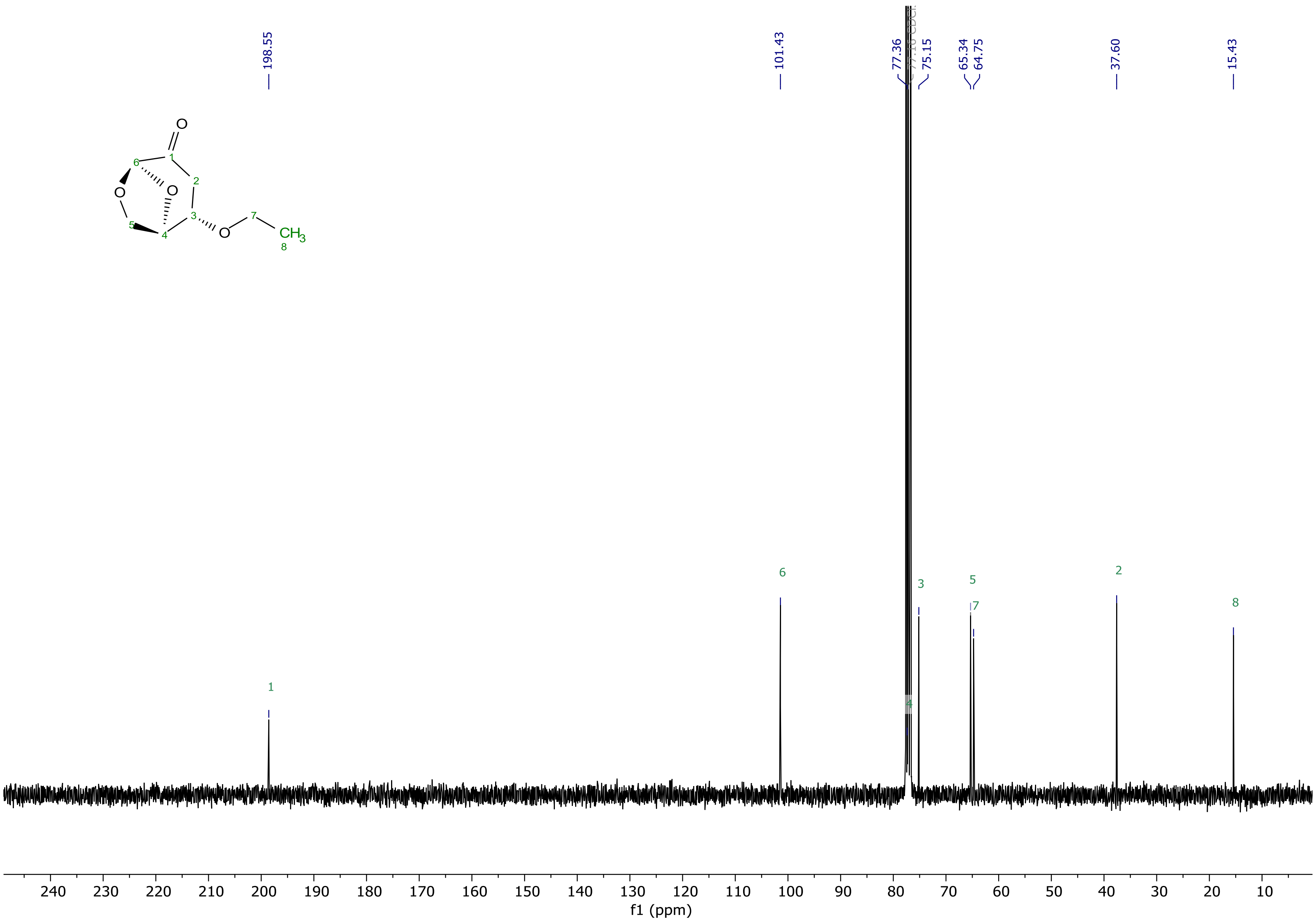

*Figure 6: ^{13}C NMR spectrum of compound **2c***

2.2. General procedure for Baeyer-Villiger oxidation[3a]

At 0°C, H_2O_2 30% w/v (1.1 equiv.) was added dropwise on the oxa-Michael addition product under gentle stirring. After the ice bath return to room temperature, the reaction was heated to 45°C. After 16 h of reaction, no H_2O_2 remains and the reaction was concentrated using a rotative evaporator set at 40°C. If necessary, hydrolysis of the formate intermediate was carried out using a spatula tip of Amberlyst 15IR in ethanol (approximatively 10 mL.g^{-1}). Amberlyst beads were removed by filtration and washed with ethanol. The resulting solution was concentrated to dryness.

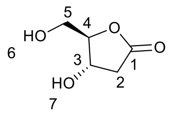

(*S*)-β-hydroxy-(*R*)-γ-hydroxymethyl-γ-butyrolactone 3a*,* colourless oil (9.87 g, 99% yield)
$[\alpha]_D^{24.2}$ = –20 (c 0.10, ethanol) [**$[\alpha]_D^{20}$** = –13.8 (water) lit.[3c]]. **^{1}H NMR** (300 MHz, DMSO) δ 5.50 (d, $J_{7/3}$ = 4.1 Hz, 1H, H7), 5.07 (t, $J_{6/5}$ = 5.4 Hz, 1H, H6), 4.32 – 4.20 (m, 2H, H3, H4), 3.54 (dt, $J_{5/4}$ = 3.7 Hz, 2H, H5), 2.81 (dd, $J_{2a/2b}$ = 17.7 Hz, $J_{2a/3}$ = 6.3 Hz, 1H, H2a), 2.28 – 2.16 (m, 1H, H2b) ppm. **^{13}C NMR** (75 MHz, DMSO) δ 176.2 (C1), 88.3 (C4), 67.8 (C3), 60.8 (C5), 38.0 (C2) ppm. **HRMS** $[M+H]^+$ predicted 133.0496, found 133.0489.

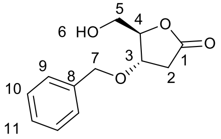

(*S*)-β-benzyloxy-(*R*)-γ-hydroxymethyl-γ-butyrolactone 3b*,* white solid (651 mg, 93% yield)
mp: 66°C. **$[\alpha]_D^{21}$** = -19.5 (c. 0.020, EtOAc) [**$[\alpha]_D^{20}$** = –19.25 (c 0.9, chloroform) lit.[3d]]. **^{1}H NMR** (300 MHz, $CDCl_3$) δ 7.50 – 7.27 (m, 6H, H9, H10, H11), 4.69 (s, 0.5H, H6), 4.65 – 4.46 (m, 3H, H3, H7), 4.31 (dt, $J_{4/5}$ = 7.0 Hz, $J_{4/3}$ = 7.0 Hz $J_{4/5}$ = 7.0 Hz 2.8 Hz, 1H, H4), 3.91 (dd, $J_{5a/5b}$ = 12.6 Hz, 1H, H5a), 3.69 (dd, 1H, H5b), 2.87 (dd, $J_{2a/2b}$ = 18.2 Hz, $J_{2a/3}$ = 7.1 Hz, 1H, H2a), 2.59 (dd, $J_{2b/3}$ = 3.1 Hz, 1H, H2b) ppm. **^{13}C NMR** (75 MHz, $CDCl_3$) δ 176.2 (C1), 137.1 (C8), 128.8 (C10), 128.3 (C11), 127.9 (C9), 85.7 (C4), 75.6 (C7), 71.6 (C3), 62.4 (C5), 36.1 (C2) ppm. **HRMS** $[M+Na]^+$ predicted 245.0784, found 245.0786.

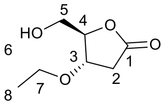

***(S)*-β-ethoxy-*(R)*-γ-hydroxymethyl-γ-butyrolactone 3c,** pale yellow oil (601 mg, 97% yield)
$[\alpha]_D^{21.7}$ = -18 (c. 0.050 EtOAc). **^{1}H NMR** (300 MHz, $CDCl_3$) δ 4.49 (q, $J_{4/5\&4/3}$ = 2.9 Hz, 1H, H4), 4.20 (dt, $J_{3/2a}$ = 6.4 Hz, 1H, H3), 3.95 (dd, $J_{5a/5b}$ = 12.5 Hz, 1H, H5a), 3.74 (dd, 1H, H5b), 3.49 (qd, $J_{7/8}$ = 6.9 Hz, $J_{7/3}$ =2.8 Hz, 2H, H7), 2.87 (dd, $J_{2a/2b}$ = 18. Hz, 1H, H2a), 2.55 (dd, $J_{2b/3}$ = 3.2 Hz, 1H, H2b), 1.21 (t, J = 7.0 Hz, 3H, 8).ppm. **^{13}C NMR**: (75 MHz, $CDCl_3$) δ 176.1 (C1), 85.7 (C4), 75.8 (C3), 65.0 (C7), 62.5 (C5), 36.1 (C2), 15.3 (C8) ppm. No ionization was recorded from HRMS analysis.

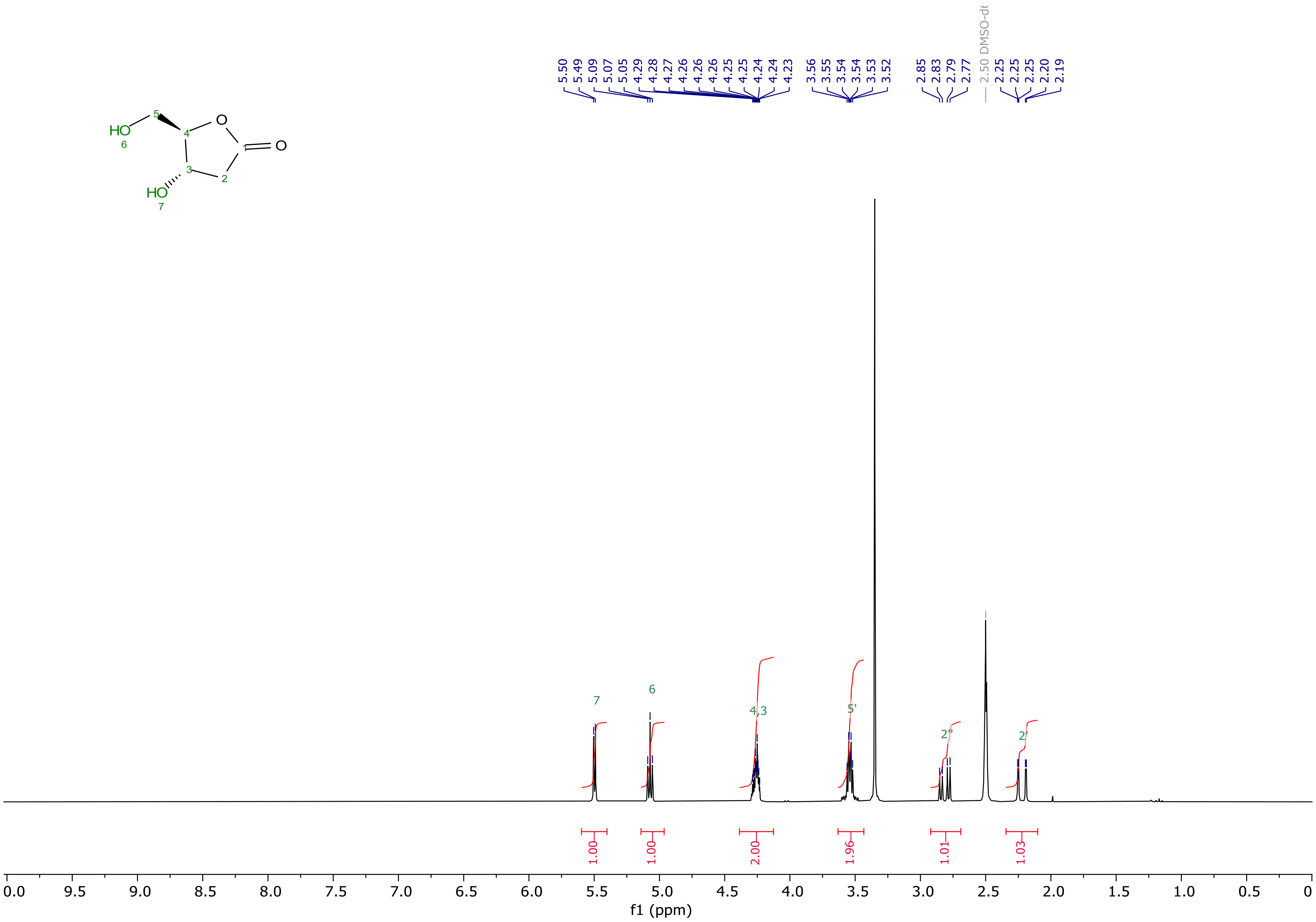

*Figure 7: ^{1}H spectrum of compound **3a***

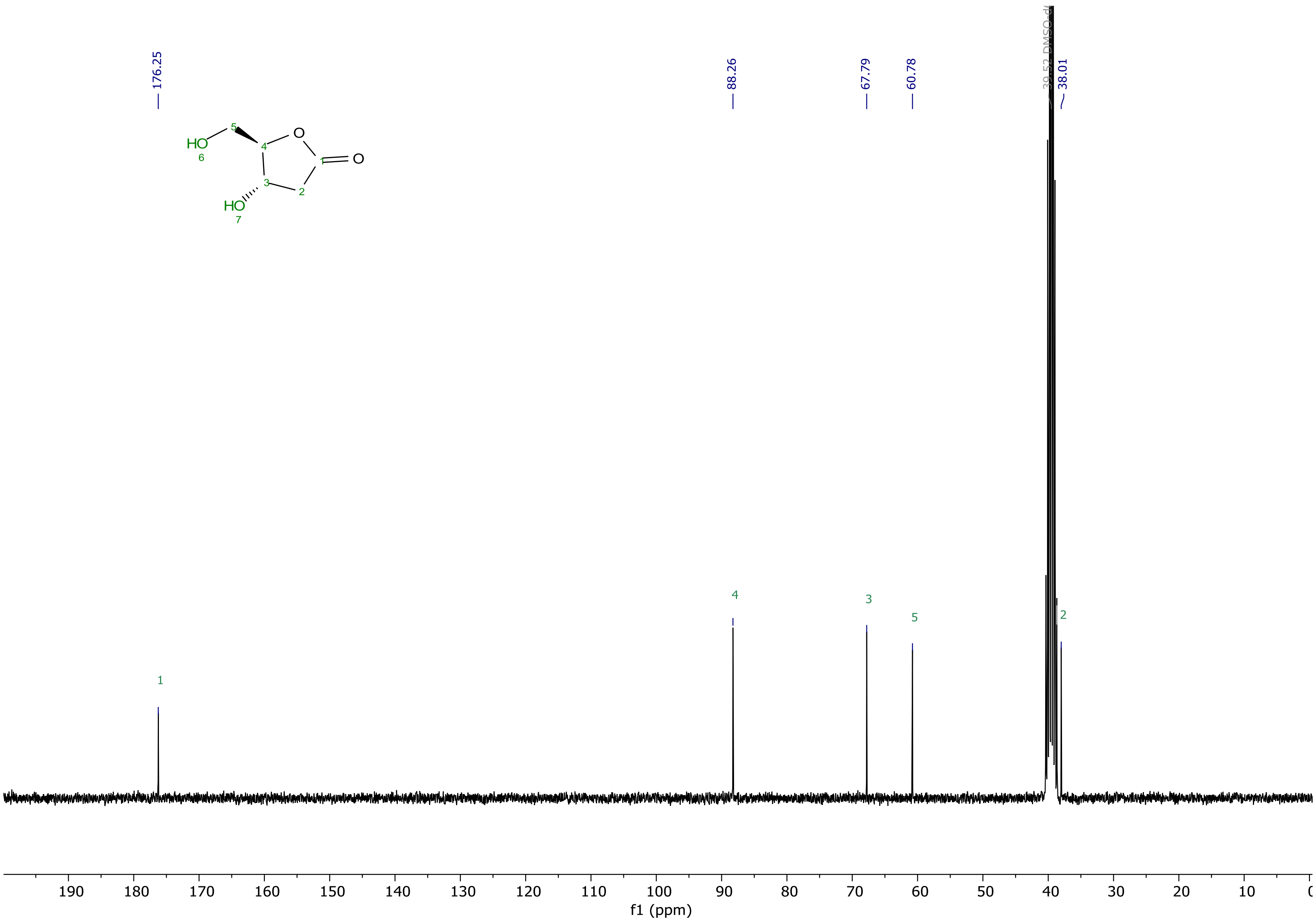

*Figure 8: ^{13}C NMR spectrum of compound **3a***

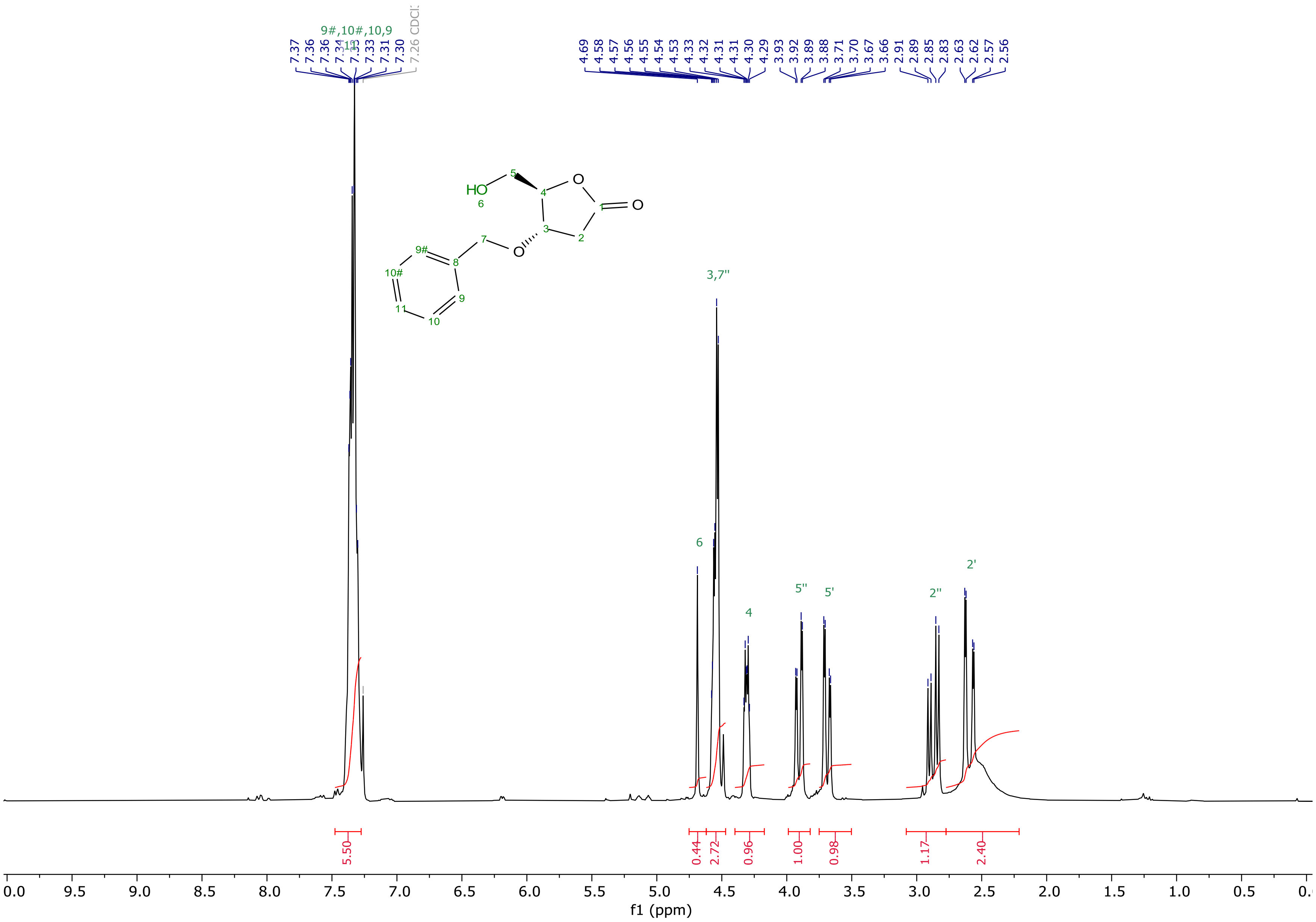

*Figure 9: ^{1}H spectrum of compound **3b***

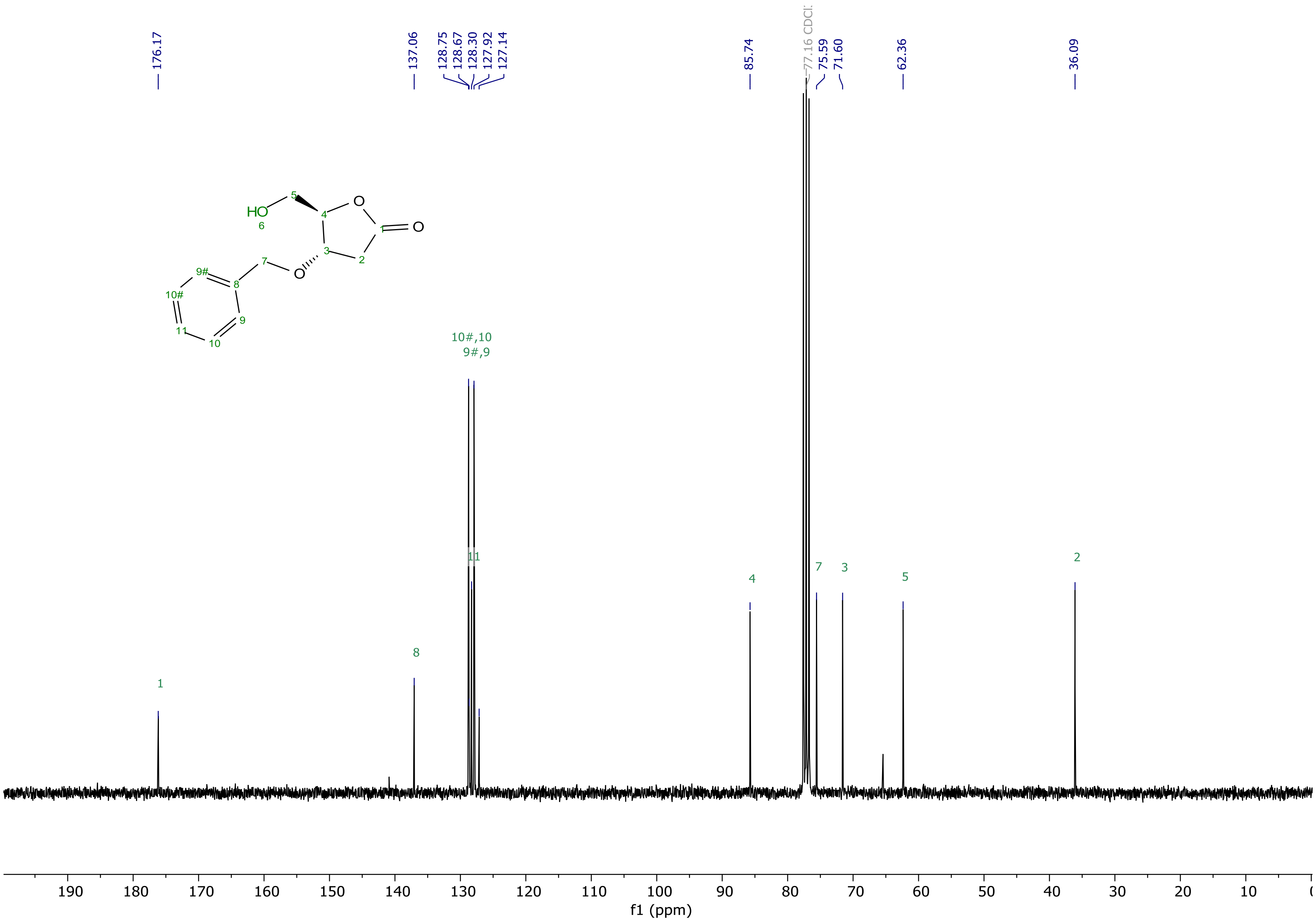

*Figure 10: ^{13}C NMR spectrum of compound **3b***

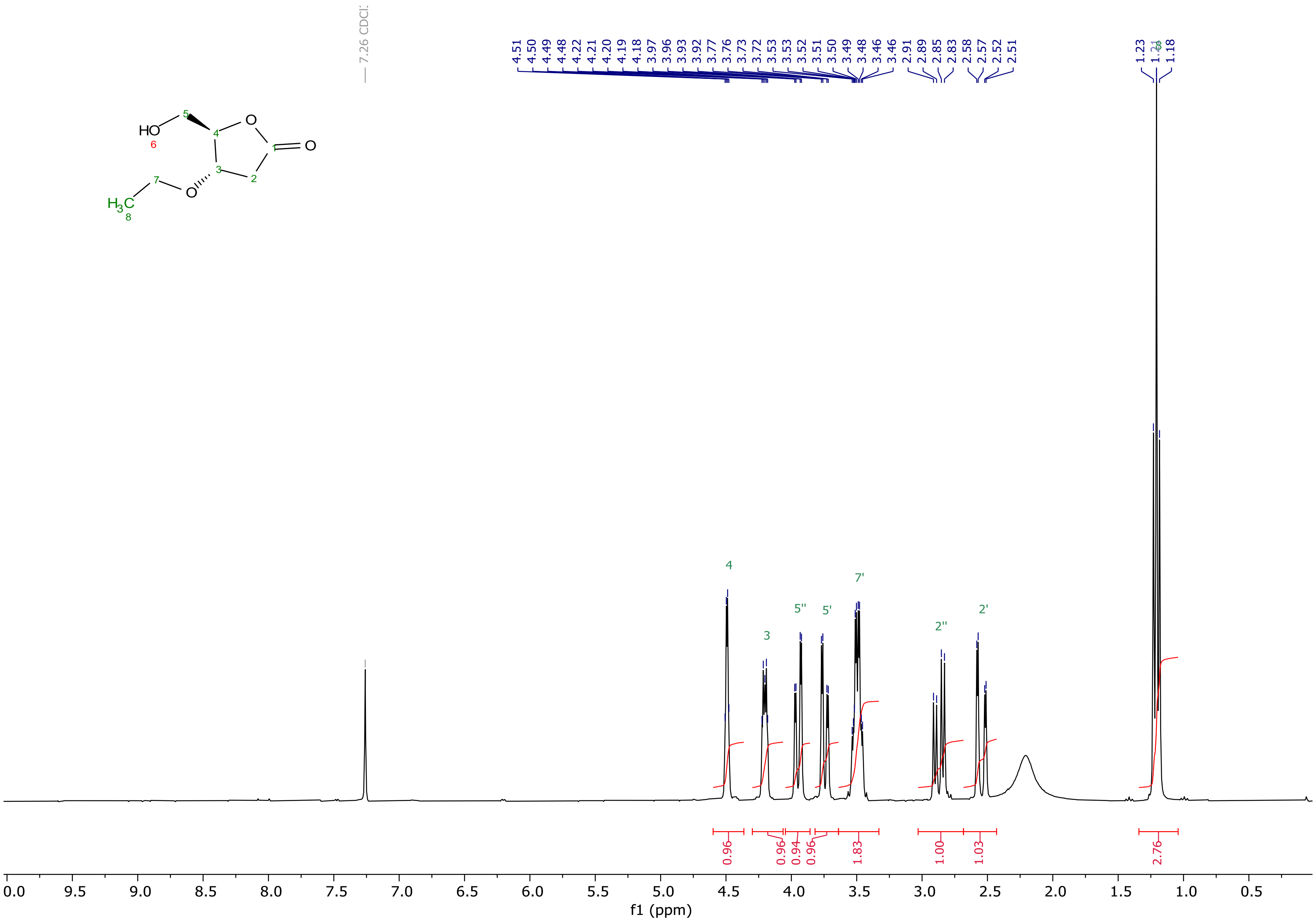

*Figure 11: ^{1}H spectrum of compound **3c***

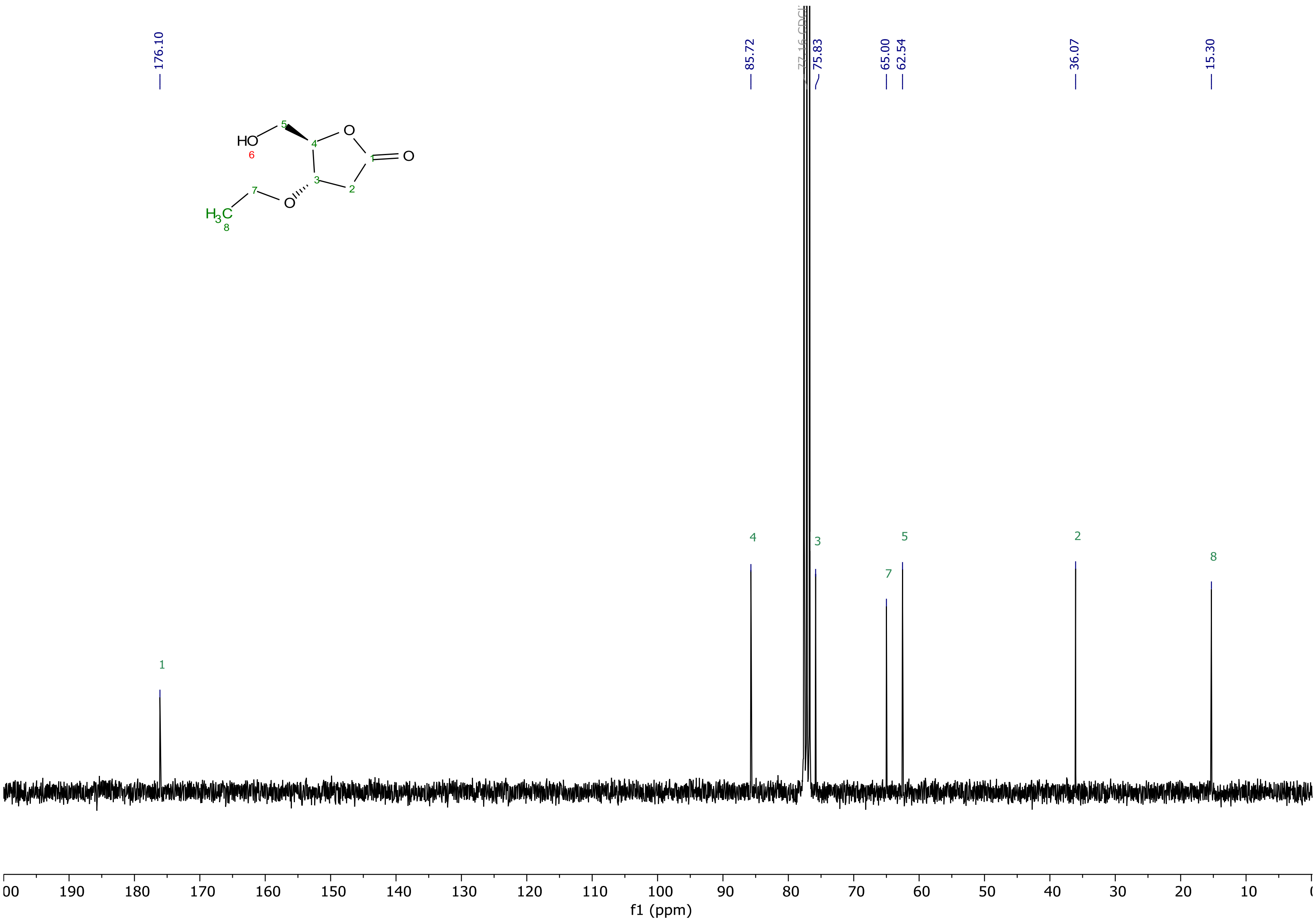

*Figure 12: ^{13}C NMR spectrum of compound **3c***

2.3. OH protection

2.3.1. General procedure for tosylation or mesylation of HBO-OH, HBO-OBn and HBO-OEt

The fonctionnalized hydroxymethyl-butyrolactone was solubilized in pyridine (1 mol.L^{-1}) before the addition of mesyl or tosyl chloride (1.25 equiv.). The reaction mixture was stirred for 2 h at room temperature. The reaction was quenched using 1 M HCl solution and the product was extracted twice using EtOAc. Organic layers were combined, dried over anhydrous $MgSO_4$, filtered and concentrated.

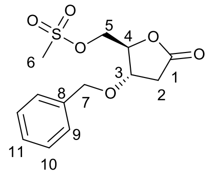

(*S*)-β-benzyloxy-(*R*)-γ-mesyloxymethyl-γ-butyrolactone 4e, colourless oil (154 mg, 88% yield)
$[\alpha]_D^{21.9}$ = +26.7 (c. 0.015 EtOAc). **^{1}H NMR** (300 MHz, $CDCl_3$) δ 7.42 – 7.27 (m, 5H, C9, C10, C11), 4.66 (q, $J_{4/3\&4/5}$ = 3.3 Hz, 1H, H4), 4.62 – 4.45 (m, 2H, H7), 4.44 – 4.22 (m, 3H, H3, H5), 3.00 (s, 3H, H6), 2.84 (dd, $J_{2a/2b}$ = 18.2 Hz, $J_{2a/3}$ = 7.2 Hz, 1H, H2a), 2.61 (dd, $J_{2b/3}$ = 3.6 Hz, 1H, H2b) ppm. **^{13}C NMR** (75 MHz, $CDCl_3$) δ 174.1 (C1), 136.6 (C8), 128.7 (C10), 128.3 (C11), 127.9 (C9), 81.7 (C4), 74.9 (C3), 71.8 (C7), 68.1 (C5), 37.5 (C6), 35.3 (C2) ppm. No ionization was recorded from HRMS analysis.

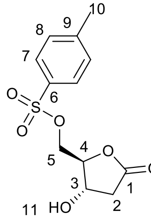

(*S*)-β-hydroxy-(*R*)-γ-tosyloxymethyl-γ-butyrolactone 4a, colourless oil (1.81 g, 77% yield)
$[\alpha]_D^{22.0}$ = +32.2 (c. 0.087 EtOAc). **^{1}H NMR** (300 MHz, $CDCl_3$) δ 7.76 (d, $J_{7/8}$ = 8.0 Hz, 2H, H7), 7.37 (d, 2H, H8), 4.74 – 4.44 (m, 2H, H3, H4), 4.41 – 4.07 (m, 2H, H5), 2.90 (dd, $J_{2a/2b}$ = 18.1 Hz, $J_{2a/3}$ = 7.1 Hz, 1H, H2a), 2.53 (dd, $J_{2b/3}$ = 3.5 Hz, 1H, H2b), 2.46 (s, 3H, H10), 2.19 (s, 1H, H11) ppm. **^{13}C NMR** (75 MHz, $CDCl_3$) δ 174.3 (C1), 145.9 (C9), 131.8 (C6), 130.3 (C8), 128.9 (C14), 128.5 (C15), 128.1 (C13), 128.0 (C7), 81.8 (C4), 72.0 (C11), 68.2 (C5), 35.4 (C2), 27.1 (C10) ppm. **HRMS** $[M+H]^+$ predicted 287.0584, found 287.0589.

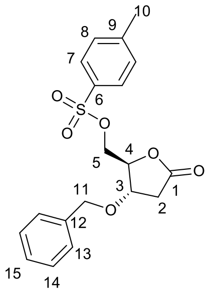

(*S*)-β-benzyloxy-(*R*)-γ-tosyloxymethyl-γ-butyrolactone 4b, white solid (670 mg, 50% yield)
mp: 72 C, **$[\alpha]_D^{22.2}$** = +34 (c. 0.051 EtOAc). **^{1}H NMR** (300 MHz, $CDCl_3$) δ 7.75 (d, $J_{7/8}$ = 8.1 Hz, 2H, H7), 7.44 – 7.26 (m, 7H, H8, H13, H14, H15), 4.63 – 4.41 (m, 3H, H4, H11), 4.36 – 4.25 (m, 1H, H3), 4.15 (dd, $J_{5a/5b}$ = 17.0 Hz, $J_{5/4}$ = 3.4 Hz, 2H, H5), 2.80 (dd, $J_{2a/2b}$ = 18.2 Hz, $J_{2a/3}$ = 7.1 Hz, 1H, H2a), 2.58 (dd, $J_{2b/3}$ = 3.1 Hz, 1H, H2b), 2.46 (s, 3H, H10) ppm **^{13}C NMR** (75 MHz, $CDCl_3$): δ 174.1 (C-1),145.8 (C-9), 136.7 (C-12), 132.0 (C-6), 130.3 (C-8), 128.8 (C-14), 128.5 (C-15), 128.1 (C-13), 128.0 (C-7), 81.8 (C-4), 75.2 (C-3), 71.9 (C-11), 68.2 (C-5), 35.4 (C-2), 21.8 (C10) ppm. **HRMS** $[M+H]^+$ predicted 377.1054, found 377.1049.

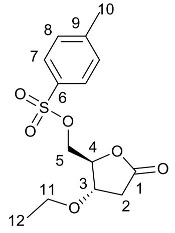

(*S*)-β-ethoxy-(*R*)-γ-tosyloxymethyl-γ-butyrolactone 4c, colourless oil (445 mg, 74% yield)
$[\alpha]_D^{22.0}$ = +31.0 (c. 0.043 EtOAc). **^{1}H NMR** (300 MHz, $CDCl_3$) δ 7.81 – 7.72 (m, 2H, H7), 7.37 ($d_{8/7}$, *J* = 8.0 Hz, 2H, H8), 4.53 (t, $J_{4/5\&4/3}$ = 3.0 Hz, 1H, H4), 4.36 – 4.03 (m, 3H, H3, H5), 3.55 – 3.40 (m, 2H, H11), 2.81 (dd, $J_{2a/2b}$ = 18.2 Hz, $J_{2a/3}$ = 7.0 Hz, 1H, H2a), 2.67 – 2.50 (m, 1H, H2b), 2.46 (s, 3H, H10), 1.20 (t, $J_{12/11}$ = 7.0 Hz, 3H, H12) ppm. **^{13}C NMR** (75 MHz, $CDCl_3$) δ 174.3 (C1), 145.8 (C9), 132.0 (C6), 130.3 (C8), 128.1 (C7), 81.9 (C4), 75.5 (C3), 68.4 (C5), 65.2 (C11), 35.4 (C2), 21.9 (C10), 15.2 (C12) ppm. **HRMS** $[M+H]^+$ predicted 315.0897, found 315.0901.

2.3.2. Tosylation of HBO-OH and acetylation of secondary alcohol

HBO-OH was solubilized in pyridine (1 mol.L^{-1}) before the addition of tosyl chloride (1.25 equiv.) and the reaction mixture was stirred for 2 h at room temperature. Acetic anhydride (4 equiv.) was directly added and the mixture stirred at room temperature for 2 h. The reaction was further quenched using 1 M HCl solution and the product was extracted twice using EtOAc. Organic layers were combined, dried over anhydrous $MgSO_4$ and filtered. After concentration, the desired product (**4**) (2.65 g, 77 % raw yield) can either directly be used in the next step or be purified over silica gel (Eluent: Cyclohexane / EtOAc: 50 / 50) to give a purified **HBO-TS-OAc** as clear yellow crystals (51% yield).

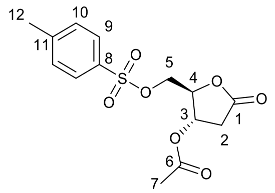

(*S*)-β-acetoxy-(*R*)-γ-tosyloxymethyl-γ-butyrolactone 4d, white solid (11.3 g, 77% yield)
/mp. = 75.5 °C. **$[\alpha]_D^{26.3}$** = +17 (c 0.01, CH_2Cl_2). **^{1}H NMR** (300 MHz, $CDCl_3$) δ 7.75 (d, *J* = 8.4 Hz, 2H, H7), 7.36 (d, 2H, H8), 5.25 (dt, $J_{3/2a}$ = 7.5 Hz, $J_{3/4\&3/2b}$ = 1.7 Hz, 1H, H3), 4.57 (td, $J_{4/5}$ = 2.7 Hz, 1H, H4), 4.37 (dd, $J_{5a/5b}$ = 11.2 Hz, 1H, H5a), 4.19 (dd, 1H, H5b), 2.98 (dd, $J_{2a/2b}$ = 18.7 Hz, 1H, H2a), 2.56 (dd, 1H, H2b), 2.45 (s, 3H, C10), 2.08 (s, 3H, C12) ppm. **^{13}C NMR** (75 MHz, $CDCl_3$) δ 173.5 (C1), 170.5 (C11), 145.8 (C9), 131.9 (C6), 130.3 (C8), 128.1 (C7), 81.8 (C4), 71.1 (C3), 68.4 (c5), 34.7 (c2), 21.8 (c10), 20.8 (C12) ppm. **HRMS** $[M+H]^+$ predicted 329.0690, found 329.0699.

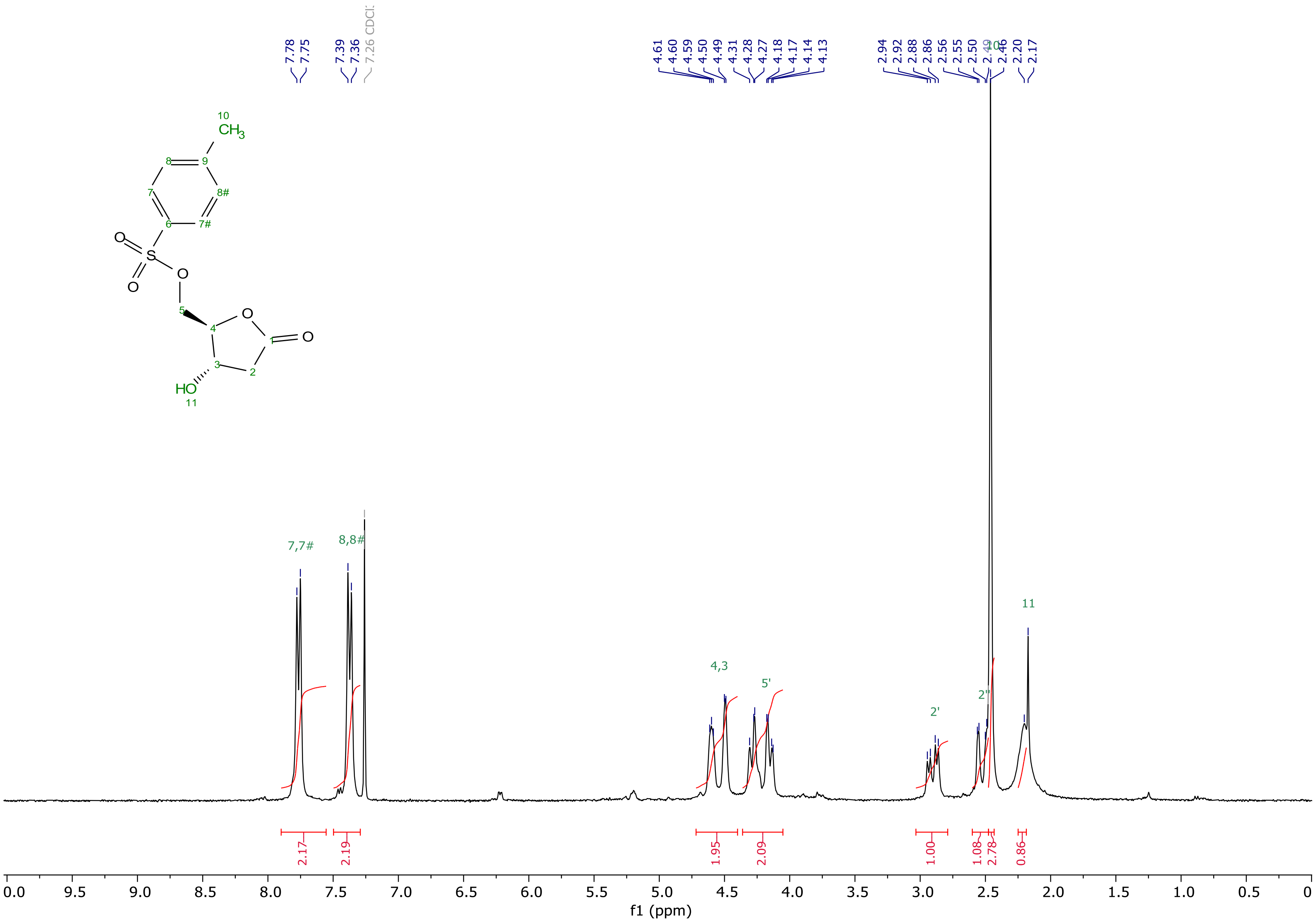

*Figure 13: ^{1}H NMR spectrum of compound **4a***

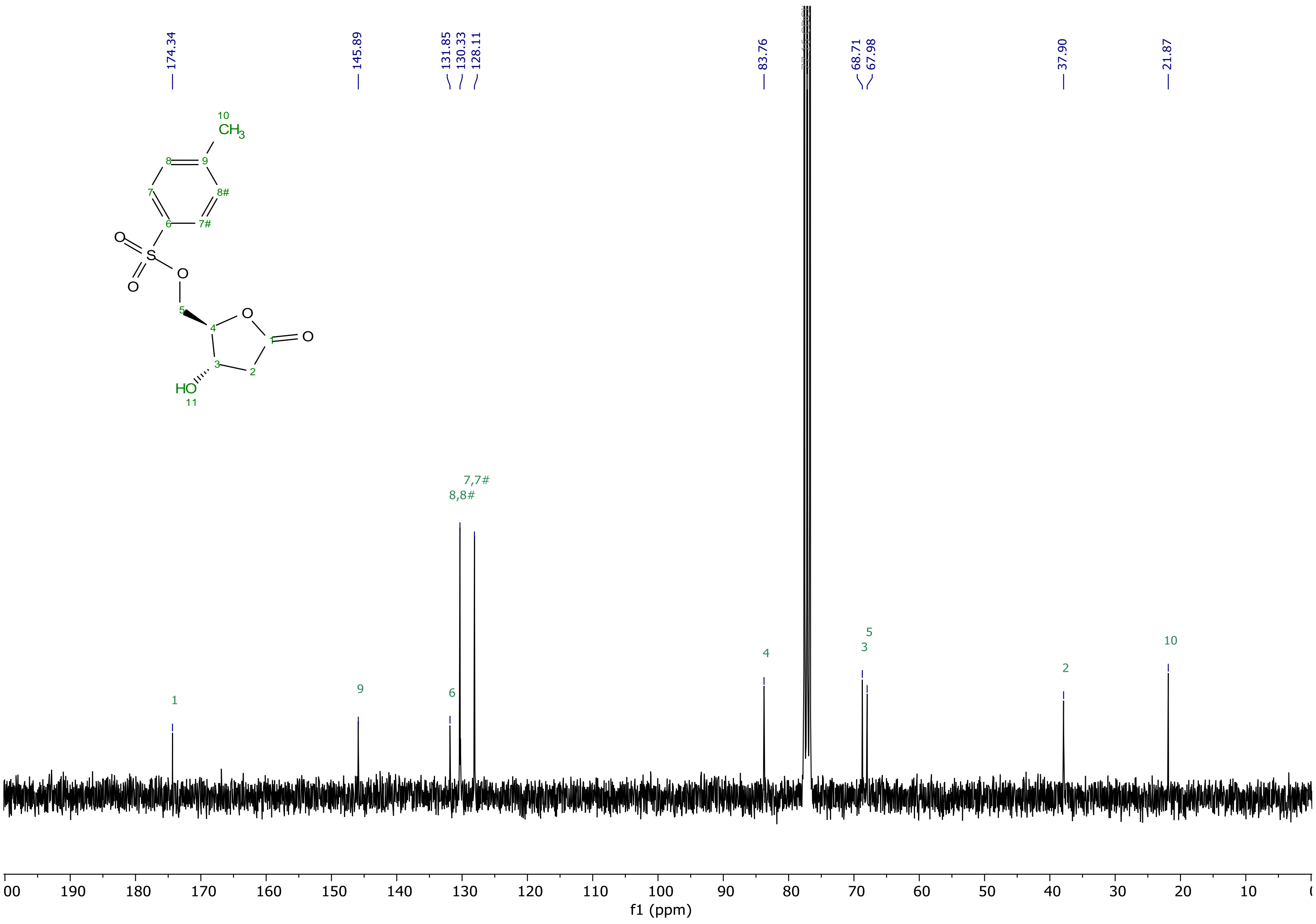

Figure 14: ^{13}C NMR spectrum of compound ***4a***

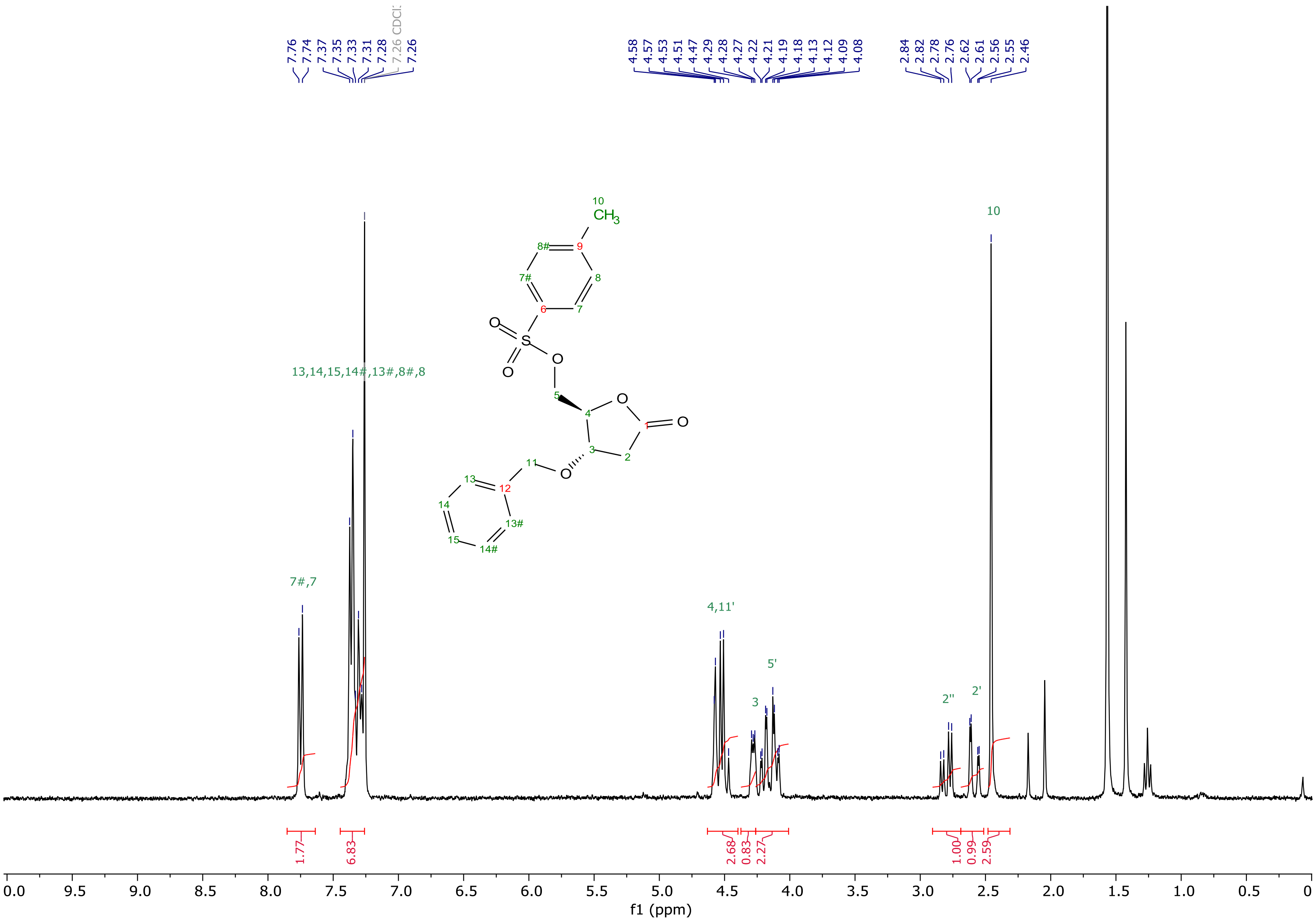

Figure 15: ^{1}H NMR spectrum of compound ***4b***

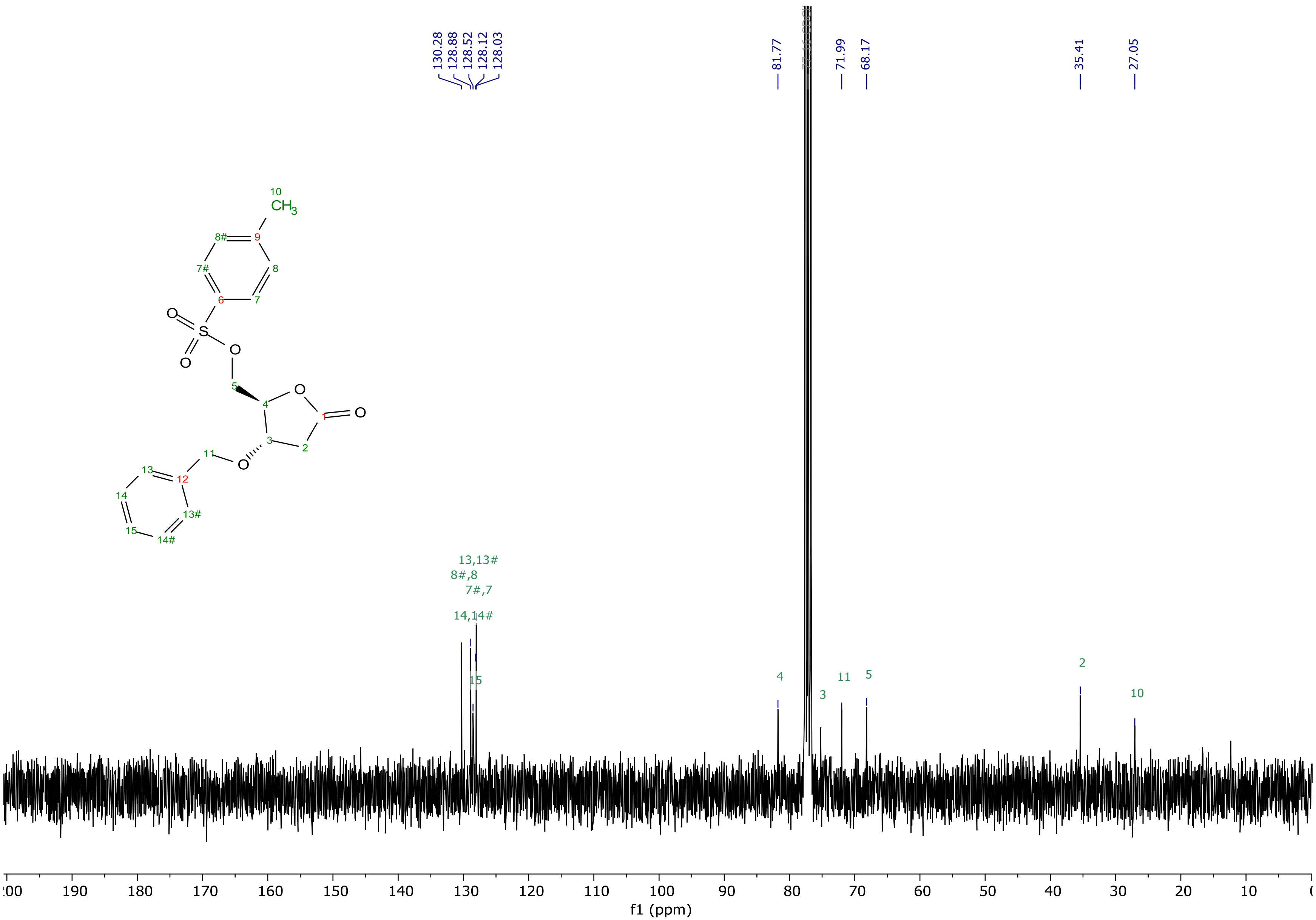

Figure 16: ^{13}C NMR spectrum of compound ***4b***

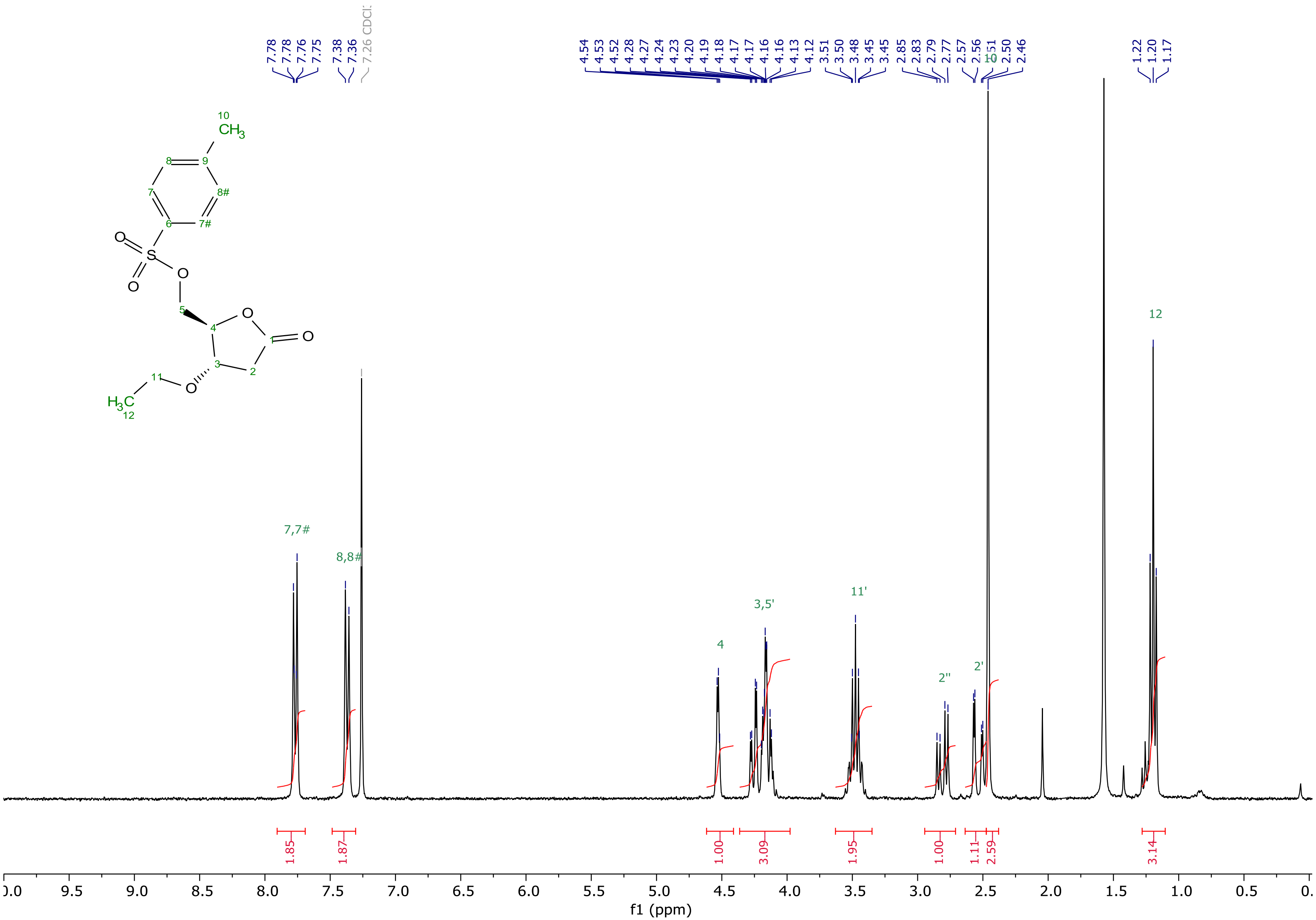

*Figure 17: ^{1}H NMR spectrum of compound **4c***

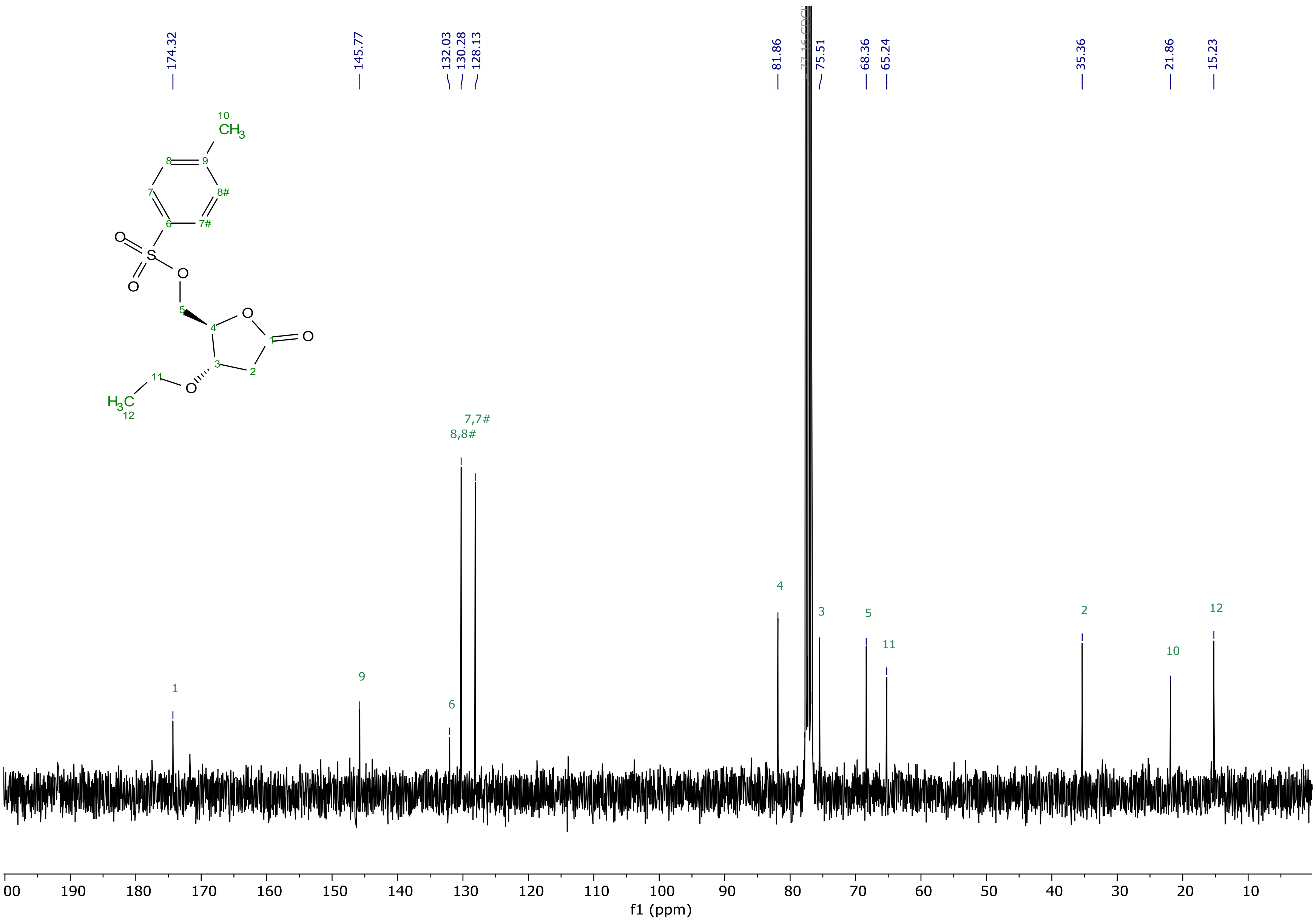

*Figure 18: ^{13}C NMR spectrum of compound **4c***

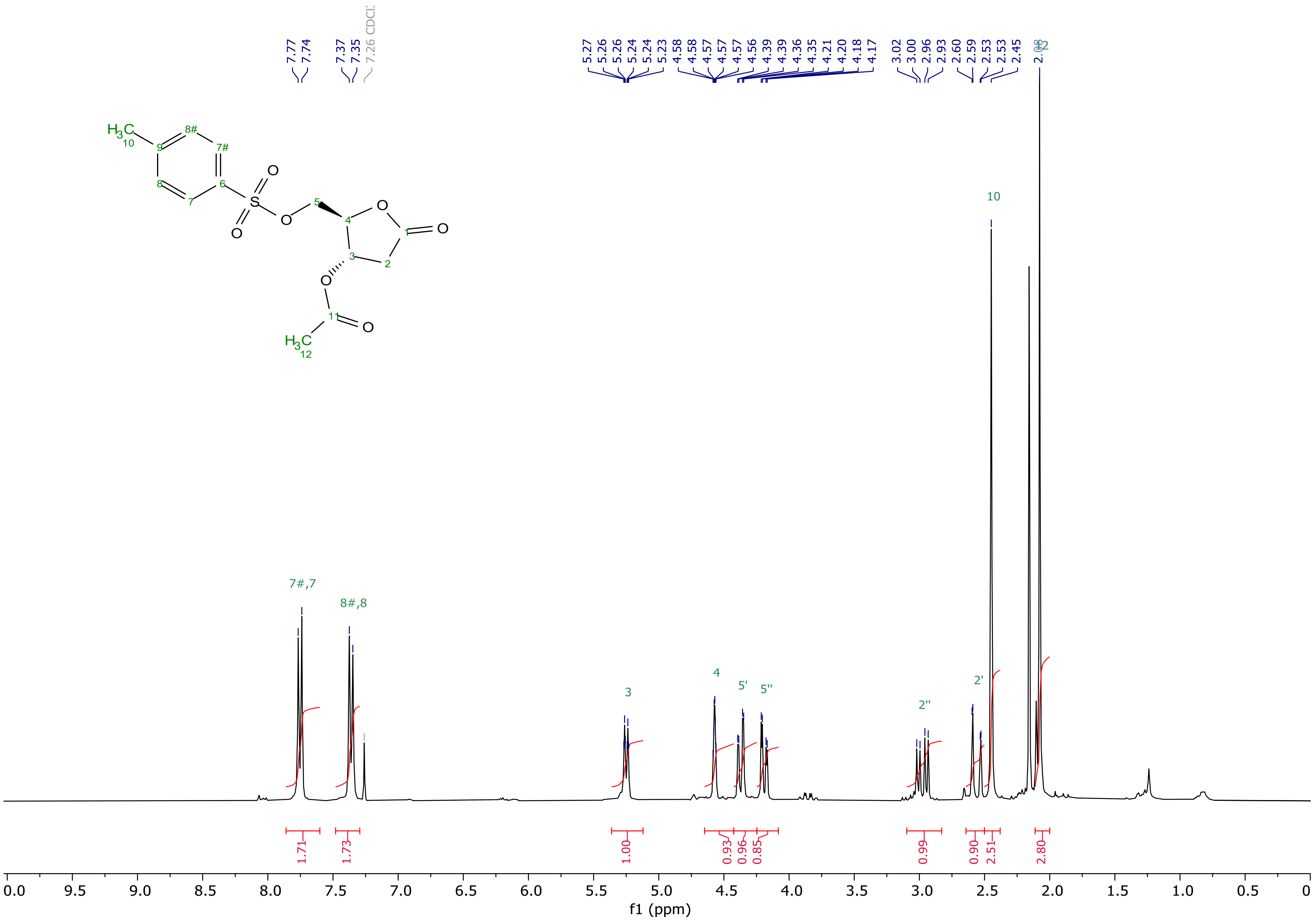

Figure 19: ^{1}H NMR spectrum of compound **4d**

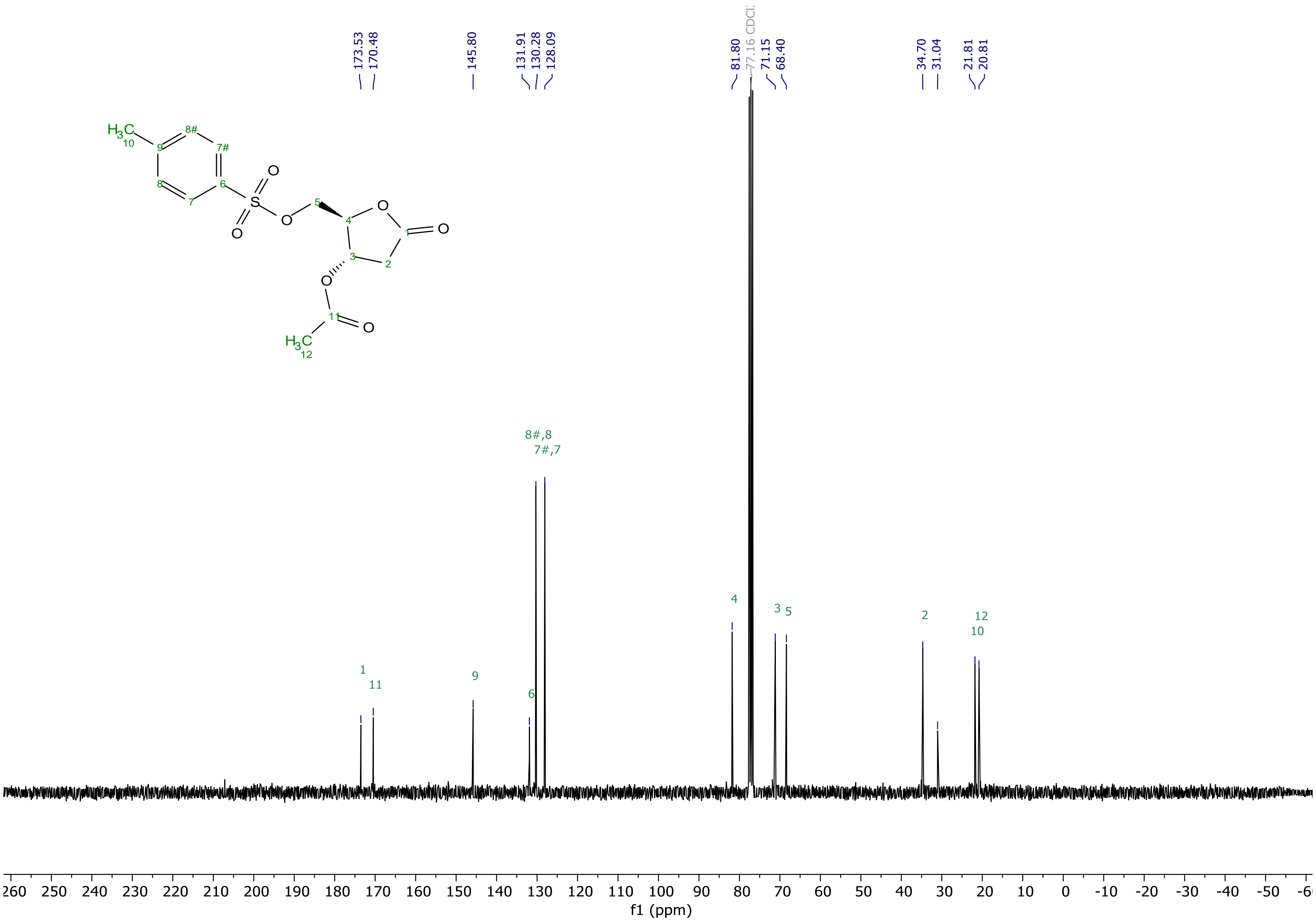

*Figure 20: ^{13}C NMR spectrum of compound **4d***

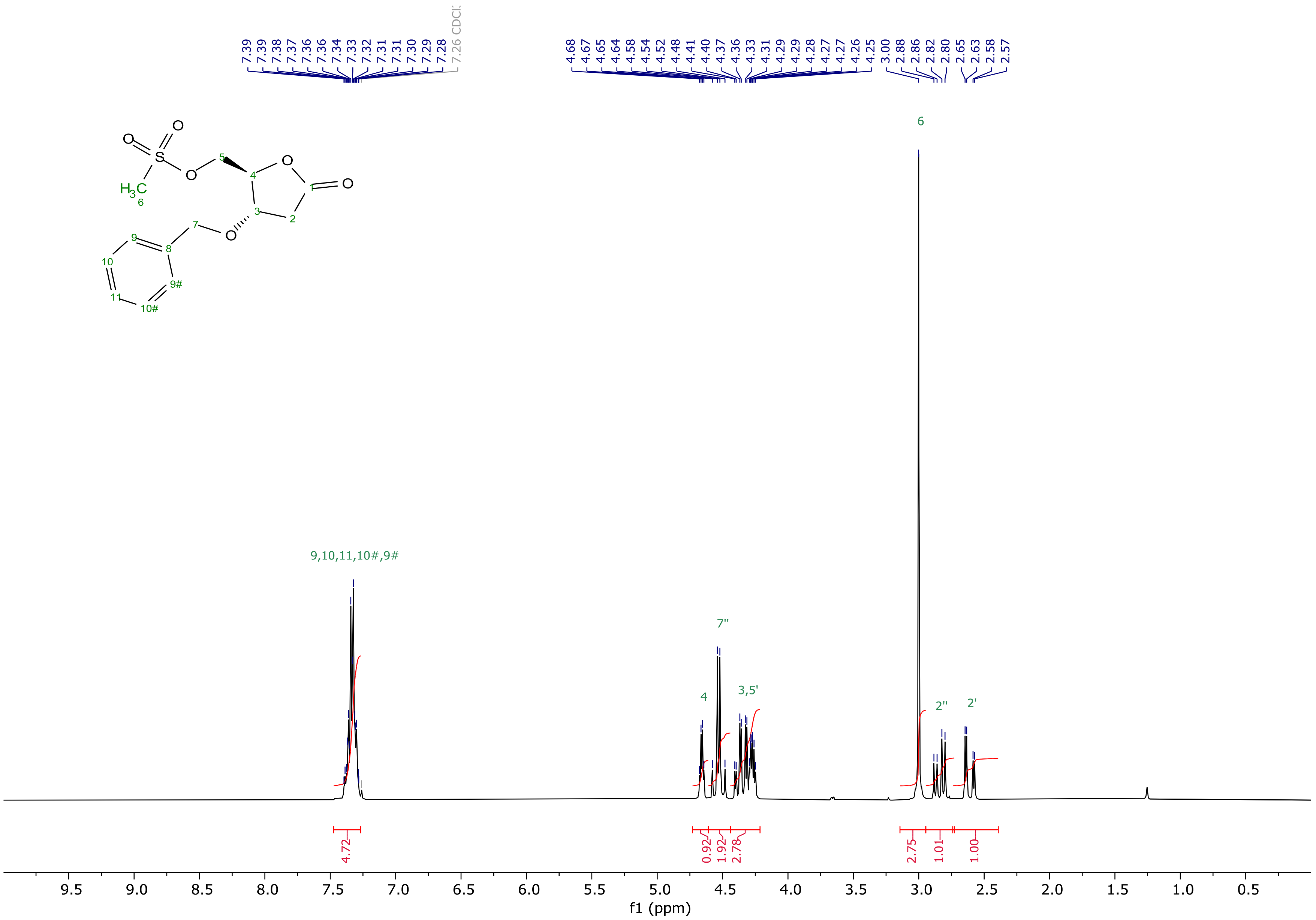

*Figure 21: ^{1}H NMR spectrum of compound **4e***

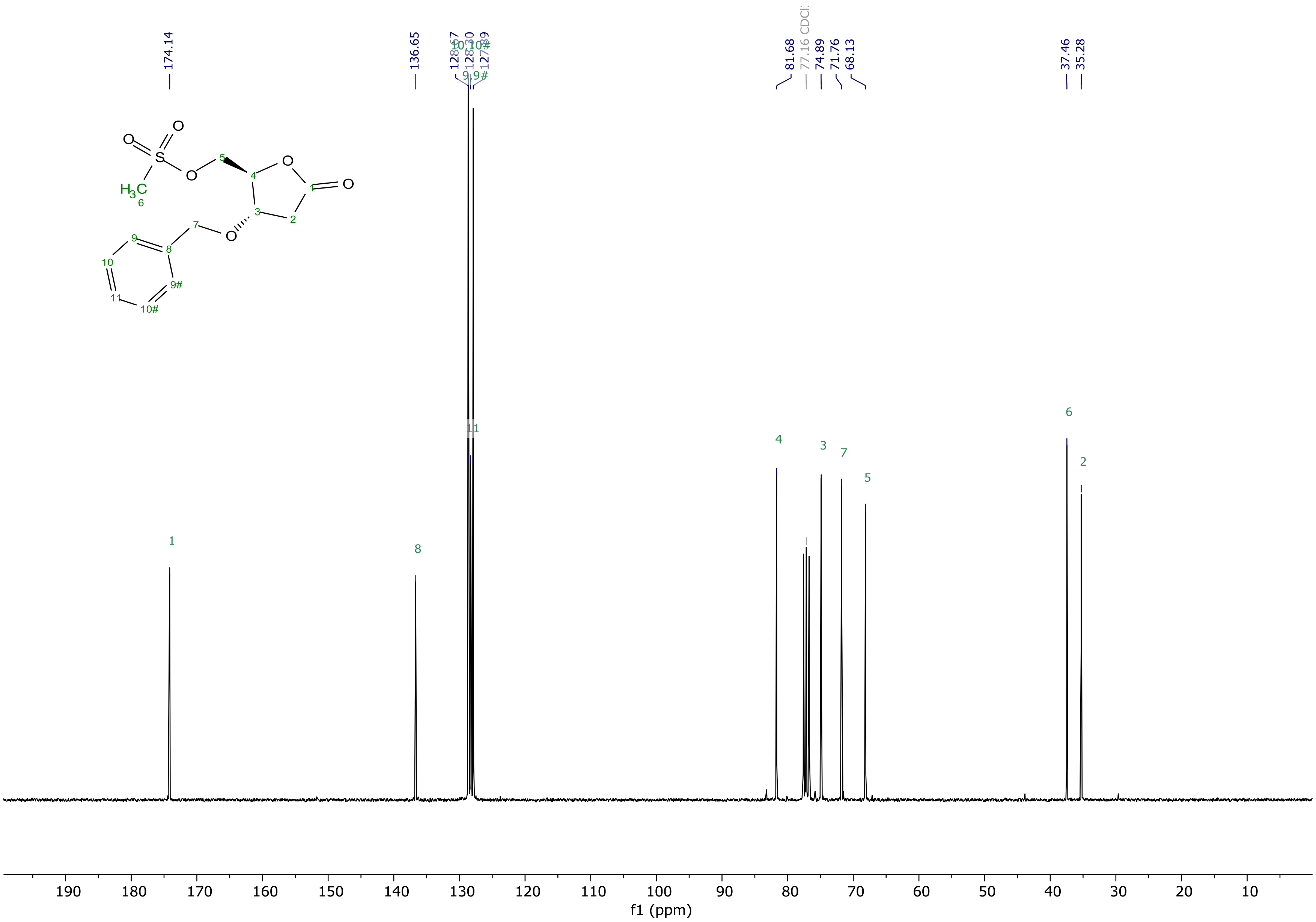

*Figure 22: ^{13}C NMR spectrum of compound **4e***

2.4. General procedure for iodination

Tosylated (or mesylated) derivative was dissolved in acetone (C = 1.0 M) before the addition of sodium iodide (1.25 equiv.) and the reaction was refluxed for 16 h. The reaction mixture is then diluted with EtOAc and washed with a 2.5 M solution of $Na_2S_2O_3$ then with brine. Organic layer was dried over anhydrous $MgSO_4$ and filtered. After concentration, the desired product can directly be engaged in the next step without further purification.

(*S*)-β-hydroxy-(*S*)-γ-iodomethyl-γ-butyrolactone, colourless oil (601 mg, 82% yield)

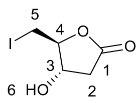

$[\alpha]_D^{21.9}$= -0.16 (c 0.06, AcOEt). **^{1}H NMR** (300 MHz, $CDCl_3$) δ 4.57 – 4.37 (m, 1H, H3, H4), 3.41 (dd, $J_{5a/5b}$ = 10.8 Hz, $J_{5a/4}$ = 4.2 Hz, 1H, H5a), 3.28 (dd, $J_{5b/4}$ = 7.3 Hz, 1H, H5b), 2.98 (dd, $J_{2a/2b}$ = 18.3 Hz, $J_{2a/3}$ = 7.3 Hz, 1H, H2a), 2.59 (dd, $J_{2b/3}$ = 4.2 Hz, 2H, H2b, H6) ppm. **^{13}C NMR** (75 MHz, $CDCl_3$): δ = 174.0 (C1), 85.3 (C4), 72.0 (C3), 37.9 (C2), 3.9 (C5) ppm. **HRMS** $[M+Na]^+$ predicted 264.9332, found 264.9334.

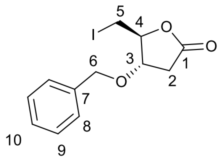

***(S)*-β-benzyloxy-(S)-γ-iodomethyl-γ-butyrolactone,** colourless oil (140 mg, 88 %)

$[\alpha]_D^{21.3}$ = - 1.18 (c 0.051, EtOAc). **^{1}H NMR** (300 MHz, $CDCl_3$) δ 7.42 – 7.29 (m, 5H, C8, C9, C10), 4.58 (s, 2H, C6), 4.56 – 4.49 (m, 1H, C4), 4.18 (dt, $J_{3/2a}$ = 7.3 Hz, $J_{3/2b\&4}$ = 3.0 Hz, 1H, H3), 3.34 (dd, $J_{5a/5b}$ = 10.9 Hz, $J_{5a/4}$ = 4.1 Hz, 1H, H5a), 3.23 (dd, $J_{5b/4}$ = 7.1 Hz, 1H, H5b), 2.88 (dd, $J_{2a/2b}$ = 18.3 Hz, 1H, H2a), 2.62 (dd, 1H, H2b) ppm. **^{13}C NMR** (75 MHz, $CDCl_3$): δ = 174.0 (C1), 136.8 (C7), 128.8 (C9), 128.5 (C10), 128.0 (C8), 83.3 (C4), 78.1 (C3), 71.8 (C6), 35.6 (C2), 4.4 (C5) ppm. **HRMS** $[M+Na]^+$ predicted 354.9801, found 354.9804.

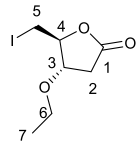

(*S*)-β-ethoxy-(*S*)-γ-iodomethyl-γ-butyrolactone, colourless oil (213 mg, 80% yield)

$[\alpha]_D^{26.3}$ = -18.8 (c 0.026, EtOAc). **^{1}H NMR** (300 MHz, $CDCl_3$) δ 4.47 (ddd, $J_{4/5b}$ = 6.9 Hz, $J_{4/5a}$ = 4.2 Hz, $J_{4/3}$ = 2.6 Hz, 1H, H4), 4.08 (ddd, $J_{3/2a}$ = 7.2 Hz, $J_{3/2b}$ = 3.3 Hz, 1H, H3), 3.54 (qd, $J_{6/7}$ = 7.0 Hz, $J_{6/3}$ = 2.5 Hz, 2H, H6), 3.39 (dd, $J_{5a/5b}$ = 10.9 Hz, 1H, H5a), 3.27 (dd, 1H, H5b), 2.90 (dd, $J_{2a/2b}$ = 18.3 Hz, 1H, H2a), 2.57 (dd, 1H, H2b), 1.22 (t, J = 7.0 Hz, 3H, H7) ppm. **^{13}C NMR** (75 MHz, $CDCl_3$): δ = 174.3 (C1), 83.2 (C4), 78.4 (C3), 65.2 (C6), 35.6 (C2), 15.3 (C7), 4.7 (C5) ppm. **HRMS** $[M+Na]^+$ predicted 292.9645, found 292.9645.

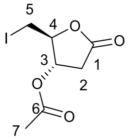

(*S*)-β-acetoxy-(*S*)-γ-iodomethyl-γ-butyrolactone, colourless oil (566 mg, 91% yield)

$[\alpha]_D^{26.3}$ = -7 (c 0.06, acetone). **^{1}H NMR** (300 MHz, $CDCl_3$) δ 5.16 (dt, $J_{3/2a}$ = 8.0 Hz, $J_{3/2b\&3/4}$ = 2.4 Hz, 1H, H3), 4.51 (ddd, $J_{4/5b}$ = 5.8 Hz, $J_{4/5a}$ = 3.9 Hz, 1H, H4), 3.57 – 3.36 (m, 2H, H5), 3.27 – 2.95 (m, 1H, H2a), 2.60 (dd, $J_{2b/2a}$ = 19.0 Hz, 1H, H2b), 2.11 (s, 3H, H7) ppm. **^{13}C NMR** (75 MHz, $CDCl_3$) δ 173.3 (C1), 170.5 (C6), 82.9 (C4), 73.9 (C3), 35.2 (C2), 20.9 (C7), 4.8 (C5) ppm. **HRMS** $[M+Na]^+$ predicted 306.9438, found 306.9437.

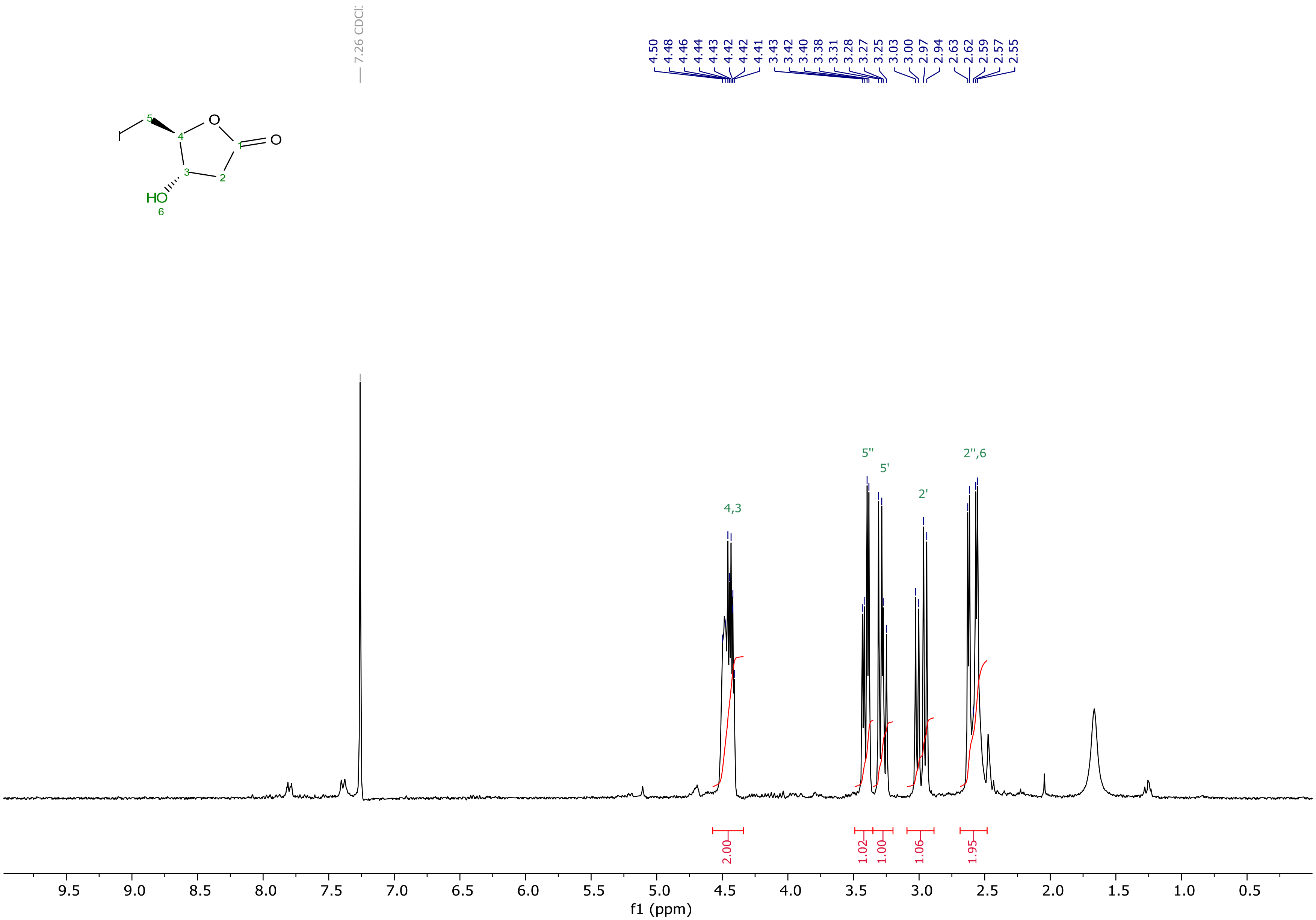

Figure 23: ^{1}H NMR spectrum of compound ***(S)-β-hydroxy-(S)-γ-iodomethyl-γ-butyrolactone***

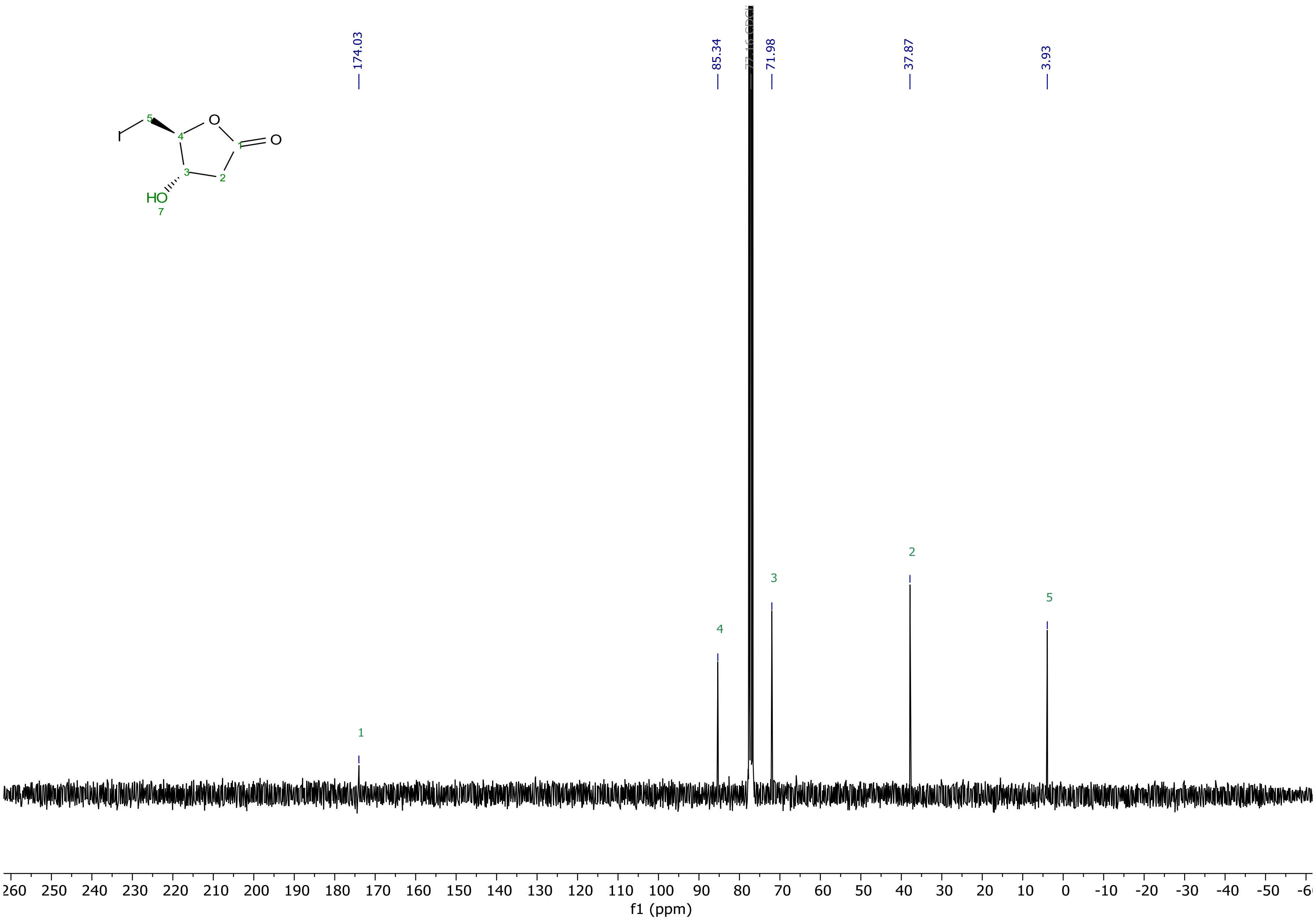

Figure 24: ^{13}C NMR spectrum of compound ***(S)-β-hydroxy-(S)-γ-iodomethyl-γ-butyrolactone***

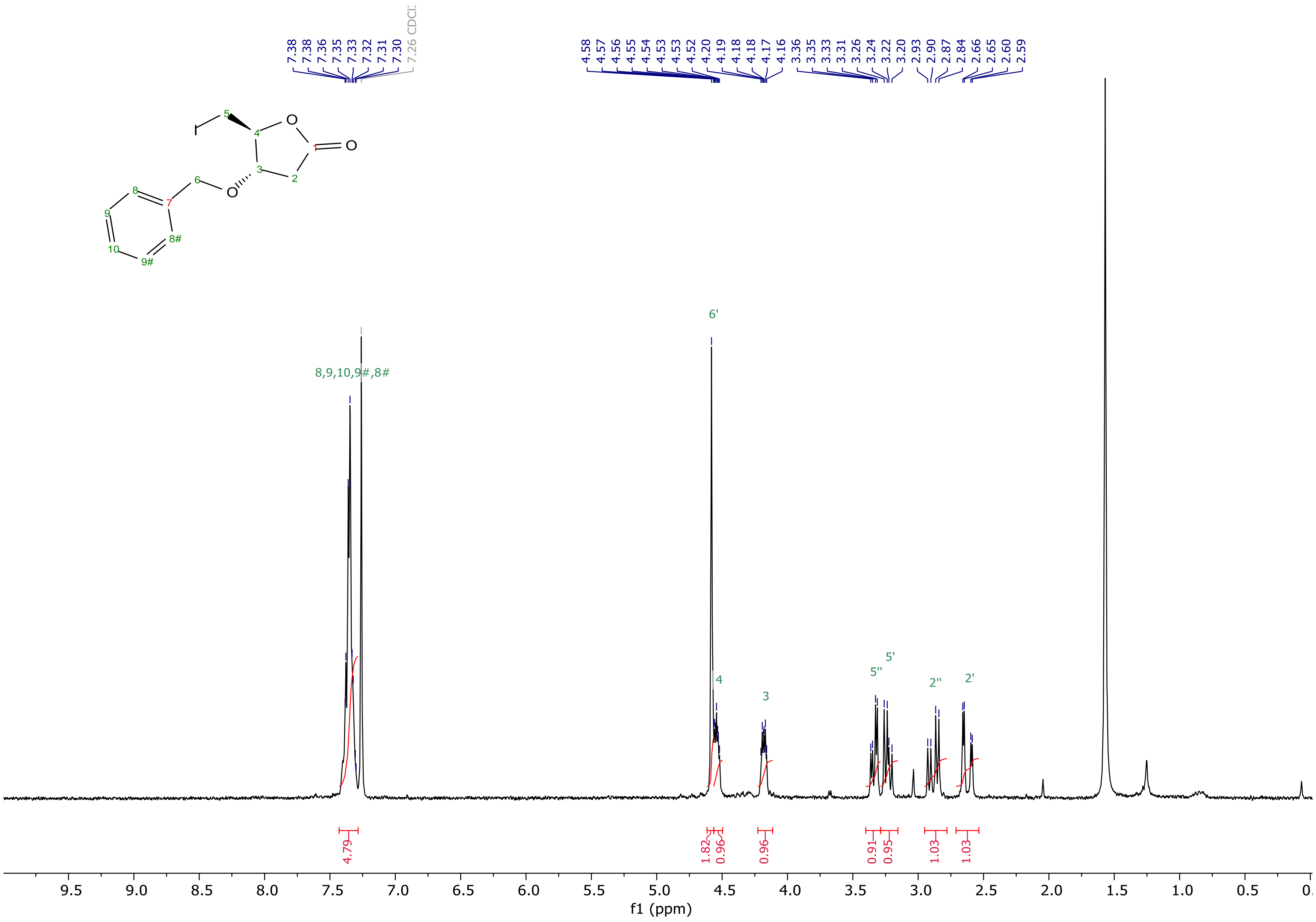

Figure 25: ^{1}H NMR spectrum of compound ***(S)-β-benzyloxy-(S)-γ-iodomethyl-γ-butyrolactone***

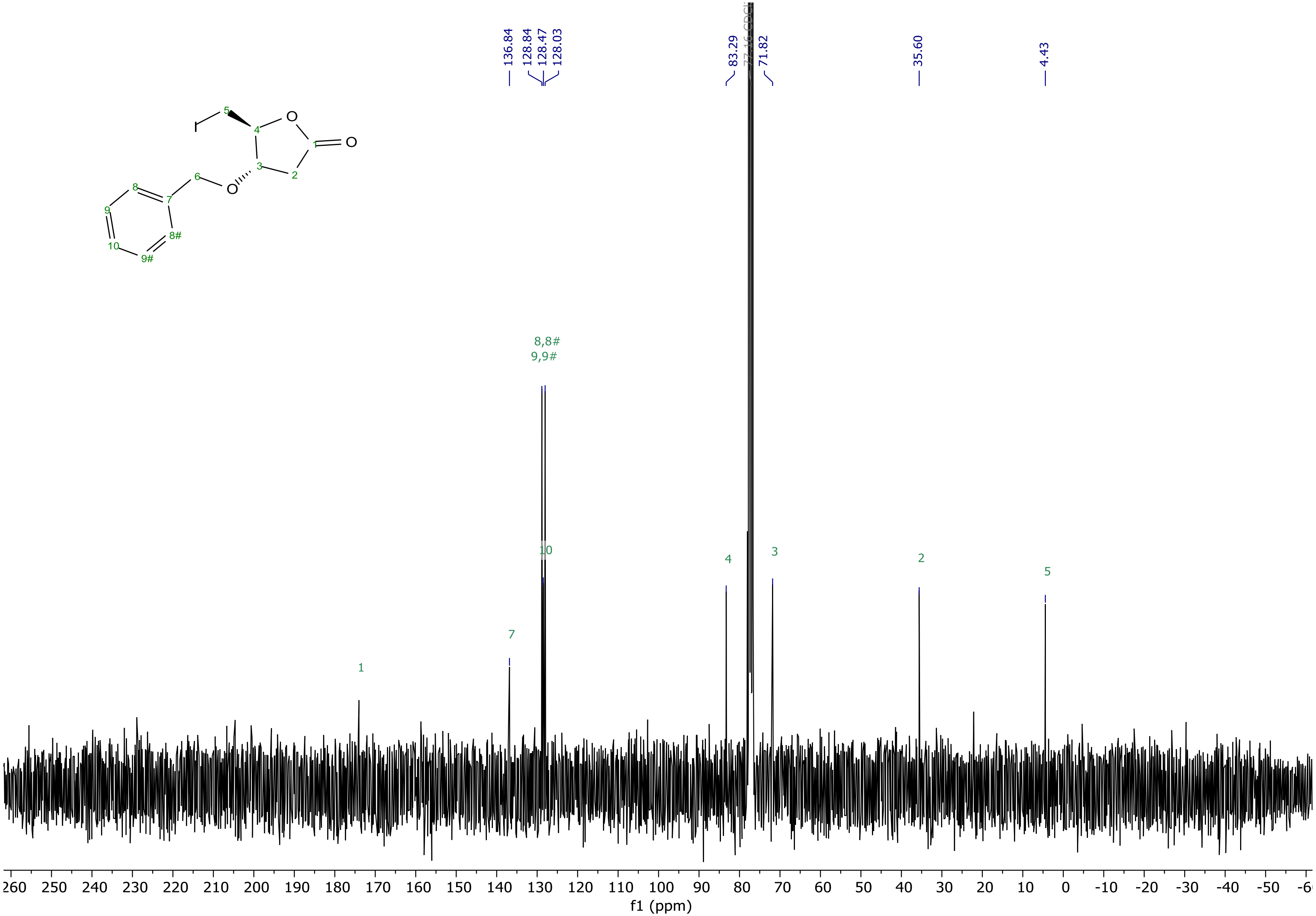

Figure 26: ^{13}C NMR spectrum of compound **(S)-*β-benzyloxy-(S)-γ-iodomethyl-γ-butyrolactone***

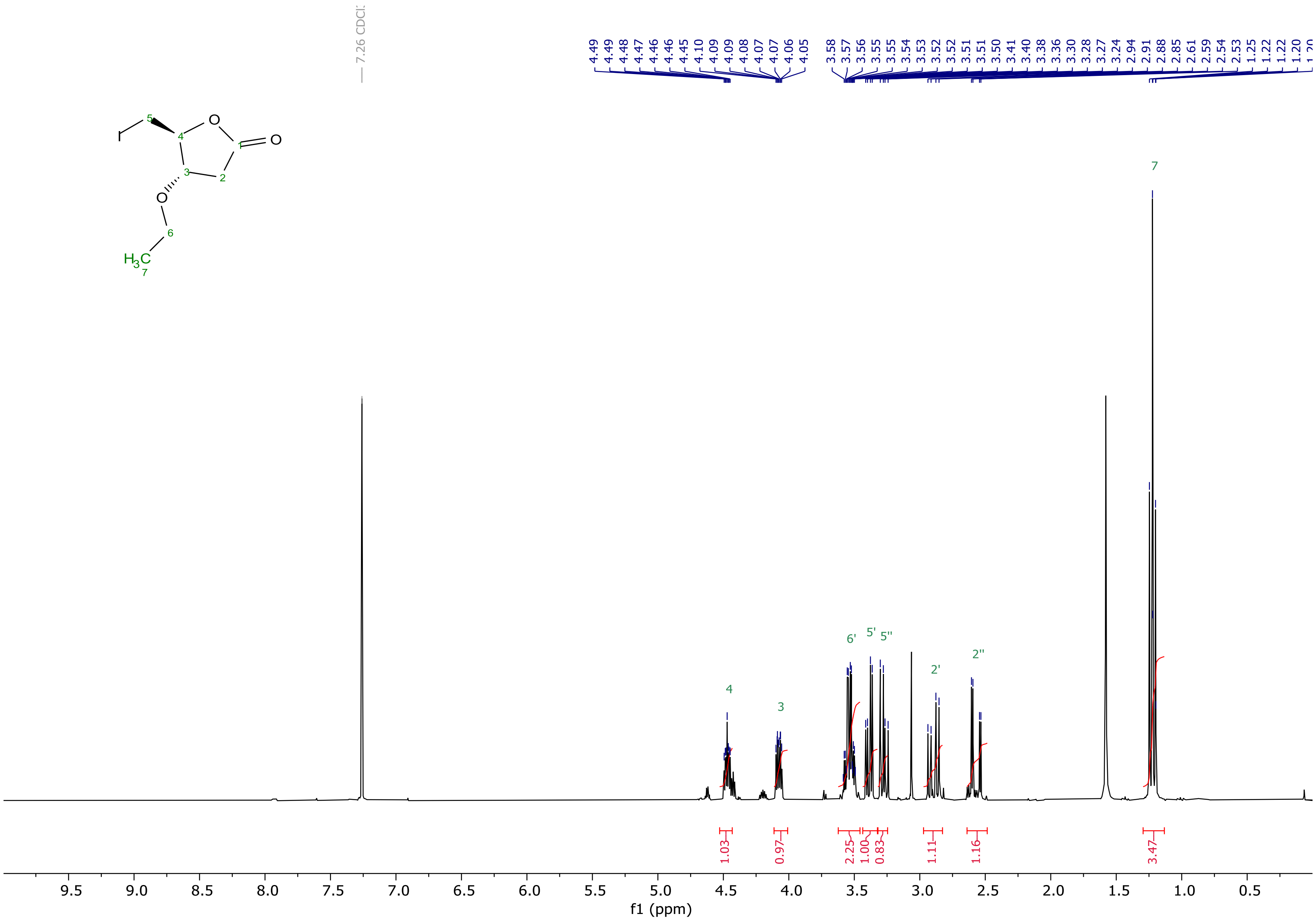

Figure 27: ^{1}H NMR spectrum of compound ***(S)-β-ethoxy-(S)-γ-iodomethyl-γ-butyrolactone***

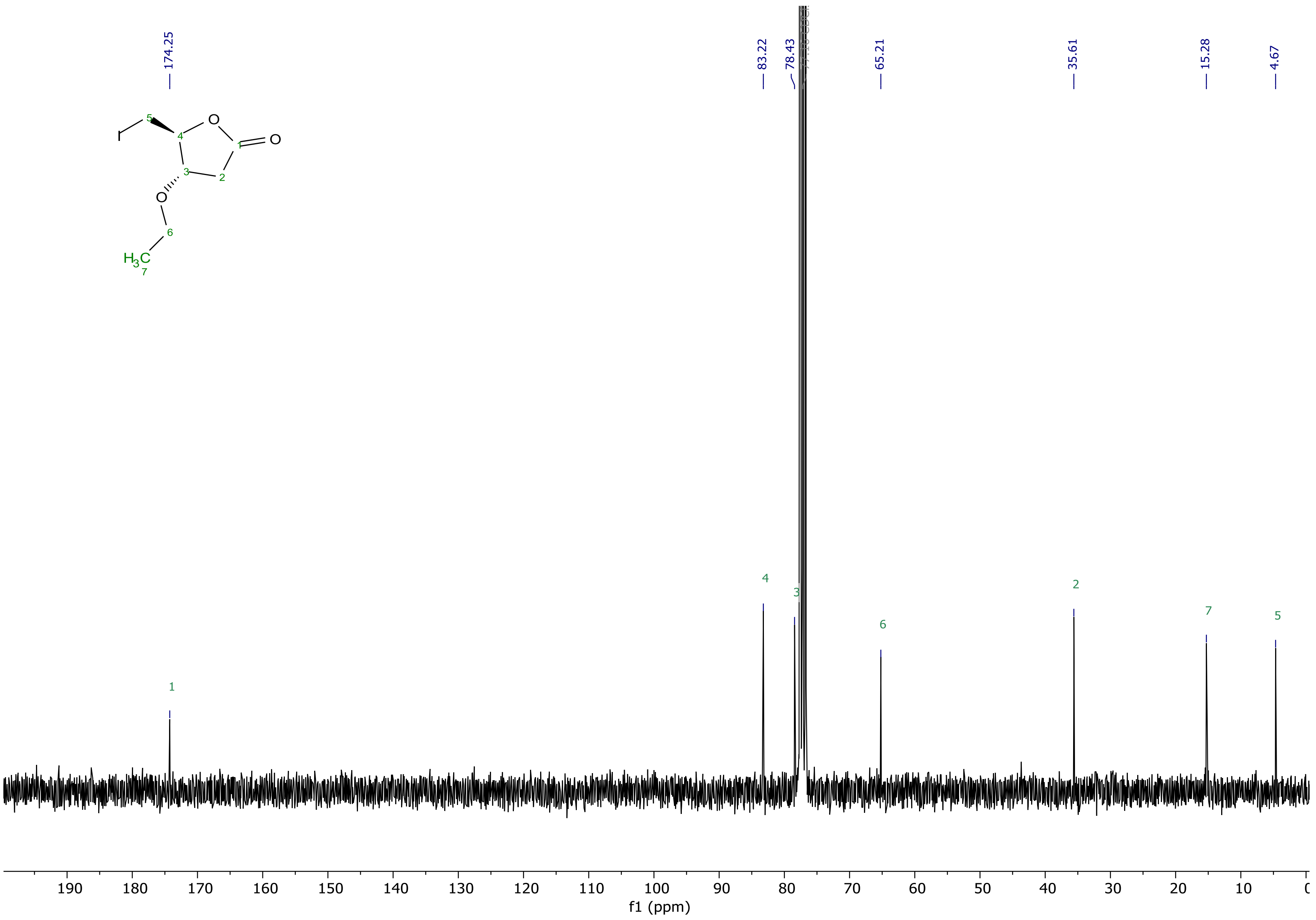

Figure 28: ^{13}C NMR spectrum of compound ***(S)-β-ethoxy-(S)-γ-iodomethyl-γ-butyrolactone***

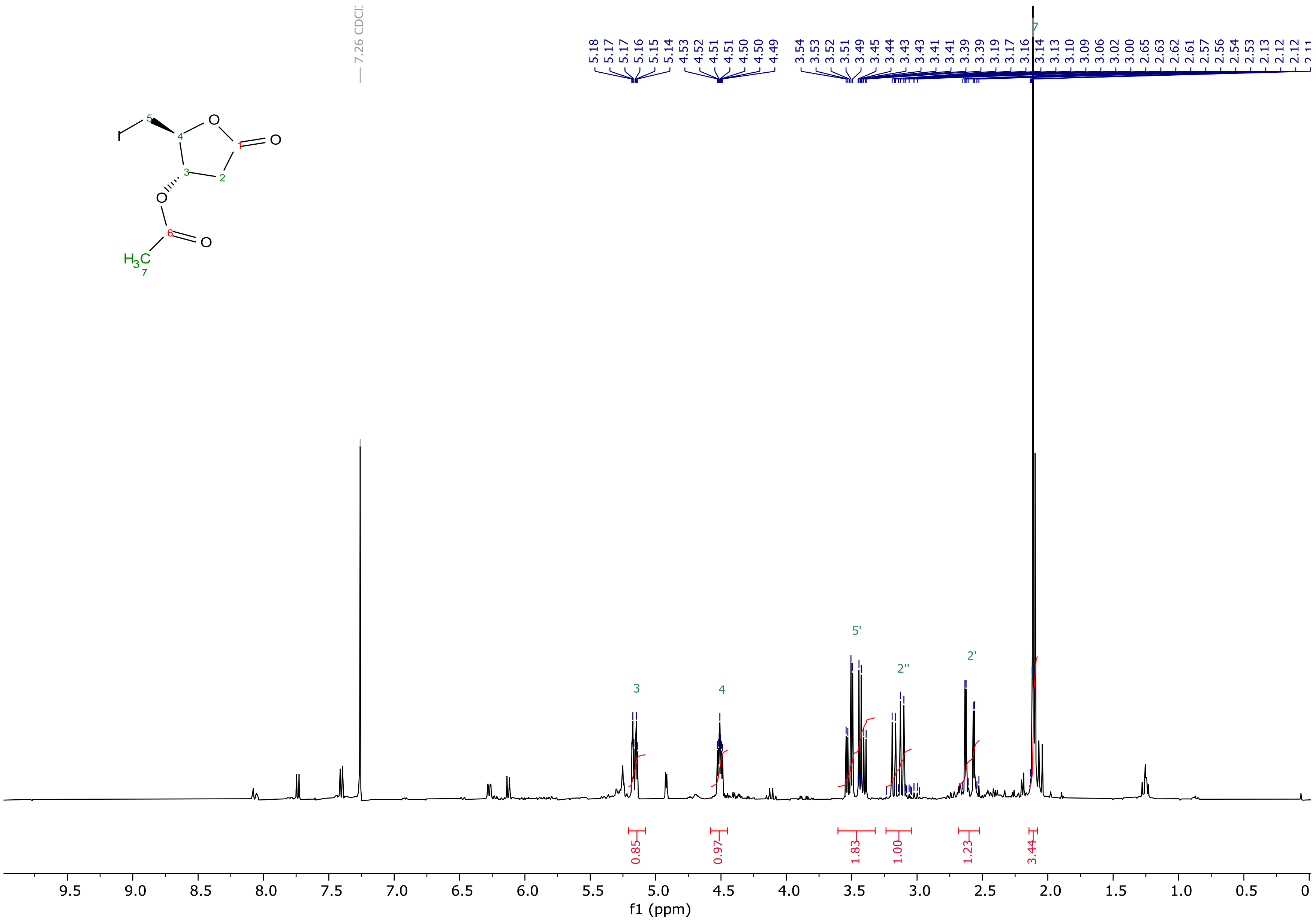

Figure 29: ^{1}H NMR spectrum of compound ***(S)-β-acetoxy-(S)-γ-iodomethyl-γ-butyrolactone***

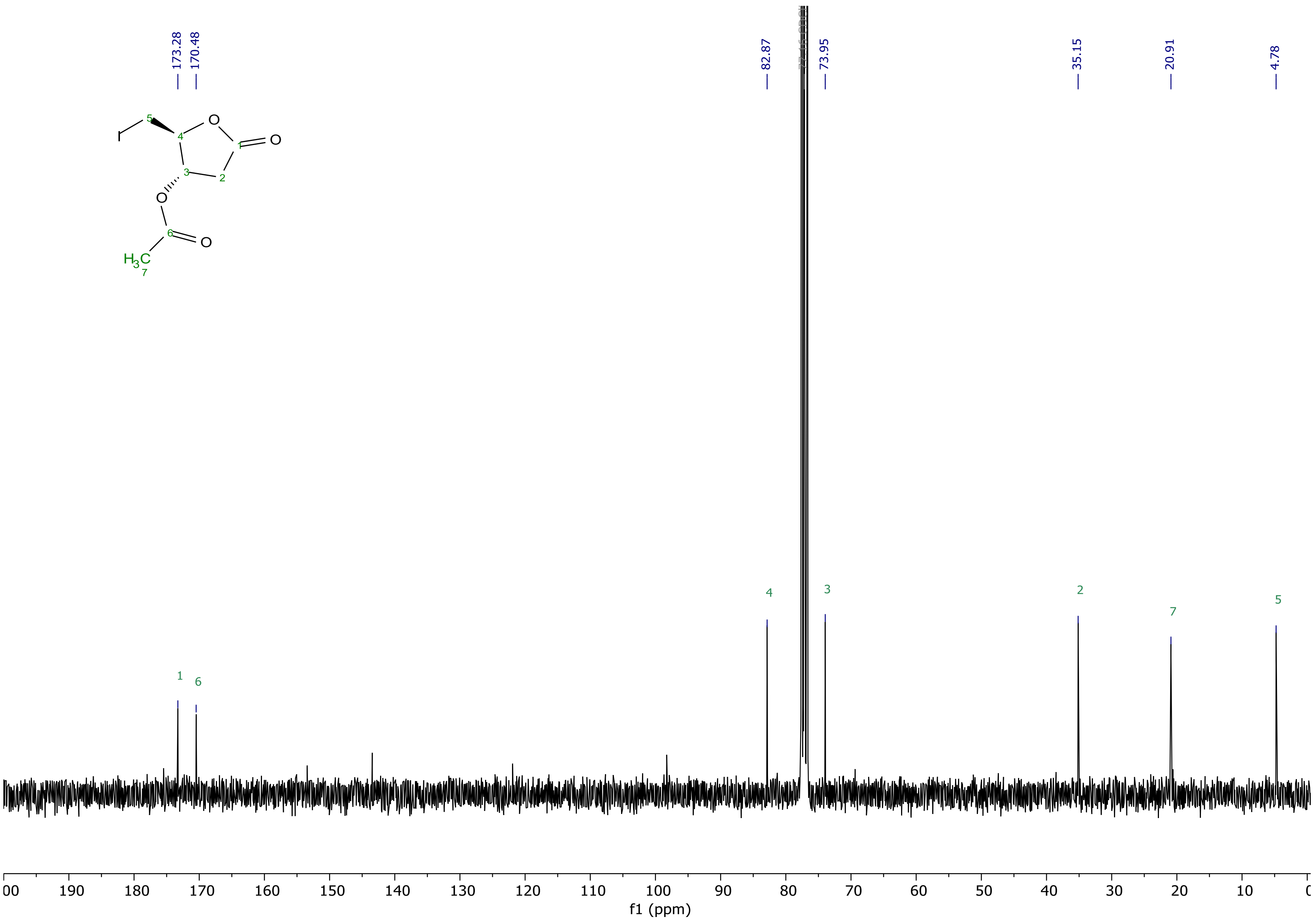

Figure 30: ^{13}C NMR spectrum of compound ***(S)-β-acetoxy-(S)-γ-iodomethyl-γ-butyrolactone***

2.5. General procedure for Bernet-Vasella reaction

Zinc dust (1 g) was activated in 1 M HCl (15 mL), filtered, rinsed with water, ethanol and diethyl ether.

Iodomethyl-compound was dissolved in a mixture of cyclopentyl methyl ether (CPME) or acetone and water (4:1, C = 1 M) before the addition of activated zinc dust (1.5 equiv.) and the reaction was stirred at 60°C for 1 h. The reaction mixture was filtered over a Celite pad, rinsed by EtOAc and water. The aqueous layer was acidified to pH = 1 and the product was extracted thrice with EtOAc. Organic layers were combined and dried over anhydrous $MgSO_4$, filtered and concentrated.

In the case of the more hydrophilic compound **5a**, addition of NaCl in the aqueous layer allows the salting out of the desired product, and thus results in a better recovery.

2.6. One-pot procedure for iodination and Bernet-Vasella reaction.

Tosylated (or mesylated) derivative was dissolved in acetone (C = 1.0 M) before the addition of sodium iodide (1.25 equiv.) and the reaction was heated at 60°C for 16 h. Then, activated zinc dust (1.5 equiv.) and water (20% of reaction volume) were added and the reaction was stirred at 60°C for 1.5 h. The reaction mixture was filtered over a Celite pad, rinsed by EtOAc and water. The aqueous layer was acidified to pH = 1 and the product was extracted thrice with EtOAc. Organic layers were combined and dried over anhydrous $MgSO_4$, filtered and concentrated.

(*S*)-3-hydroxypent-4-enoic acid 5a, yellow oil (667 mg, 91% yield)

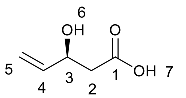

$[\alpha]_D^{21.9}$ = +12.8 (c 0.046, EtOAc). **^{1}H NMR** (300 MHz, DMSO) δ 5.86 (ddd, $J_{4/5a}$ = 16.4 Hz, $J_{4/5b}$ = 10.4, $J_{4/3}$ = 5.2 Hz, 1H, H4), 5.24 – 5.13 (m, 1H, H5a), 5.01 (d, 1H, H5b), 4.39-4.27 (m, 1H, H3), 2.46 – 2.17 (m, 2H, H2) ppm. **^{13}C NMR** ($(CD_3)_2SO$, 75 MHz): δ = 172.5 (C1), 141.4 (C4), 113.7 (C5), 68.2 (C3), 42.5 (C2) ppm. **HRMS** $[M-H]^-$ predicted 115.0400, found 115.0395.

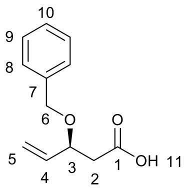

(*S*)-3-(benzyloxy)pent-4-enoic acid 5b, colourless oil (240 mg, 95% yield)

$[\alpha]_D^{22.1}$ = -20.5 (c 0.039, EtOAc) [**$[\alpha]_D^{25}$** = -19.00 (c 1.0, chloroform) lit.[4]]. **^{1}H NMR** (300 MHz, $CDCl_3$) δ 7.45 – 7.17 (m, 5H, H8, H9, H10), 5.77 (ddd, $J_{4/5a}$ = 17.6 Hz, $J_{4/5b}$ = 10.2 Hz, $J_{4/3}$ = 7.6 Hz, 1H, H4), 5.37 – 5.22 (m, 2H, H5), 4.58 (d, $J_{6a/6b}$ = 11.7 Hz, 1H, H6a), 4.38 (d, 1H, H6b), 4.30 – 4.17 (m, 1H, H3), 2.70 (dd, $J_{2a/2b}$ = 15.4 Hz, $J_{2a/3}$ = 8.2 Hz, 1H, H2a), 2.53 (dd, $J_{2b/3}$ = 5.2 Hz, 1H, H2b) ppm. **^{13}C NMR** ((75 MHz, $CDCl_3$) δ 176.8 (C1), 138.0 (C7), 136.8 (C4), 128.4 (9C), 127.9 (C10), 127.8 (C8), 118.7 (C5), 76.7 (C3), 70.6 (C6), 41.0 (C2) ppm. **HRMS** $[M-H]^-$ predicted 205.0870, found 205.0869.

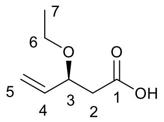

(*S*)-3-ethoxypent-4-enoic acid 5c, pale yellow oil (146 mg, 98% yield)

$[\alpha]_D^{24.5}$ = -3.2 (c 0.040, EtOAc). **^{1}H NMR** (300 MHz, $CDCl_3$) δ 5.73 (ddd, $J_{4/5a}$ = 17.5 Hz, $J_{4/5b}$ = 10.2Hz, $J_{4/3}$ = 7.4 Hz, 1H, 4), 5.41 – 5.18 (m, 2H, H5), 4.25-4.08 (m, 1H, H3), 3.61 (dq, $J_{6a/6b}$ = 9.3 Hz, $J_{6a/7}$= 7.0 Hz, 1H, H6a), 3.42 (dq, $J_{6b/7}$ = 6.9 Hz, 1H, H6b), 2.72 – 2.48 (m, 2H, H2), 1.20 (t, 3H, H7).ppm. **^{13}C NMR** (75 MHz, $CDCl_3$) δ 176.6 (C1), 137.1 (C4), 117.8 (C5), 77.0 (C3), 64.3 (C6), 40.9 (C2), 15.1 (C7) ppm. **HRMS** $[M-H]^-$ predicted 143.0713, found 143.0716.

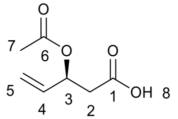

(*S*)-3-acetoxypent-4-enoic acid 5d, yellow oil (380 mg, 98% yield)

$[\alpha]_D^{23.5}$ = -12 (c 0.01, EtOAc). **^{1}H NMR** (300 MHz, DMSO) δ 5.85 (ddd, $J_{4/5a}$ = 17.3 Hz, $J_{4/5b}$ = 10.6 Hz, $J_{4/3}$ = 5.6 Hz, 1H, H4), 5.48 (dtt, $J_{3/2a}$ = 8.3 Hz, $J_{3/2b}$ =1.3 Hz, 1H, H3), 5.31 – 5.08 (m, 2H, H5), 2.77 – 2.42 (m, 2H, H2), 2.00 (s, 3H, H7) ppm. **^{13}C NMR** (75 MHz, DMSO) δ 171.2 (C1), 169.4 (C6), 135.9 (C4), 116.6 (C5), 70.5 (C3), 38.8 (C2), 20.8 (C7) ppm. No ionization was recorded from HRMS analysis.

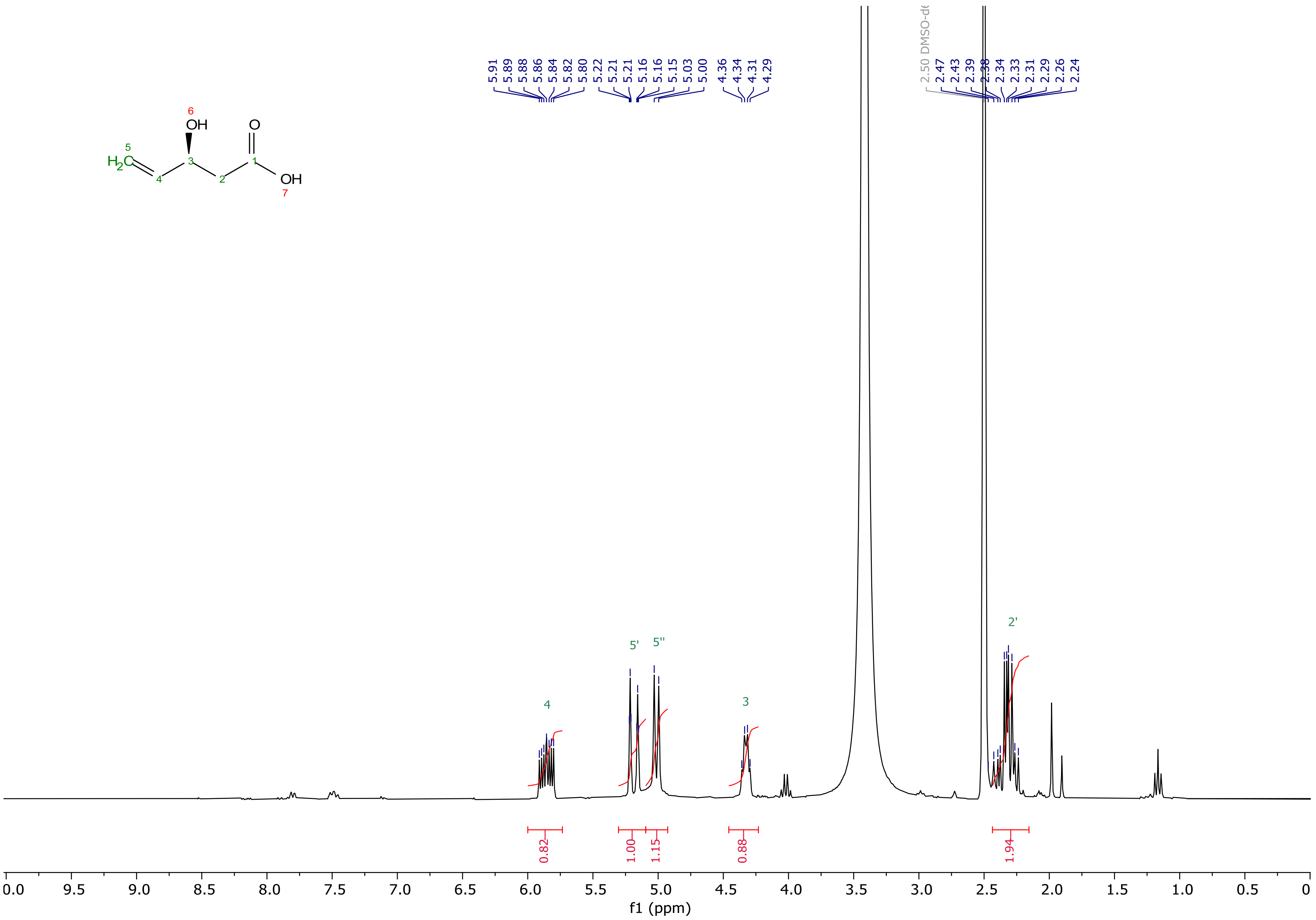

Figure 31: ^{1}H NMR spectrum of compound ***5a***

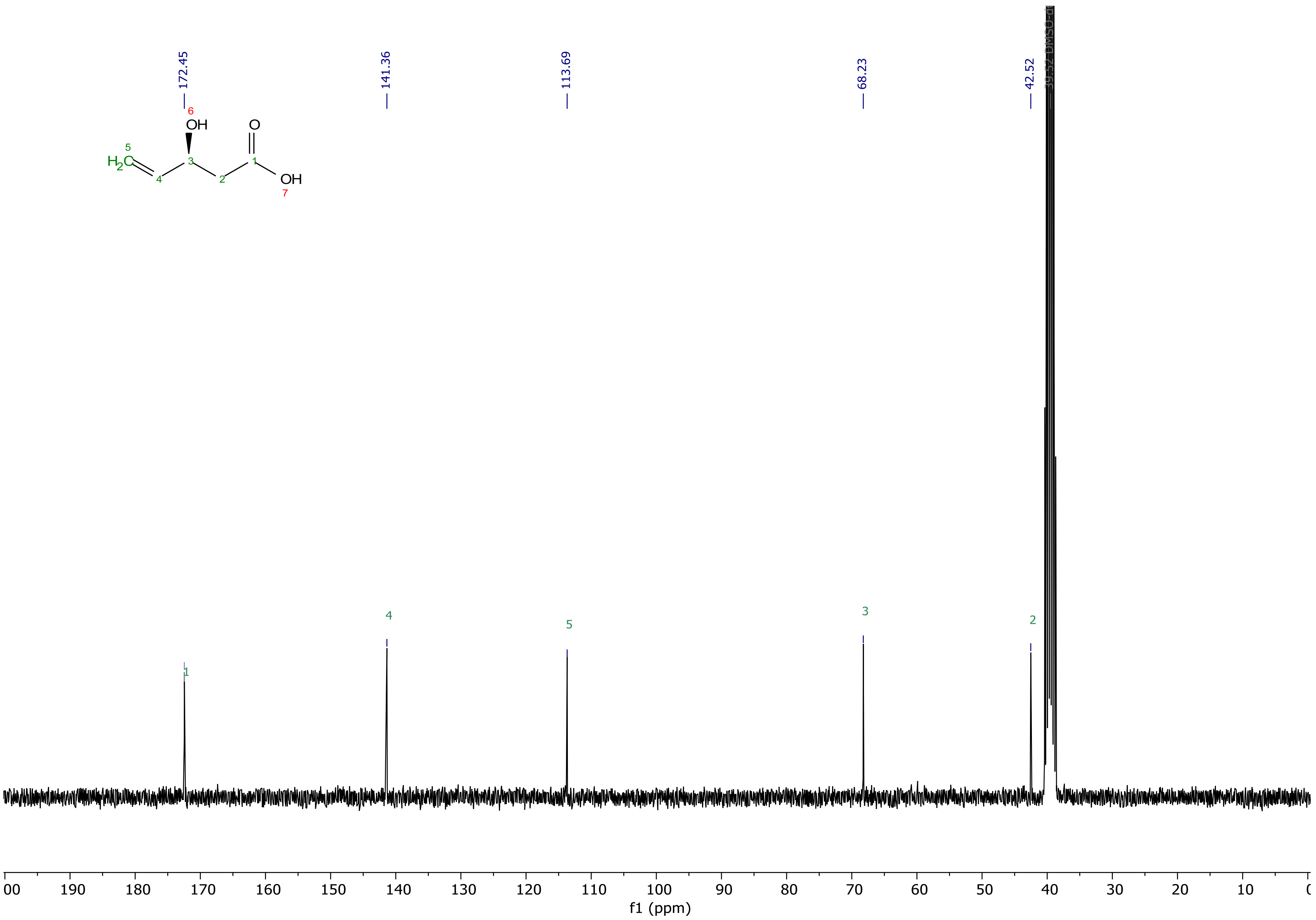

Figure 32: ^{13}C NMR spectrum of compound ***5a***

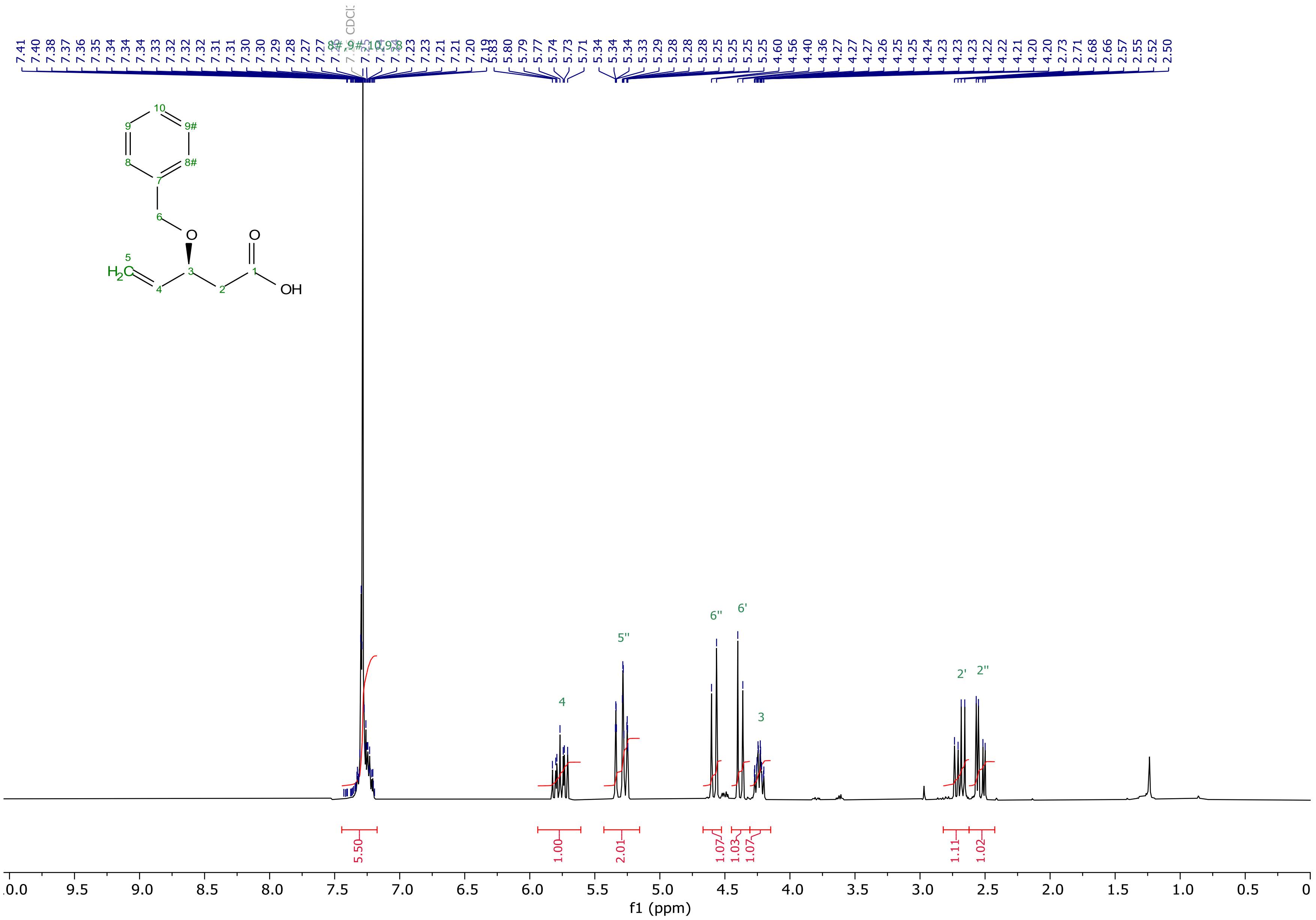

*Figure 33: ^{1}H NMR spectrum of compound **5b***

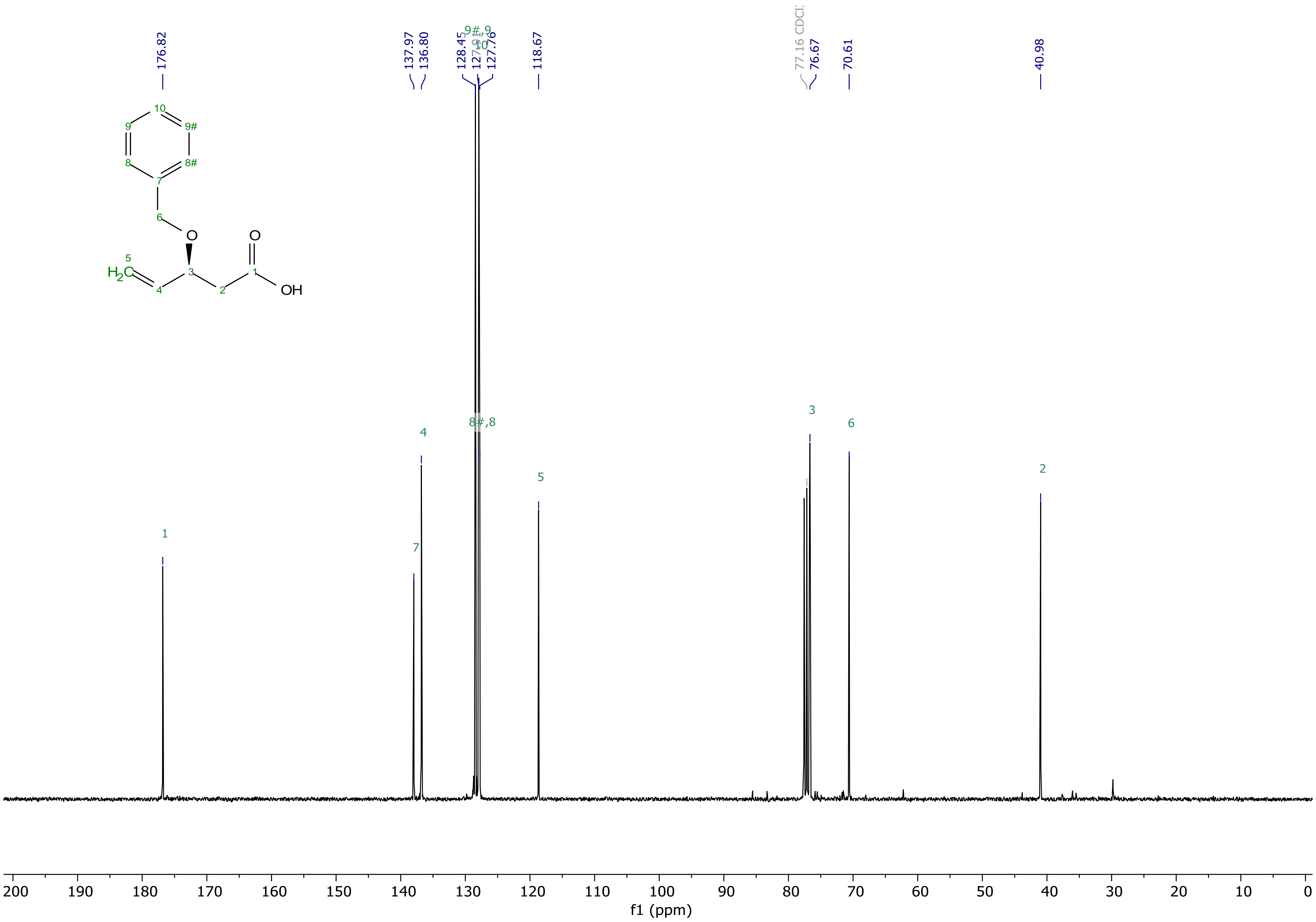

Figure 34: ^{13}*C NMR spectrum of compound* ***5b***

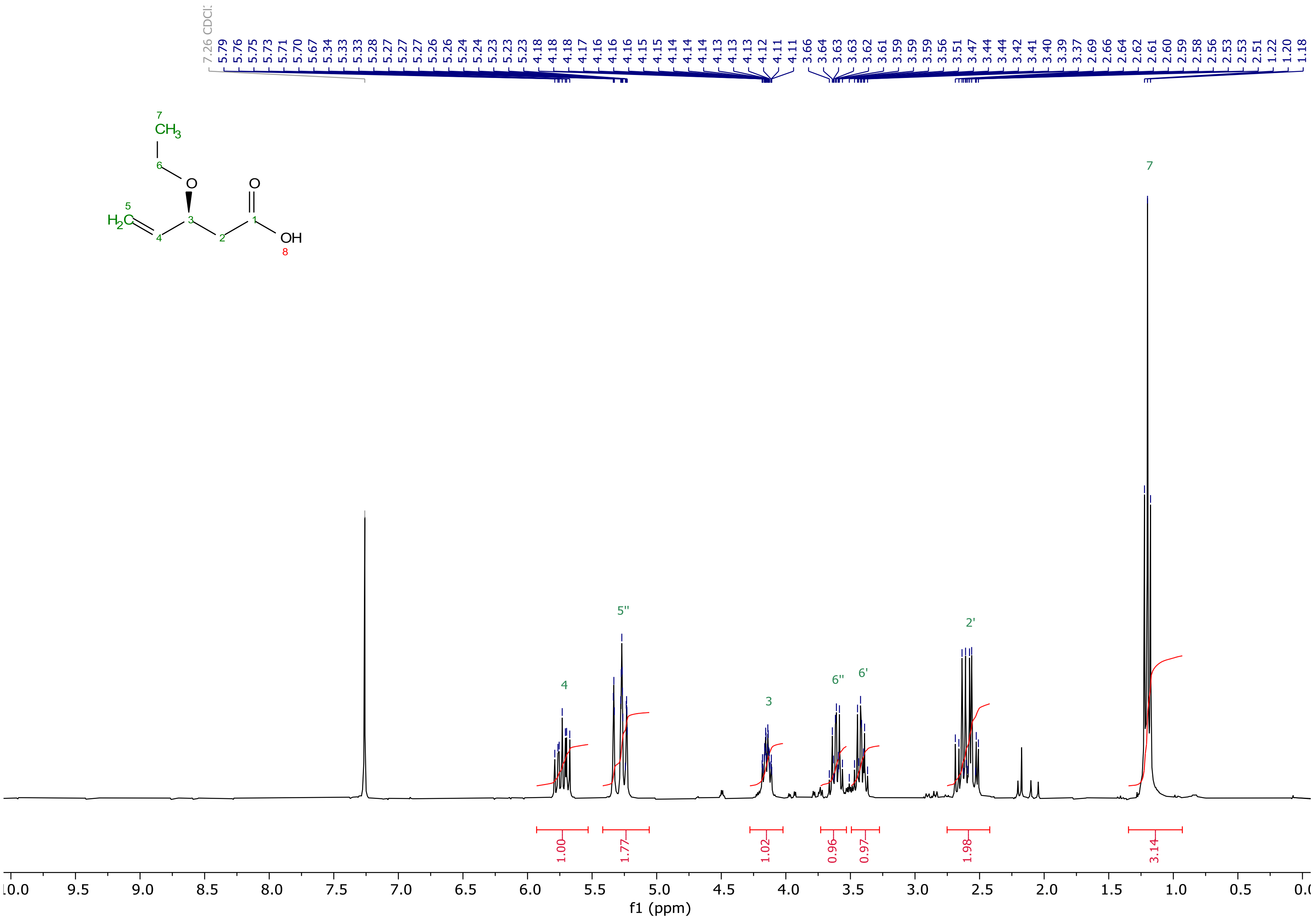

Figure 35: ¹H NMR spectrum of compound ***5c***

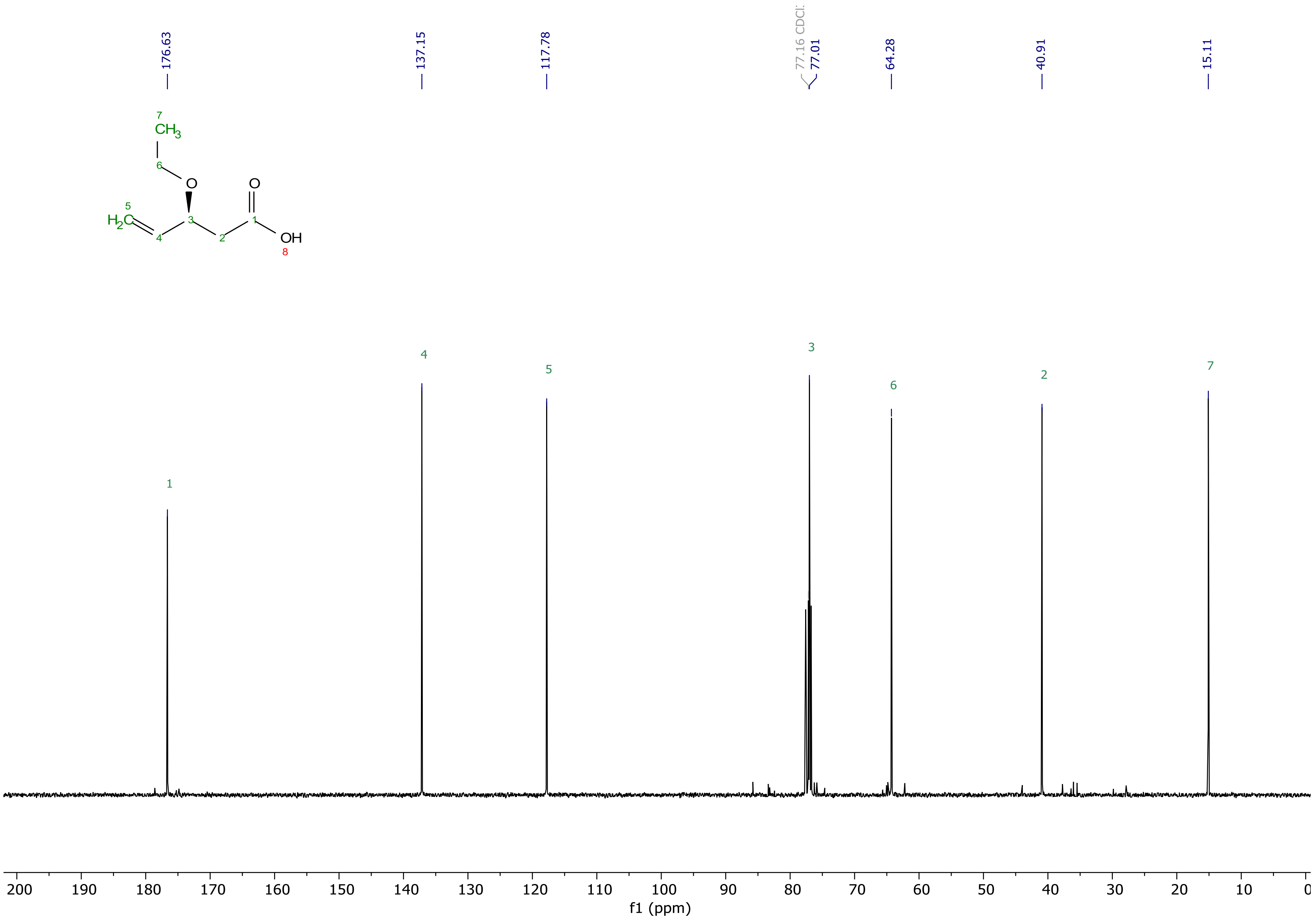

*Figure 36: ^{13}C NMR spectrum of compound **5c***

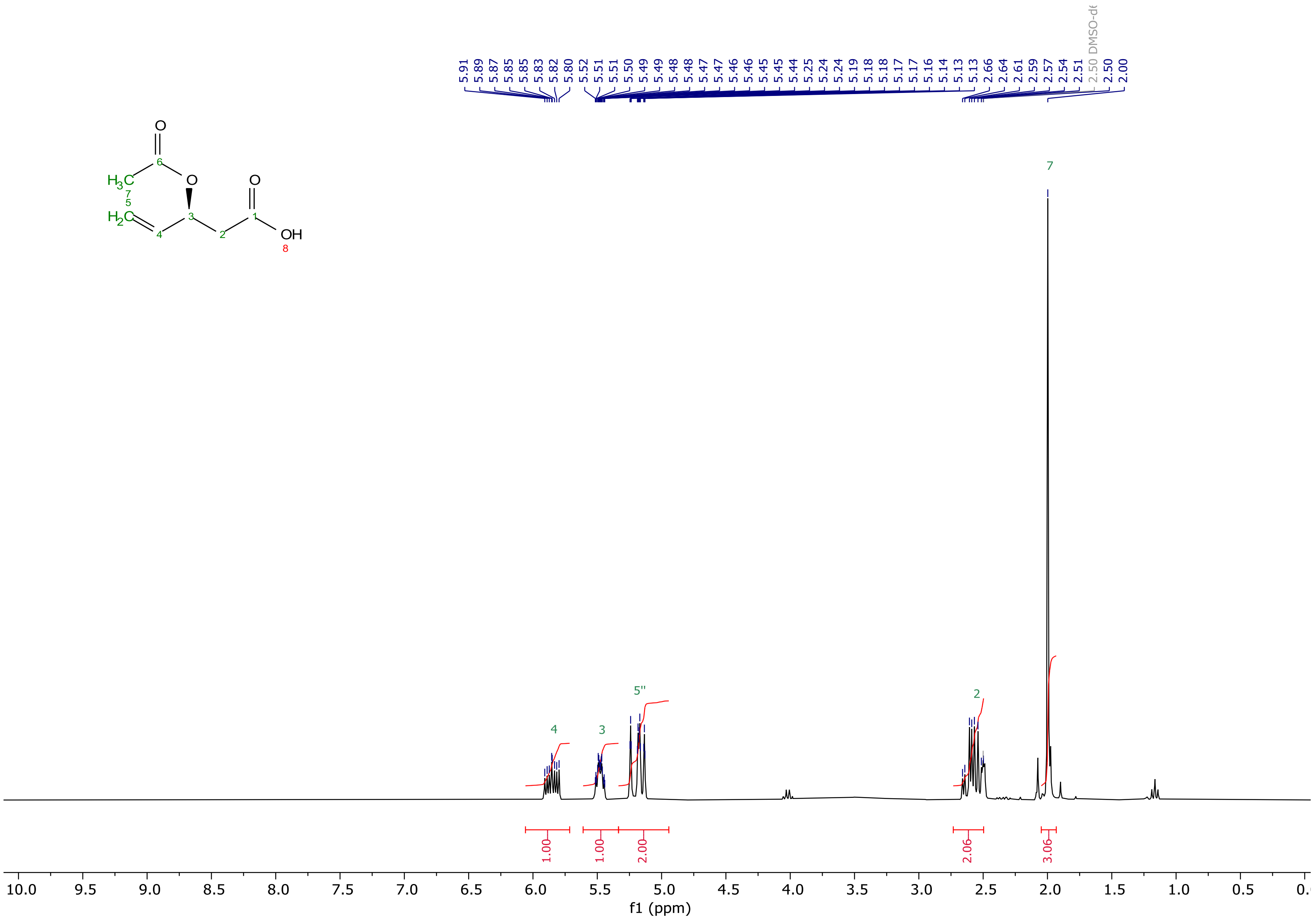

*Figure 37: ^{1}H NMR spectrum of compound **5d***

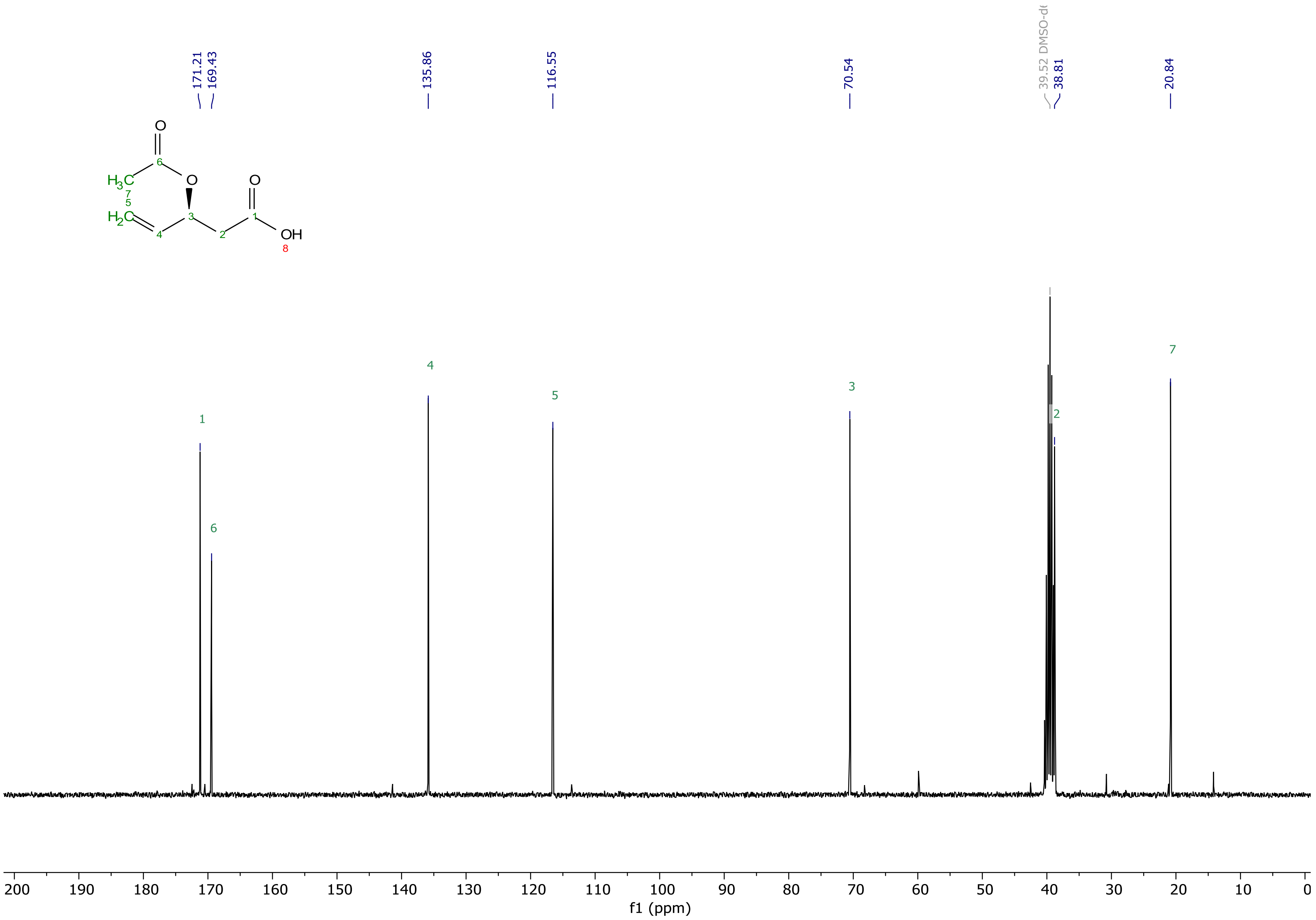

Figure 38: ^{13}C NMR spectrum of compound ***5d***

2.7. Carboxylic acid protection

Under nitrogen, pentenoic acid derivative was dissolved in DMF (C = 1.0 M), then K_2CO_3 (1.5 equiv) and benzyl bromide (1.2 equiv) were added. Reaction media was stirred at room temperature during 1 h. After completion of the reaction, salts were filtered off over Celite® pad and rinsed with EtOAc. The medium was concentrated to remove DMF. The crude product was dissolved in AcOEt, washed twice with water, then brine, dried over $MgSO_4$, filtered and concentrated. Residual traces of BnBr were removed by high vacuum pumping.

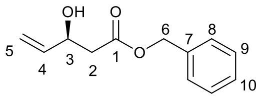

Benzyl (*S*)-3-hydroxypent-4-enoate 7a, pale yellow oil (348 mg, 84% yield)
$[\alpha]_D^{22.8}$ = +10.1 (c 0.068, Acetone). **^{1}H NMR** (300 MHz, $CDCl_3$) δ 7.36 (s, 5H, H8, H9, H10), 5.88 (ddd, $J_{4/5a}$ = 17.2 Hz, $J_{4/5b}$ = 10.5 Hz, $J_{4/3}$ = 5.5 Hz, 1H, H4), 5.31 (dt, $J_{5a/5b}$ = 1.4 Hz, 1H, H5a), 5.23 – 5.04 (m, 3H, H5b, H6), 4.57 (dddt, $J_{3/2a}$ = 8.2 Hz, $J_{3/2b}$ = 4.3, $J_{3/6}$ = 1.5 Hz, 1H, H3), 2.75 – 2.49 (m, 2H, H2) ppm. **^{13}C NMR** (75 MHz, $CDCl_3$) δ 172.2 (C1), 138.8 (C4), 135.6 (C7), 128.8 (C9,), 128.6 (C10), 128.5 (C8), 115.7 (C5), 69.1 (C3), 66.8 (C6), 41.3 (C2) ppm. **HRMS** $[M+Na]^+$ predicted 229.0835, found 229.0830.

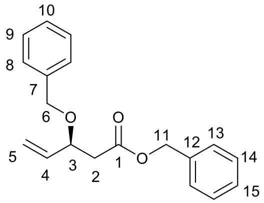

Benzyl (*S*)-3-(benzyloxy)pent-4-enoate 7b, colourless oil (243 mg, 85% yield)
$[\alpha]_D^{22.4}$ = -13.2 (c 0.015, EtOAc). **^{1}H NMR** (300 MHz, $CDCl_3$) δ 7.73-7.11 (m, 10H, H8, H9, H10, H13, H14, H15), 5.76 (ddd, $J_{4/5a}$ = 17.5 Hz, $J_{4/5b}$ = 10.3 Hz, $J_{4/3}$ = 7.6 Hz, 1H, H4), 5.34 – 5.19 (m, 2H, 5H), 5.18 – 5.02 (m, 2H, H11), 4.55 (d, $J_{6a/6b}$ = 11.6 Hz, 1H, H6a), 4.35 (d, 1H, H6b), 4.27 (td, $J_{3/2a}$ = 7.9 Hz, $J_{3/2b}$ = 5.1 Hz, 1H, H3), 2.72 (dd, $J_{2a/2b}$ = 15.1 Hz, 1H, H2a), 2.54 (dd, 1H, H2b) ppm. **^{13}C NMR** (75 MHz, $CDCl_3$): δ = 170.8 (C1), 138.3 (C7), 137.2 (C4), 136.0 (C12), 128.7-127.7 (C8, C9, C10, C13, C14, C15), 118.4 (C5), 77.2 (C3), 70.7 (C6), 66.5 (C11), 41.3 (C2) ppm. **HRMS** $[M+Na]^+$ predicted 319.1304, found 319.1312.

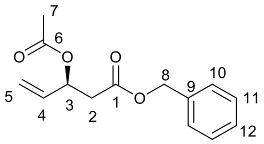

Benzyl (*S*)-3-acetoxypent-4-enoate 7d, colourless oil (897 mg, 82% yield)
$[\alpha]_D^{22.8}$ = -5.7 (c 0.082, EtOAc). **^{1}H NMR** (300 MHz, $CDCl_3$) δ 7.49-7.26 (m, 5H, H10, H11, H12), 5.83 (ddd, $J_{4/5a}$ = 17.0 Hz, $J_{4/5a}$ = 10.5 Hz, $J_{4/3}$ = 6.2 Hz, 1H, H4), 5.72-5.58 (m, 1H, H3), 5.39 – 5.16 (m, 2H, H5), 5.13 (d, $J_{8/10}$ = 1.3 Hz, 2H, H8), 2.82 – 2.59 (m, 2H, H2), 1.98 (s, 3H, H7) ppm. **^{13}C NMR** (75 MHz, $CDCl_3$): δ = 170.0 (C6), 169.8 (C1), 135.8 (C9), 135.0 (C4), 128.7 (C11), 128.5 (C-10, C12), 117.8 (C5), 70.9 (C3), 66.7 (C8), 39.6 (C2), 21.1 (C7) ppm. **HRMS** $[M+Na]^+$ predicted 271.0941, found 271.0938.

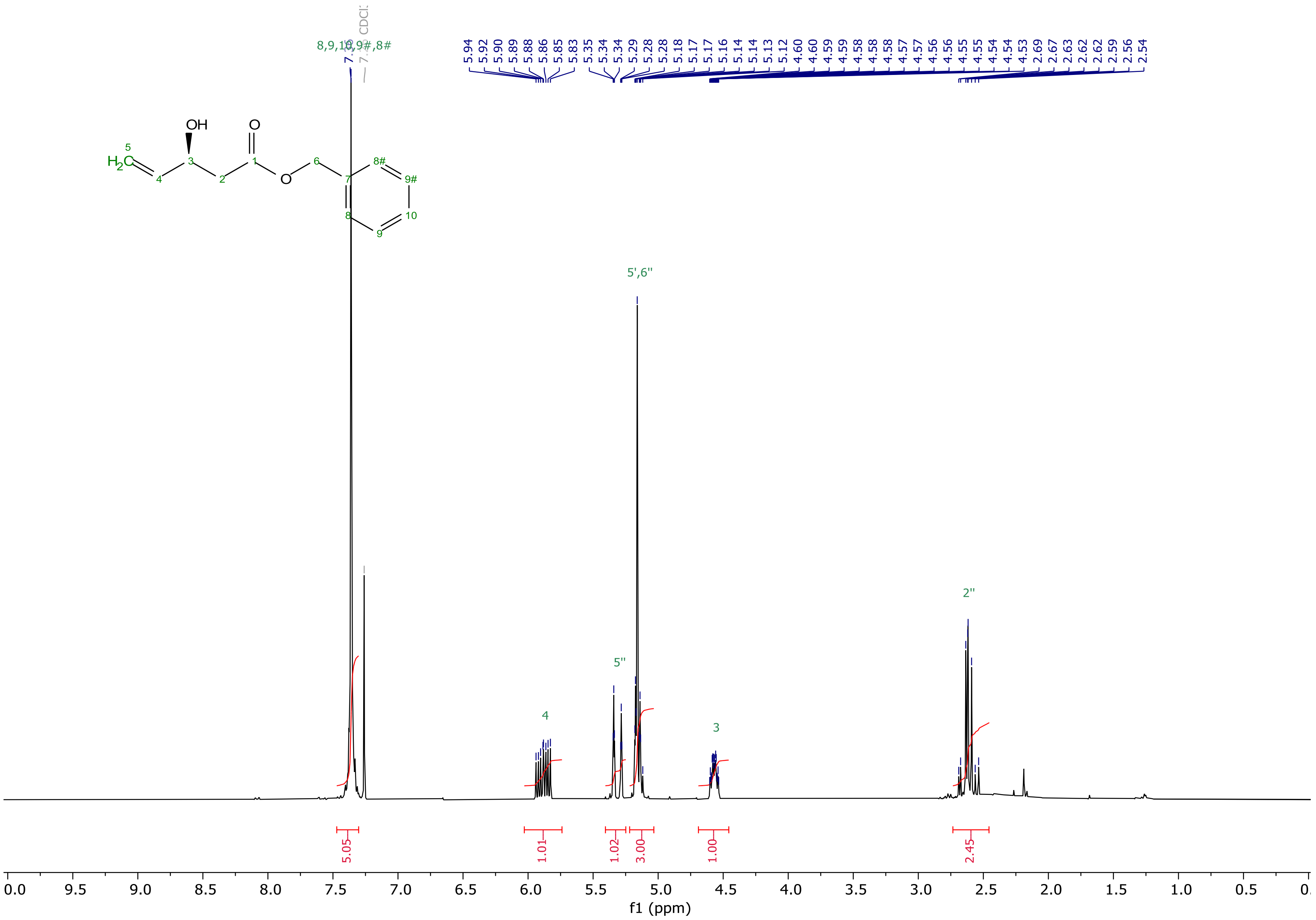

*Figure 39: ^{1}H NMR spectrum of compound **7a***

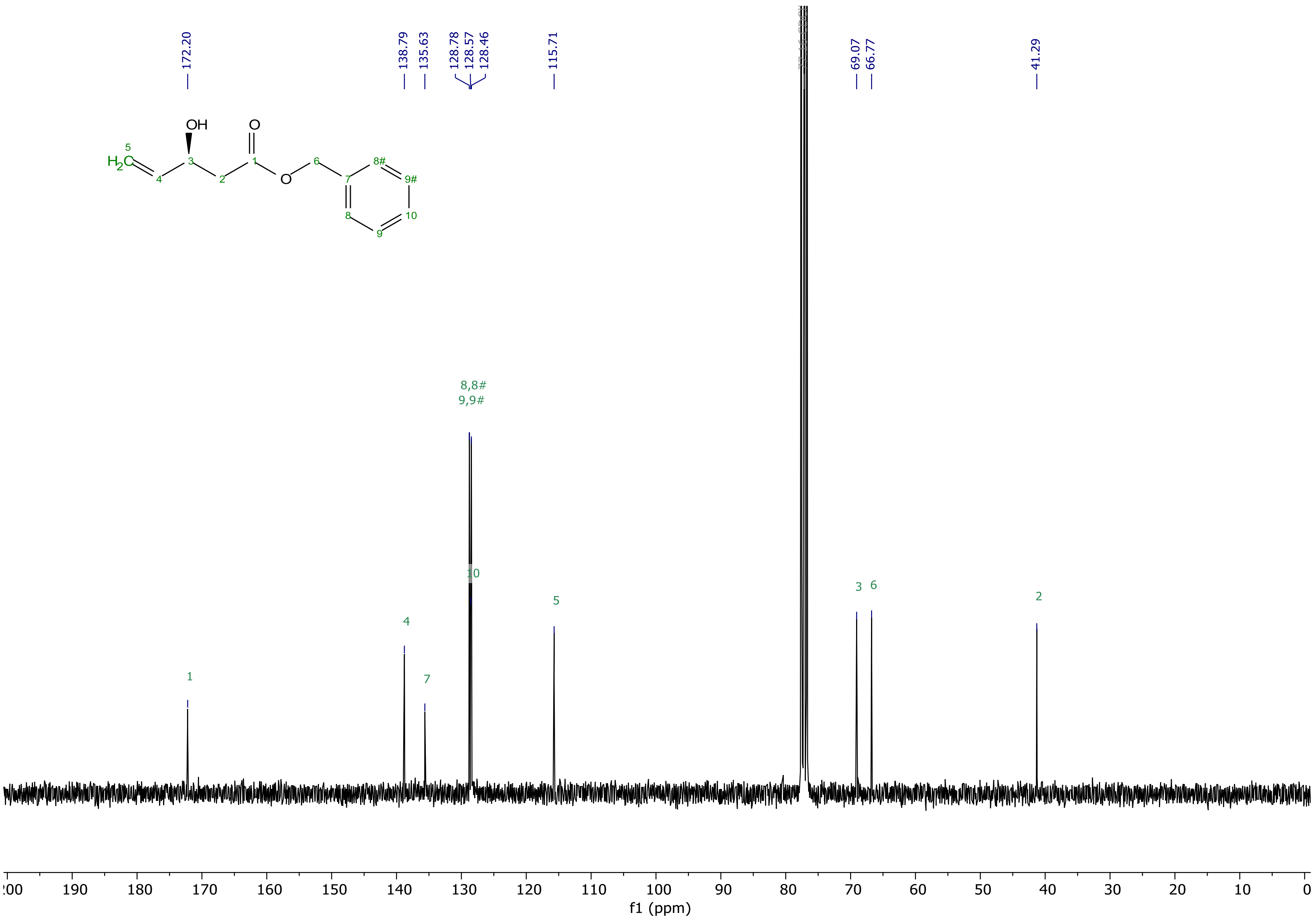

*Figure 40: ^{13}C NMR spectrum of compound **7a***

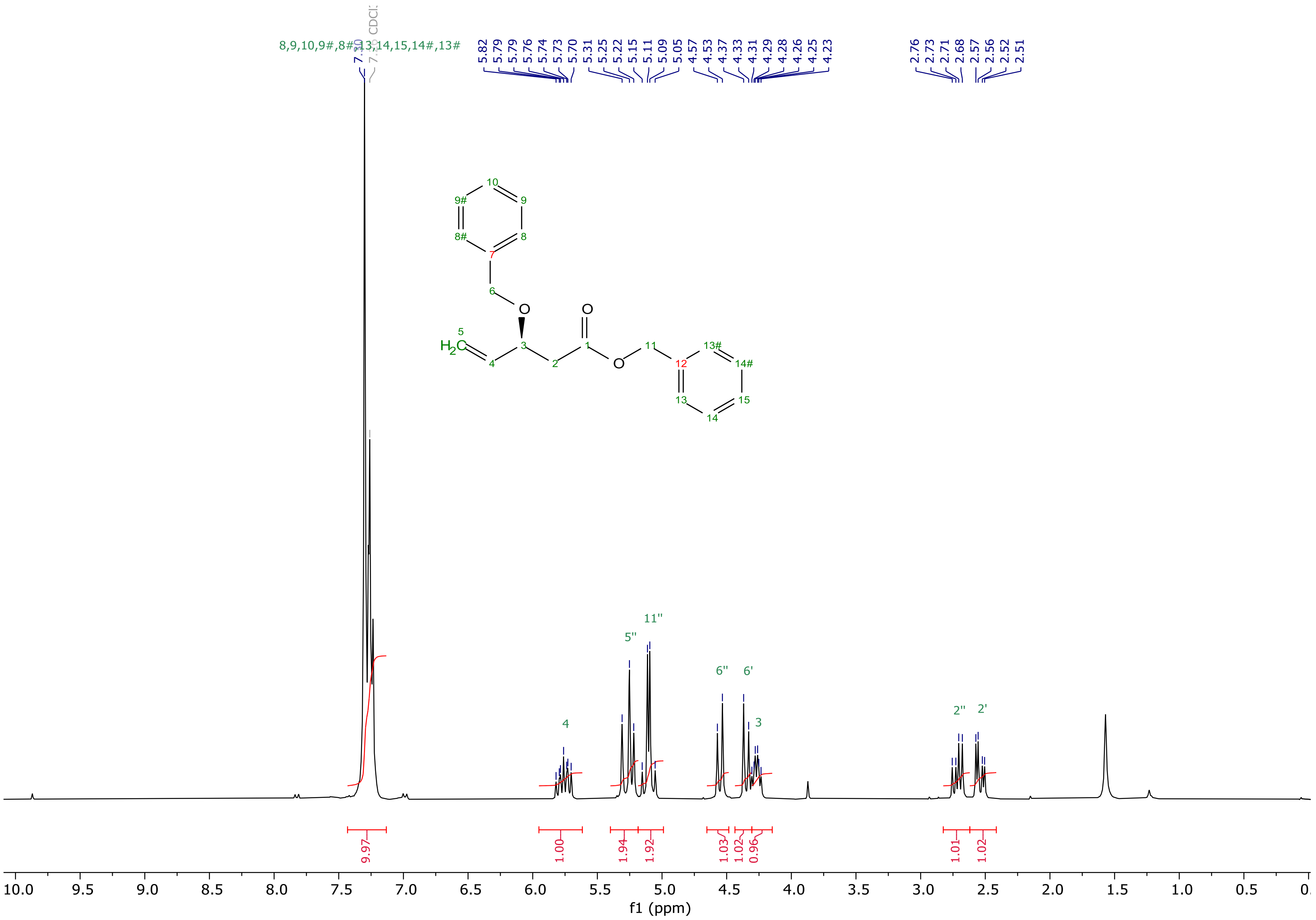

*Figure 41: ^{1}H NMR spectrum of compound **7b***

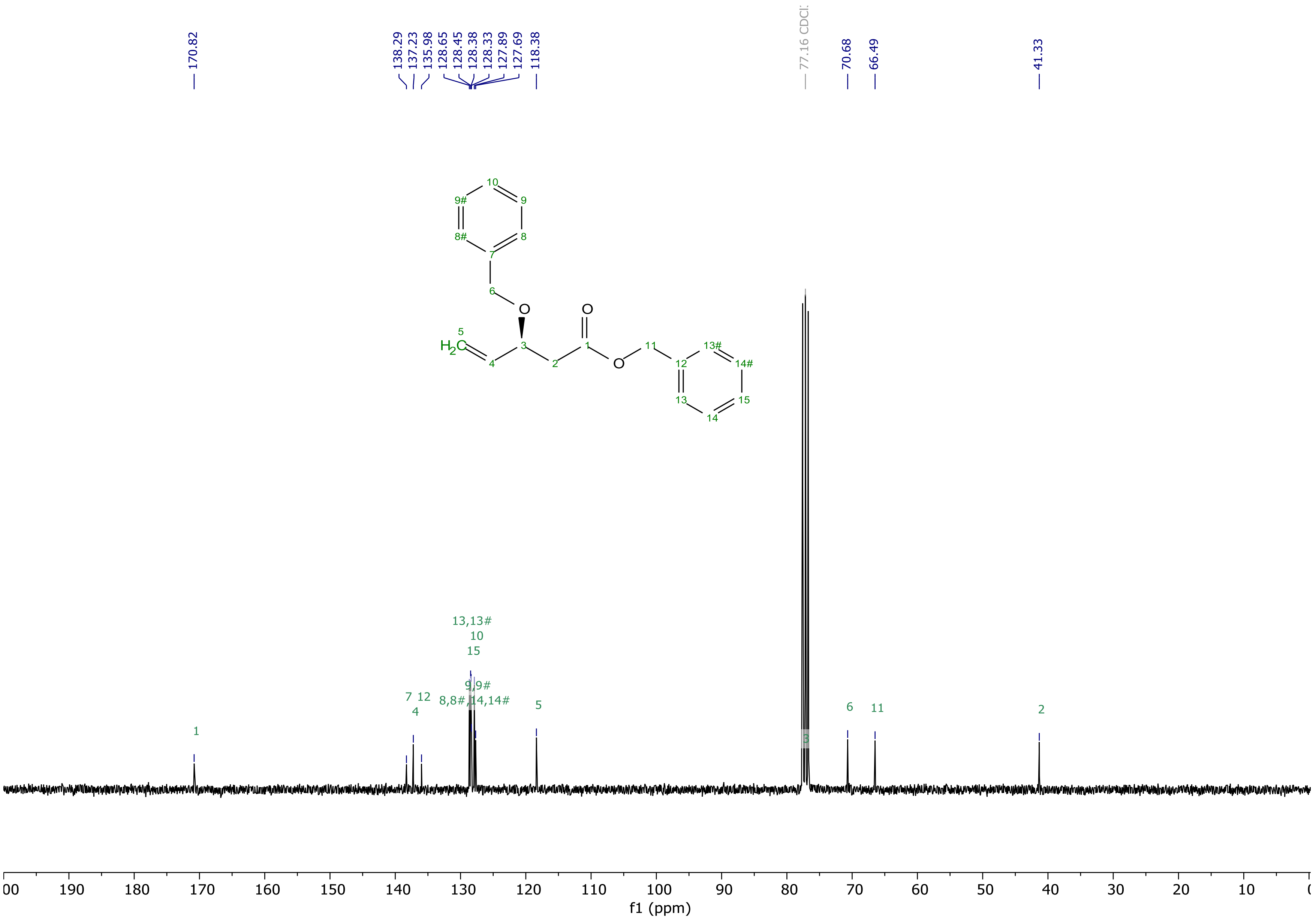

*Figure 42: ^{13}C NMR spectrum of compound **7b***

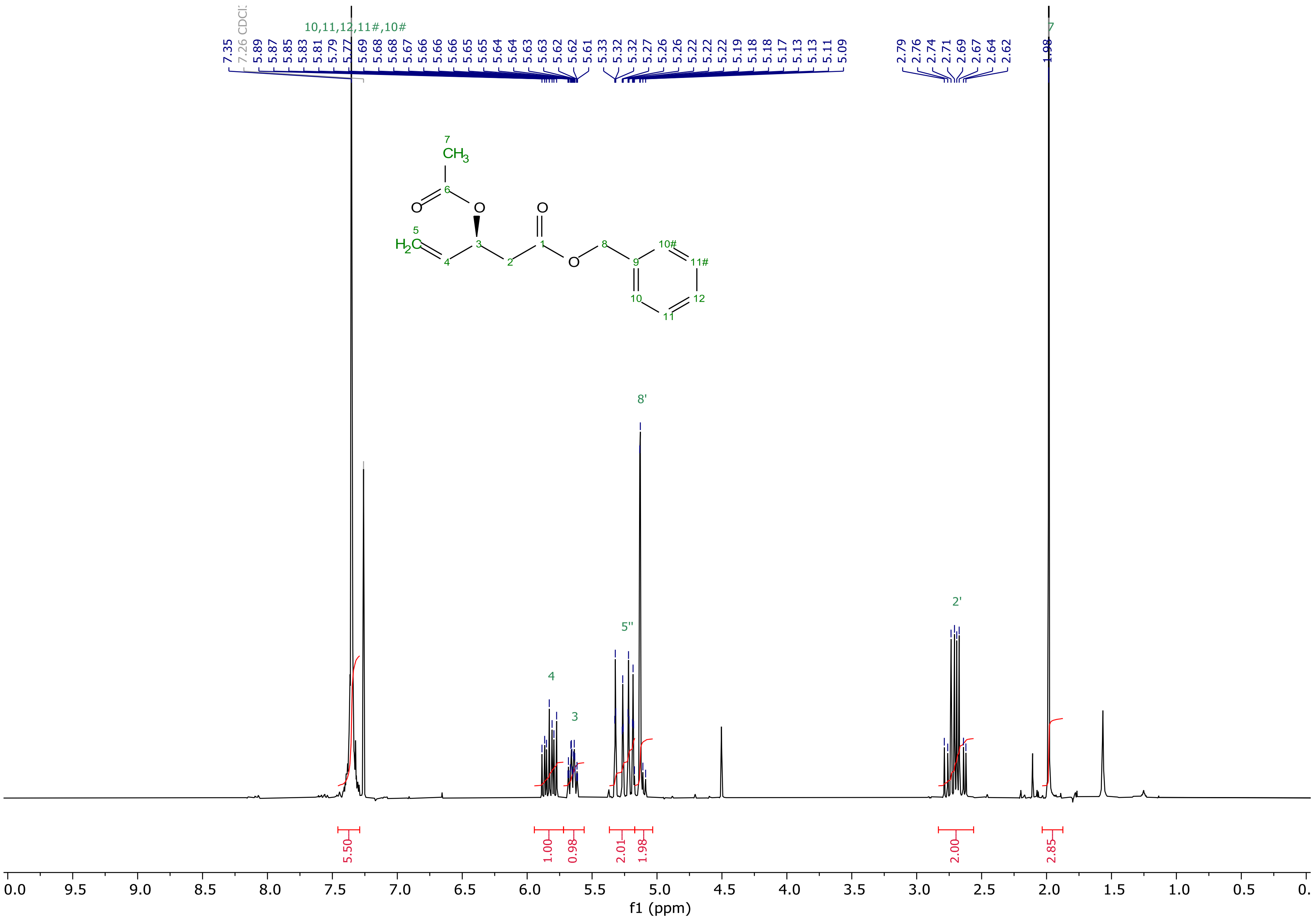

*Figure 43: ^{1}H NMR spectrum of compound **7d***

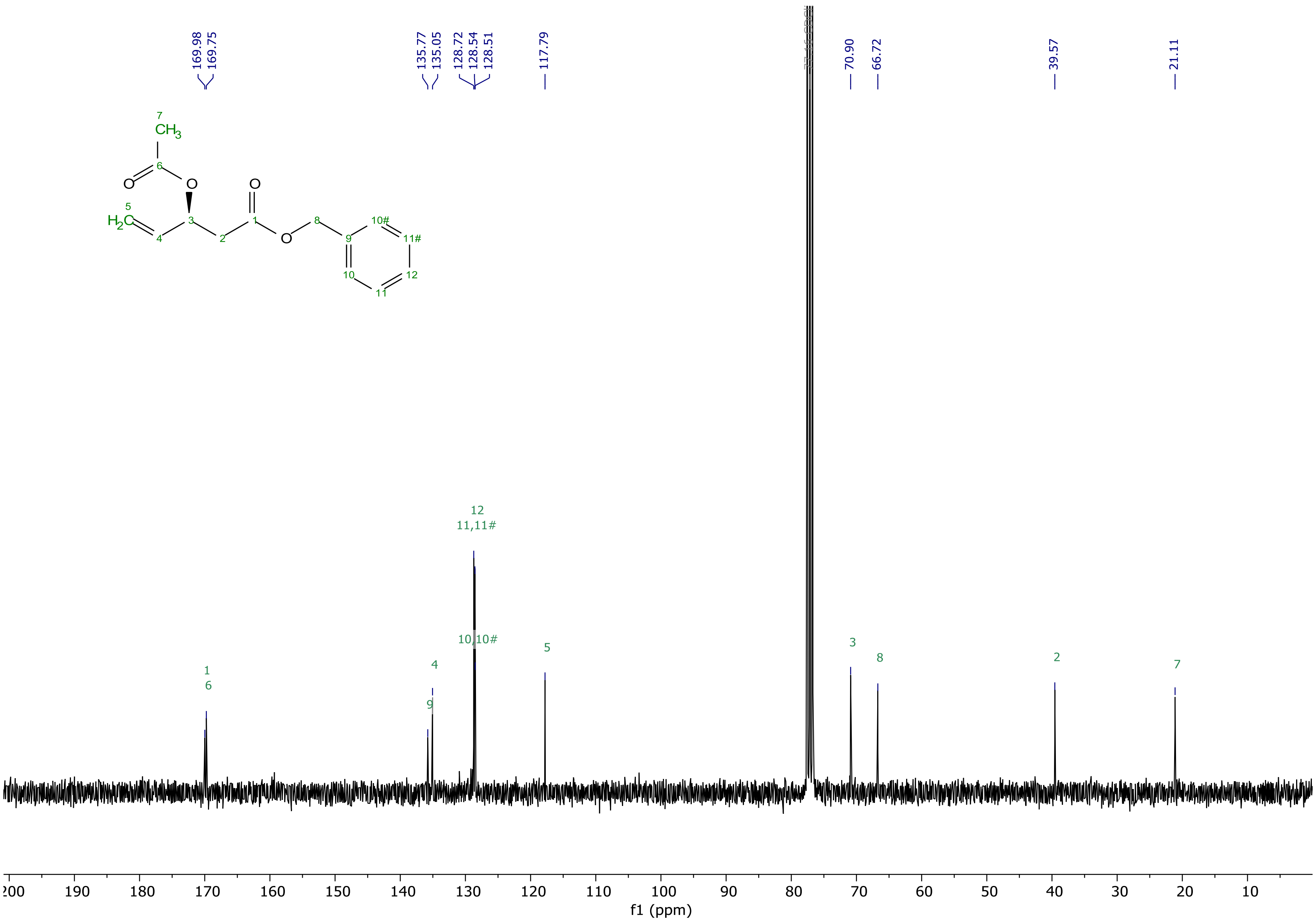

*Figure 44: ¹³C NMR spectrum of compound **7d***

2.8. General procedure for cross-metathesis

Under N_2, pentenoic acid (or benzyl pentanoate) derivative was dissolved in CPME (C = 0.1 or 0.25 M), then hept-1-ene (4 equiv.) and CuI (7 mol%) were added. The reaction mixture was stirred and heated to 40°C. Grubbs II catalyst (4.8 or 2.4 mol%) was dissolved in CPME (2 mL) and added through syringe pump over 1 hour. Then reaction was pursued for two more hours. Products were adsorbed on silica and purified by flash chromatography over silica gel. If needed, residual ruthenium traces can be removed by adsorption on activated charcoal in ethanol.

(*S,E*)-3-hydroxydec-4-enoic acid 6a, Purification 70/30 cyclohexane/EtOAc (126 mg, 46% yield), colourless oil

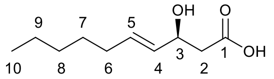

$[\alpha]_D^{21.0}$ = +3.6 (c 0.025, EtOAc). **^{1}H NMR** ($CDCl_3$, 300 MHz): δ = 5.72 (dt, $J_{5/4}$ = 15.3 Hz, $J_{5/6}$ = 6.7 Hz, 1H, H-5), 5.47 (ddt, $J_{4/3}$ = 6.6 Hz, $J_{4/2}$ = 1.5 Hz, 1H, H-4), 4.50 (q (ddd), $J_{3/2}$ = 6.3 Hz, 1H, H-3), 2.65-2.54 (m, 2H, H-2), 2.12-1.97 (m, 2H, H-6), 1.49-1.10 (m, 6H, H-7, H-8 and H-9), 0.86 (t, $J_{10/9}$ = 6.6 Hz, 3H, H-10) ppm. **^{13}C NMR** (75 MHz, $CDCl_3$): δ = 176.7 (C-1), 133.7 (C-5), 130.1 (C-4), 69.0 (C-3), 40.4 (C-2), 32.2 (C-6), 31.5 (C-8), 28.8 (C-7), 22.6 (C-9), 14.2 (C-10) ppm. **HRMS** $[M-H]^-$ predicted 185.1183, found 185.1185.

Benzyl (*S,E*)-3-hydroxydec-4-enoate 8a, Purification 95/5 cyclohexane/EtOAc (124 mg, 59% yield), colourless oil.

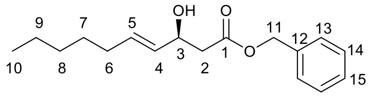

$[\alpha]_D^{21.0}$ = +4.5 (c 0.036, EtOAc). **^{1}H NMR** ($CDCl_3$, 300 MHz): δ = 7.39-7.32 (m, 5H, H-13, H-14 and H-15), 5.71 (dt, $J_{5/4}$ = 15.3, $J_{5/6}$ = 6.6 Hz, $J_{5/3}$ =1.1 Hz 1H, H-5), 5.47 (ddt, $J_{4/3}$ = 6.6 Hz, $J_{4/2}$ = 1.5 Hz, 1H, H-4), 5.15 (s, 2H, H-11), 4.51 (q (ddd), J = 6.3 Hz, 1H, H-3), 2.62-2.54 (m, 2H, H-2), 2.00 (q (dt), $J_{6/7}$ = $J_{6/5}$ = 6.8 Hz, 2H, H-6), 1.40-1.22 (m, 6H, H-7, H-8 and H-9), 0.88 (t, $J_{10/9}$ = 6.7 Hz, 3H, H-10) ppm. **^{13}C NMR** (75 MHz, $CDCl_3$): δ = 172.3 (C-1),135.7 (C-12), 133.2 (C-5), 130.4 (C-4), 128.8 (C-14), 128.5 (C-15), 128.4 (C-13), 69.1 (C-3), 66.7 (C-11), 41.8 (C-2), 32.3 (C-6), 31.5 (C-8), 28.8 (C-7), 22.6 (C-9), 14.2 (C-10) ppm. **HRMS** $[M+H]^+$ predicted 229.0835, found 229.0830.

(*S,E*)-3-acetoxydec-4-enoic acid 6d, Purification 95/5 cyclohexane/EtOAc (104 mg, 62% yield), colourless oil

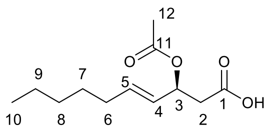

$[\alpha]_D^{24.4}$ = -23.1 (c 0.027, Acetone). **^{1}H NMR** ($CDCl_3$, 300 MHz): δ = 5.84-5.66 (m, 1H, H-5), 5.63-5.51 (m, 1H, H-3), 5.47-5.35 (m, 1H, H-4), 2.72-2.42 (m, 2H, H-2), 2.03-1.95 (m, 5H, H-6 and H-12), 1.39-1.22 (m, 6H, H-7, H-8 and H-9), 0.85 (t, $J_{10/9}$ = 6.7 Hz, 3H, H-10) ppm. **^{13}C NMR** (75 MHz, $CDCl_3$): δ = 175.5 (C-1), 170.5 (C-11), 135.3 (C-5), 126.8 (C-4), 71.3 (C-3), 32.2 (C-2 and C-6), 31.4 (C-8), 28.6 (C-7), 22.5 (C-9), 21.3 (C-12), 14.1 (C-10) ppm. **HRMS** $[M-H]^-$ predicted 227.1289, found 227.1289.

Benzyl (*S,E*)-3-acetoxydec-4-enoate 8d, Purification 98/2 cyclohexane/EtOAc (38% yield), colourless oil.

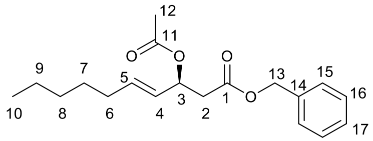

$[\alpha]_D^{21.0}$ = -18.6 (c 0.027, EtOAc). **^{1}H NMR** ($CDCl_3$, 300 MHz): δ = 7.45-7.28 (m, 5H, H-15, H-16 and H-17), 5.83-5.68 (m 1H, H-5), 5.62-5.56 (m, 1H, H-3), 5.45-5.37 (m, 1H, H-4), 5.12 (s, 2H, H-13), 2.86-2.46 (m, 2H, H-2), 1.98-1.92 (m, 5H, H-6 and H-12), 1.37-1.15 (m, 6H, H-7, H-8 and H-9) 0.85 (t, $J_{10/9}$ = 6.9 Hz, 3H, H-10) ppm. **^{13}C NMR** (75 MHz, $CDCl_3$): δ = 170.1 (C-11), 169.9 (C-1), 135.8 (C-5 and C-14), 128.7 (C-16), 128.5 (C-15 and C-17), 126.6 (C-4), 71.1 (C-3), 66.6 (C-13), 40.0 (C-2), 32.2 (C-6), 31.5 (C-8), 28.6 (C-7), 22.6 (C-9), 21.3 (C-12), 14.2 (C-10) ppm. **HRMS** $[M+Na]^+$ predicted 341.1723, found 341.1708.

(*S,E*)-3-(benzyloxy)dec-4-enoic acid 6b, Purification 90/10 cyclohexane/EtOAc, (88 mg, 47% yield), colourless oil

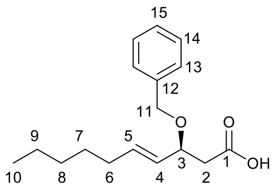

$[\alpha]_D^{23.9}$ = -16.8 (c 0.0072, Acetone). **^{1}H NMR** ($CDCl_3$, 300 MHz): δ = 7.45-7.28 (m, 5H, H13-14-15), 5.76 (dt, $J_{5/4}$ = 14.3, $J_{5/6}$ = 6.8 Hz, 1H, H-5), 5.37 (dd, $J_{4/3}$ = 8.4 Hz, 1H, H-4), 4.62 (d, J = 11.6 Hz, 1H, H-11a), 4.39 (d, J = 11.6 Hz, 1H, H11b), 4.27-4.14 (m, 1H, H-3), 2.70 (dd, $J_{2a/2b}$ = 15.5 Hz, $J_{2a/3}$ = 8.2 Hz, 1H, H-2a), 2.57 (dd, $J_{2b/3}$ = 4.7 Hz, 1H, H-2b), 2.07 (q, J = 7.1 Hz, 2H, H-6), 1.45-1.22 (m, 6H, H-7-8-9), 0.94-0.84 (m, 3H, H-10) ppm. **^{13}C NMR** (75 MHz, $CDCl_3$): δ = 176.4 (C-1), 137.8 (C-12), 136.8 (C-5), 128.6 (C-14), 128.5 (C-15), 128.0 (C-13), 127.8 (C-4), 76.5 (C-3), 70.3 (C-11), 41.0 (C-2), 32.3 (C-6), 31.5 (C-8), 28.8 (C-7), 22.6 (C-9), 14.2 (C-10) ppm. **HRMS** $[M-H]^-$ predicted 275.1652, found 275.1652.

Benzyl (*S,E*)-3-(benzyloxy)dec-4-enoate 8b, Purification 100% cyclohexane (149 mg, 66% yield)

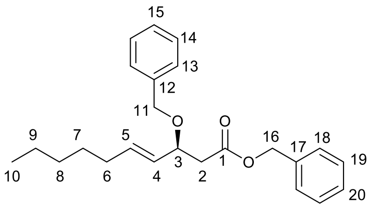

[α]$_D$$^{21.9}$ = -21.0 (c 0.014, EtOAc). **^{1}H NMR** ($CDCl_3$, 300 MHz): δ = 7.34-7.22 (m, 10H, H-13, H-14, H-15, H-18, H-19 and H-20), 5.68 (dq, $J_{5/4}$ = 14.7, $J_{5/3}$ = 8.0, $J_{5/6}$ = 7.3 Hz, 1H, H-5), 5.35 (dd, $J_{4/3}$ = 8.3 Hz, 1H, H-4), 5.19-5.03 (m, 2H, H-16), 4.54 (d, $J_{11a/11b}$ = 11.7 Hz, 1H, H-11a), 4.33 (d, 1H, H-11b), 4.29-4.08 (m, 1H, H-3), 2.72 (dd, $J_{2a/2b}$ = 14.9, $J_{2a/3}$ = 8.3 Hz, 1H, H-2a), 2.59-2.41 (m, 1H, H-2b), 2.03 (q, $J_{6/7}$ = 7.0 Hz, 2H, H-6), 1.45-1.16 (m, 6H, H-7-8-9), 0.88 (t, $J_{10/9}$ = 6.7 Hz, 3H, H-10) ppm. **^{13}C NMR** (75 MHz, $CDCl_3$): δ = 171.0 (C-1), 138.5 (C-12), 136.1 (C-17), 127.7-128.5 (C-13, C-14, C-15, C-18, C-19 and C-20), 76.9 (C-3), 70.2 (C-11), 66.4 (C-16), 41.7 (C-2), 32.3 (C-6), 31.5 (C-8), 28.9 (C7), 22.6 (C-9), 14.2 (C-10) ppm. **HRMS** [M+Na]$^+$ predicted 389.2087, found 389.2085.

(*S,E*)-3-ethoxydec-4-enoic acid 6c, Purification 95/5 cyclohexane/EtOAc (130 mg, 60% yield), brown oil

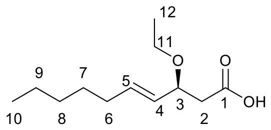

[α]$_D$$^{24.2}$ = +2.5 (c 0.008, EtOAc). **^{1}H NMR** ($CDCl_3$, 300 MHz): δ = 5.71 (dt, $J_{5/4}$ = 14.4 Hz, $J_{5/6}$ = 6.8 Hz, 1H, H-5), 5.30 (dd, $J_{4/3}$ = 8.2 Hz, 1H, H-4), 4.09 (dt, $J_{3/2}$ = 4.7 Hz, 1H, H-3), 3.69-3.52 (m, 1H, H-11a), 3.37 (dt, $J_{11b/3}$ = 9.3 Hz, $J_{11b/11a}$ = 6.8 Hz, 1H, H-11b), 2.72-2.42 (m, 2H, H-2), 2.07-2.01 (m, 2H, H-6), 1.49-1.09 (m, 9H, H-7, H-8, H-9 and H-12), 0.88 (t, $J_{10/9}$ = 6.7 Hz, 3H, H-10) ppm. **^{13}C NMR** (75 MHz, $CDCl_3$): δ = 174.6 (C-1), 135.9 (C-5), 128.3 (C-4), 77.4 (C-3), 63.9 (C-11), 41.1 (C-2), 32.2 (C-6), 31.4 (C-8), 28.8 (C-7), 22.6 (C-9), 15.2 (C-12), 14.2 (C-10) ppm. **HRMS** [M-H]$^-$ predicted 213.1496, found 213.1496.

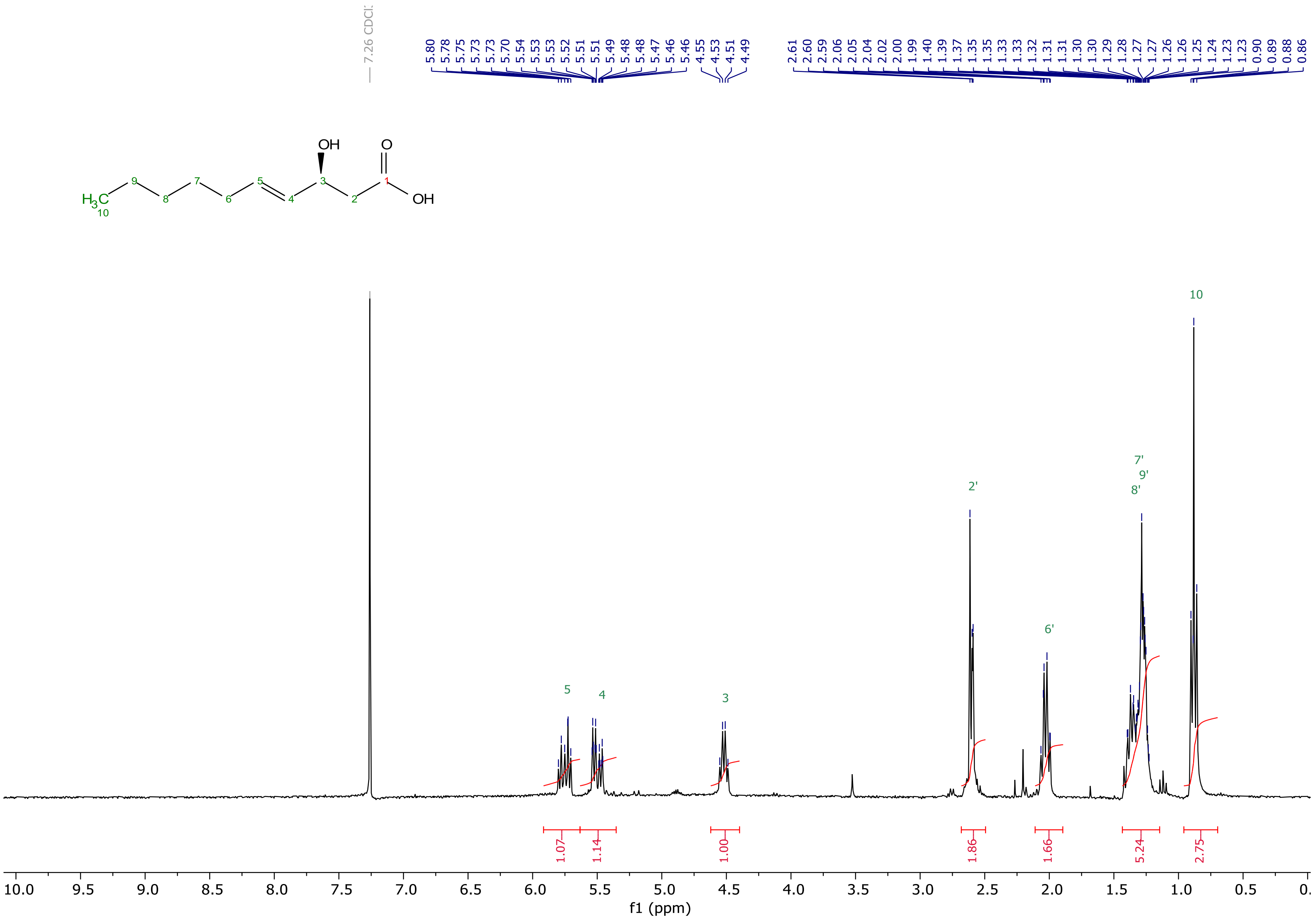

Figure 45: ^{1}H NMR spectrum of compound 6a

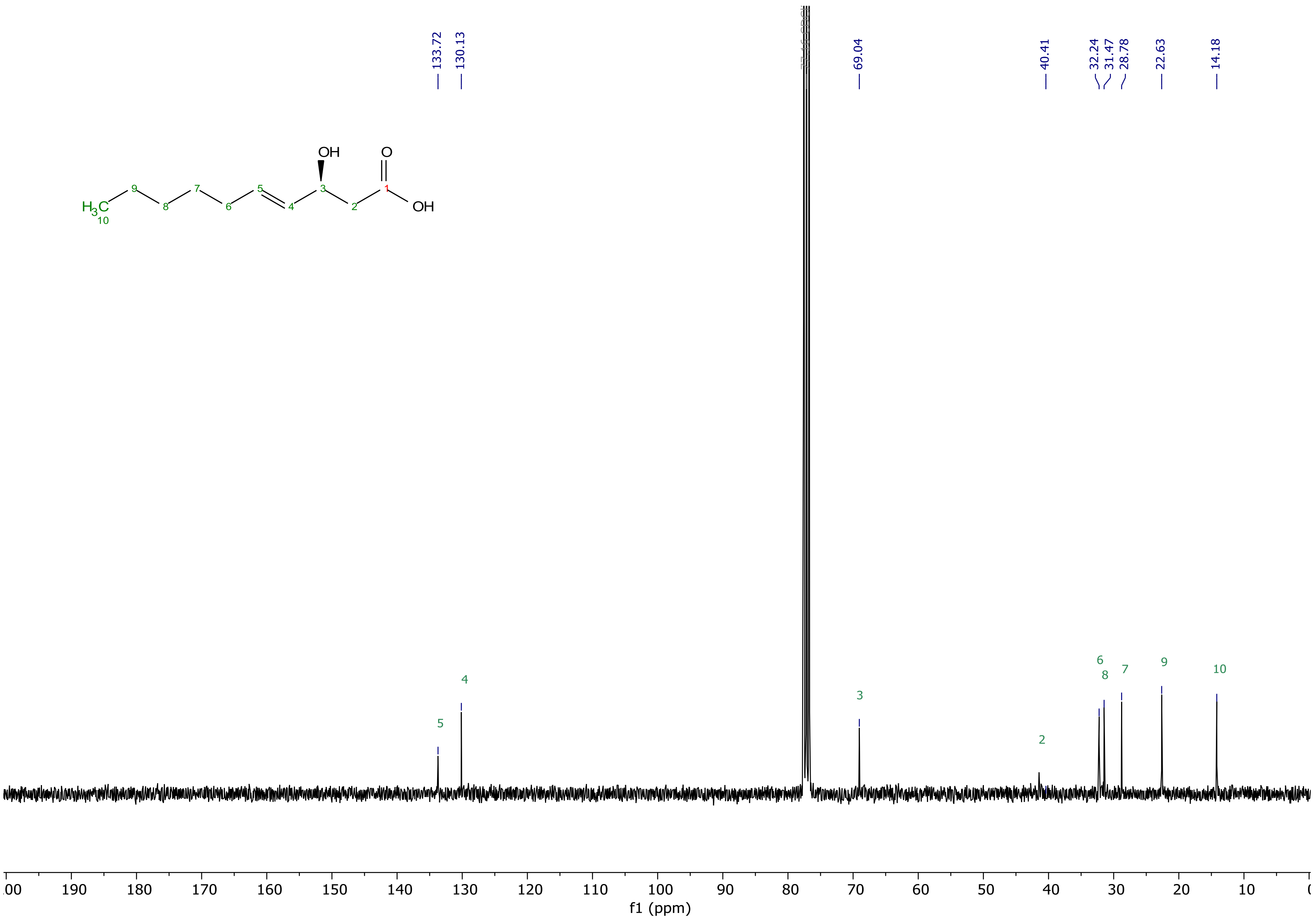

Figure 46: ^{13}C NMR spectrum of compound 6a

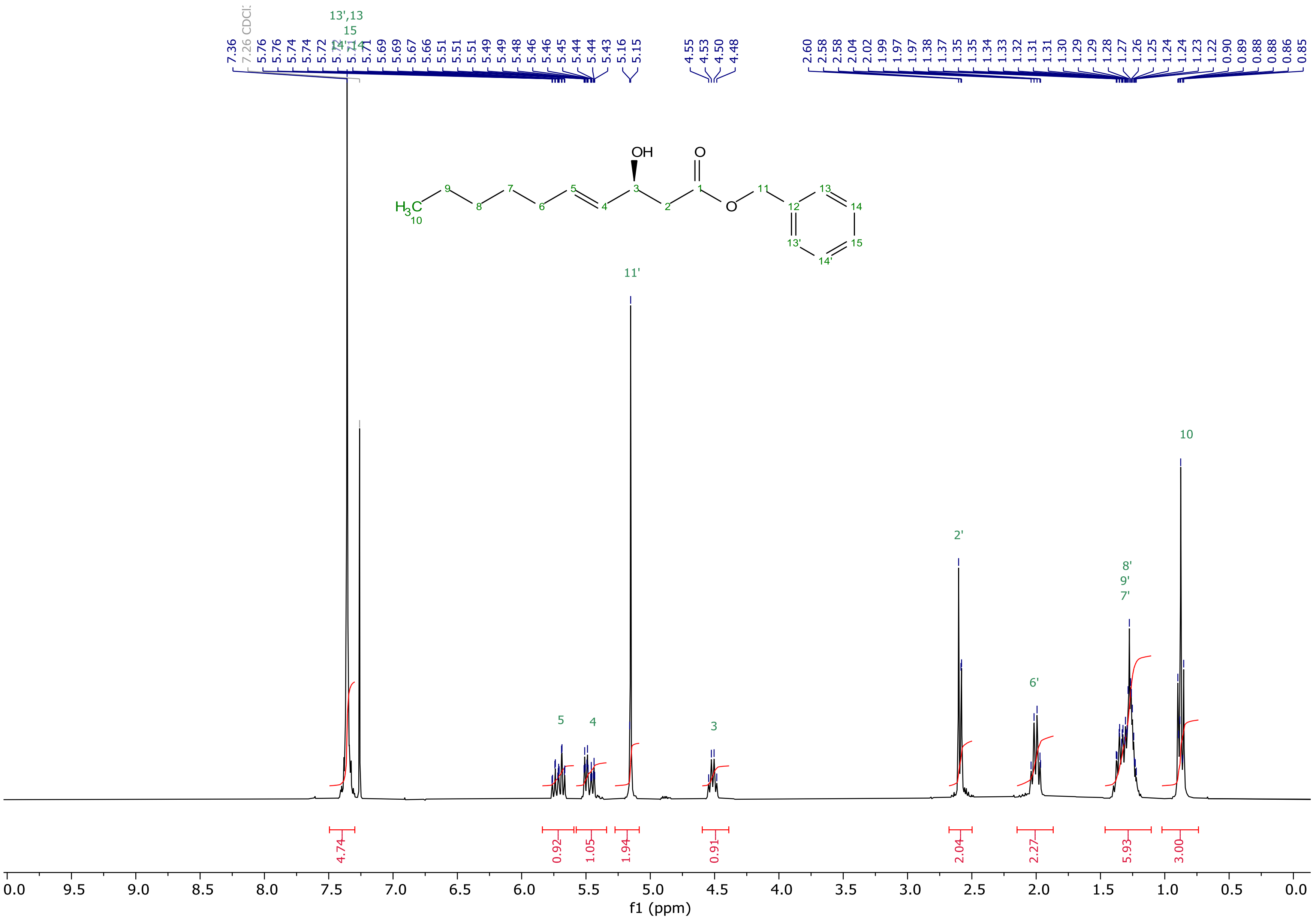

Figure 47: ^{1}H NMR spectrum of compound 8a

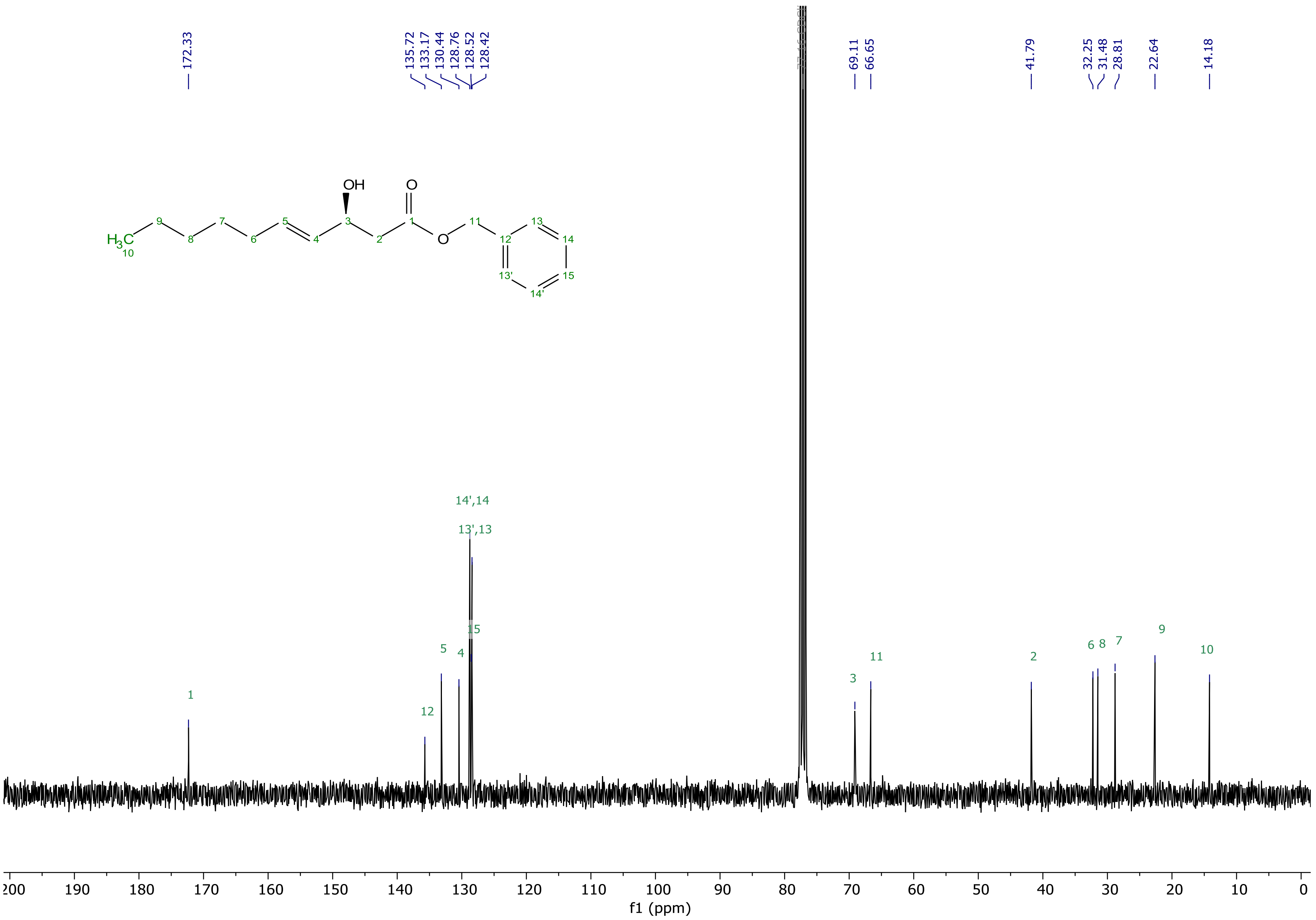

Figure 48: ^{13}C NMR spectrum of compound 8a

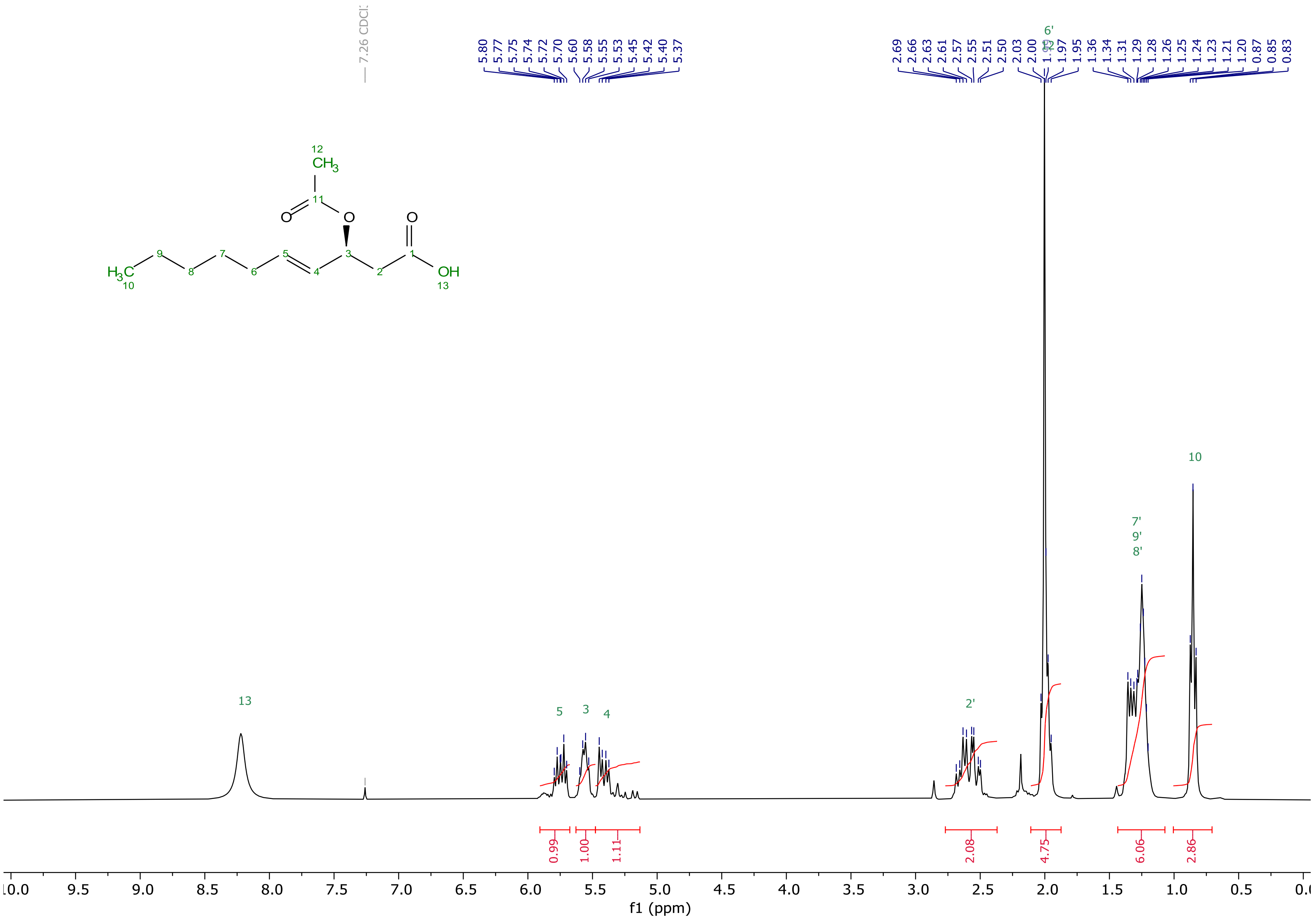

Figure 49: ^{1}H NMR spectrum of compound 6d

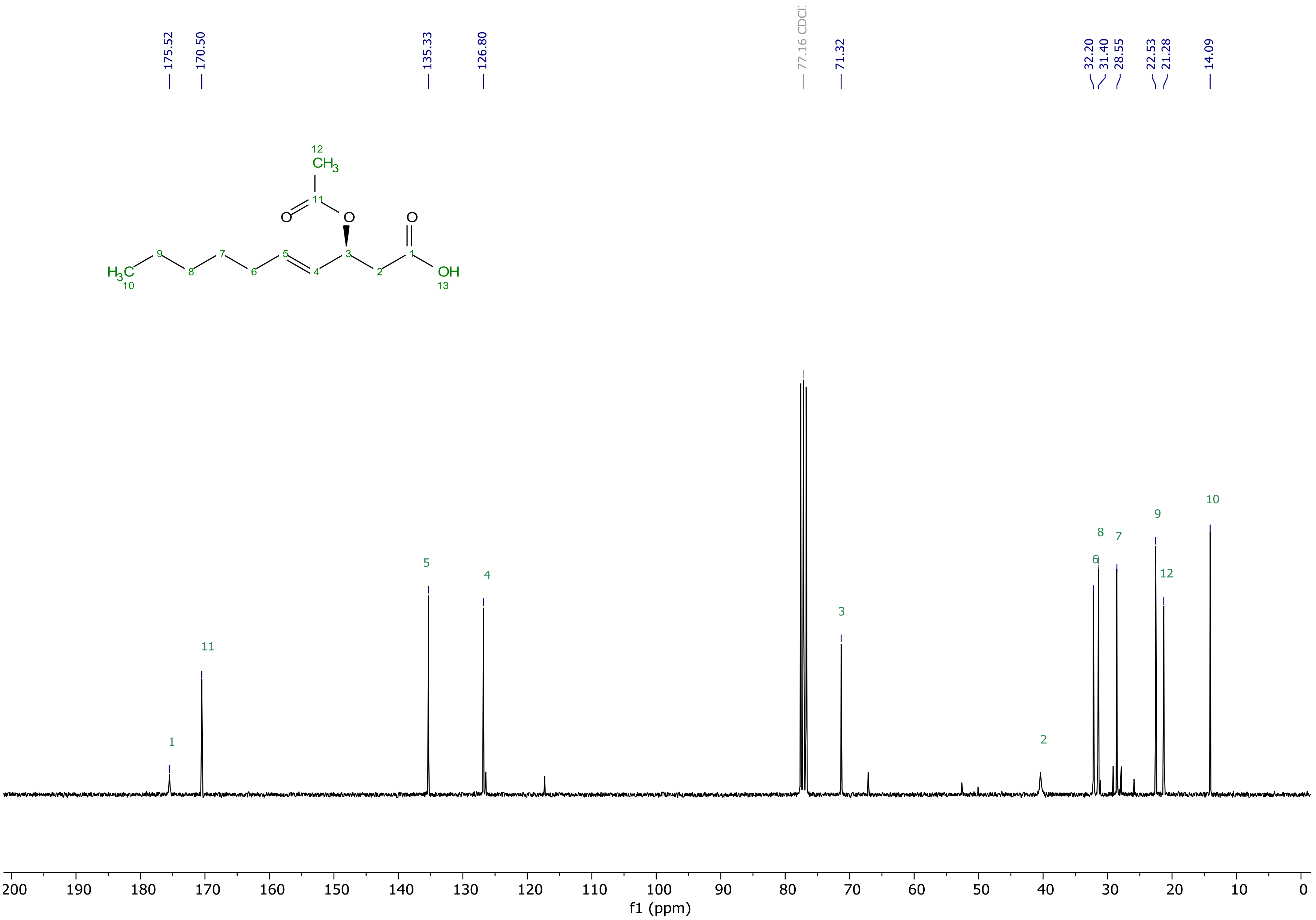

Figure 50: ^{13}C NMR spectrum of compound 6d

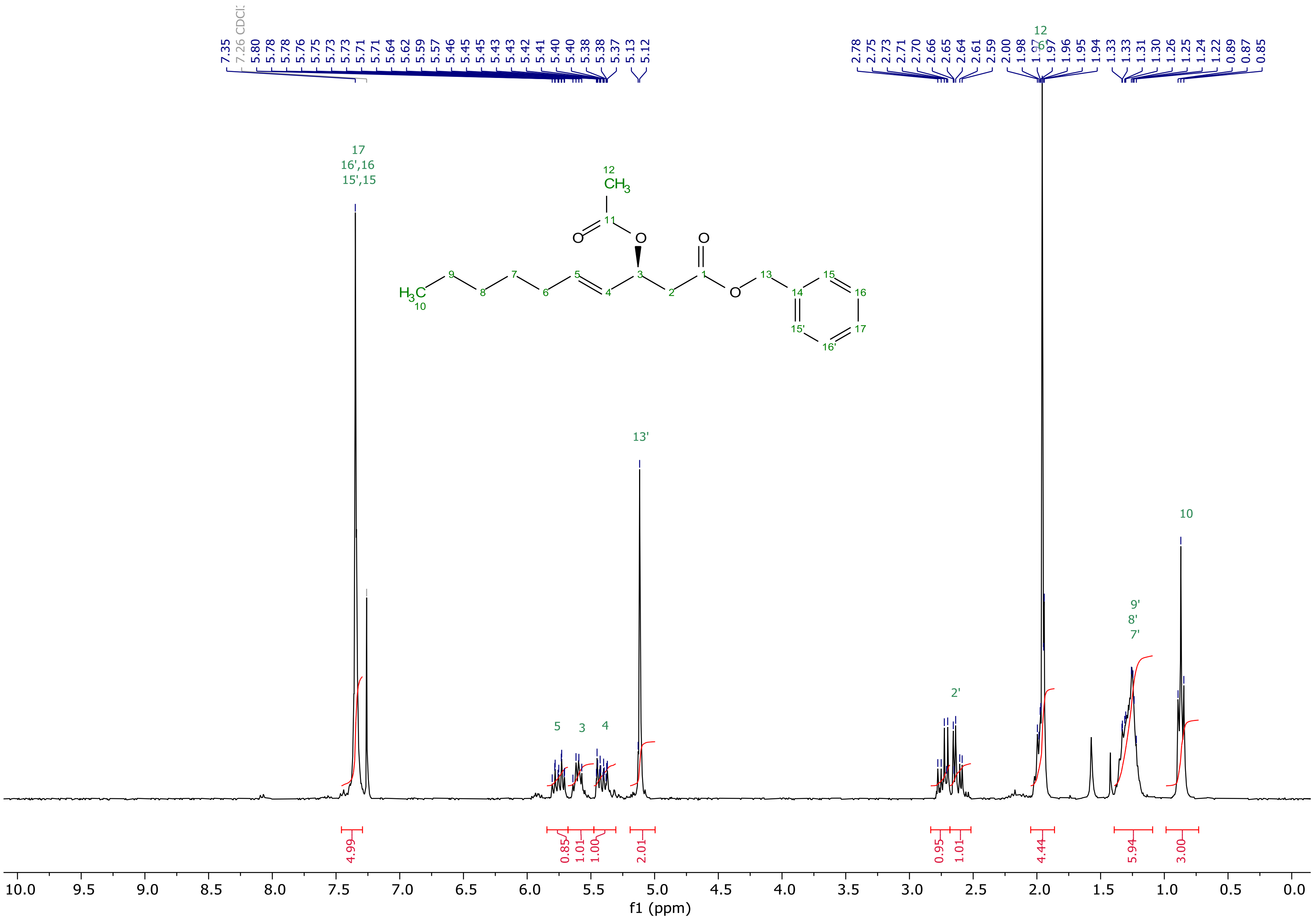

Figure 51: ^{1}H NMR spectrum of compound 8d

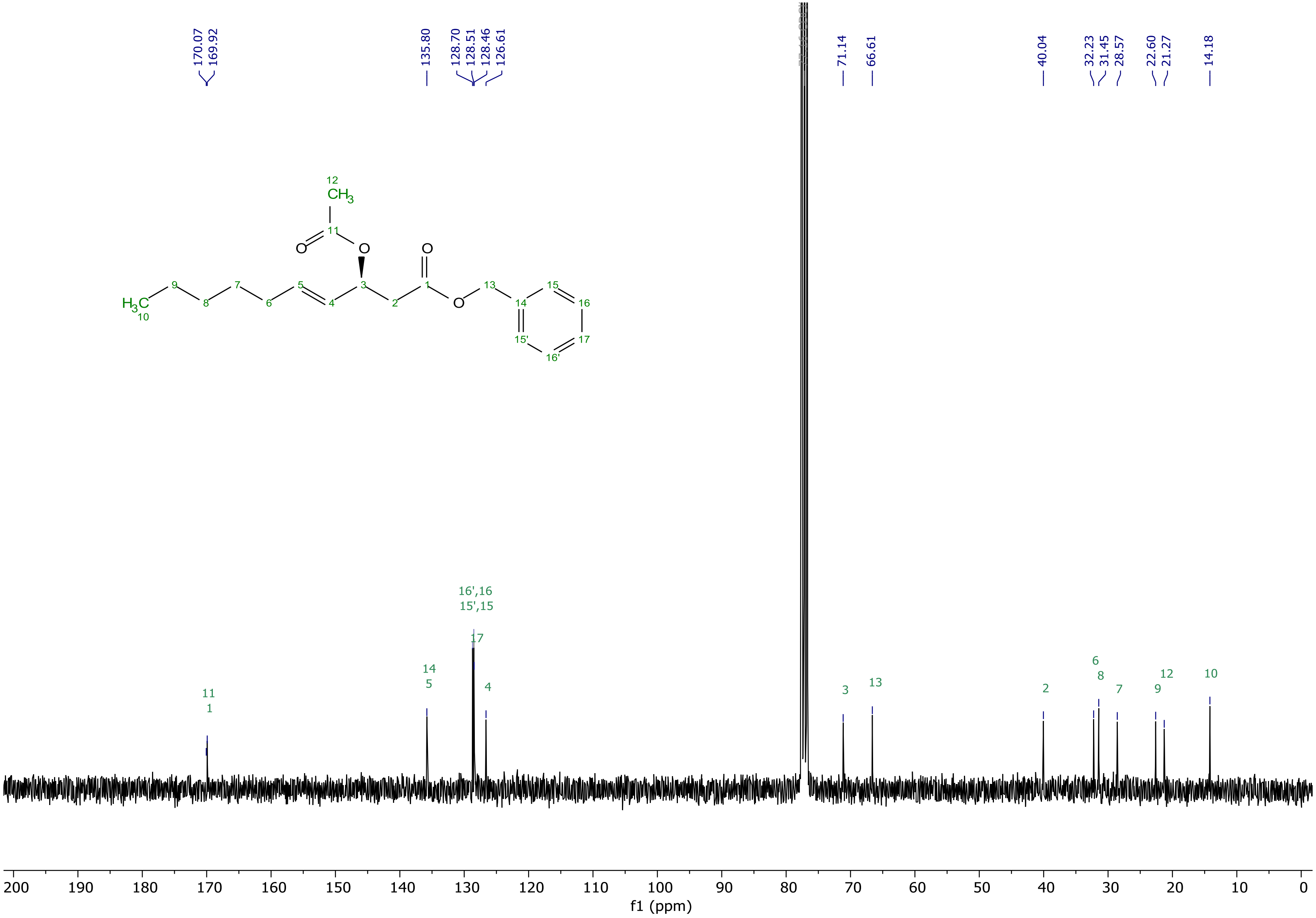

Figure 52: ^{13}C NMR spectrum of compound 8d

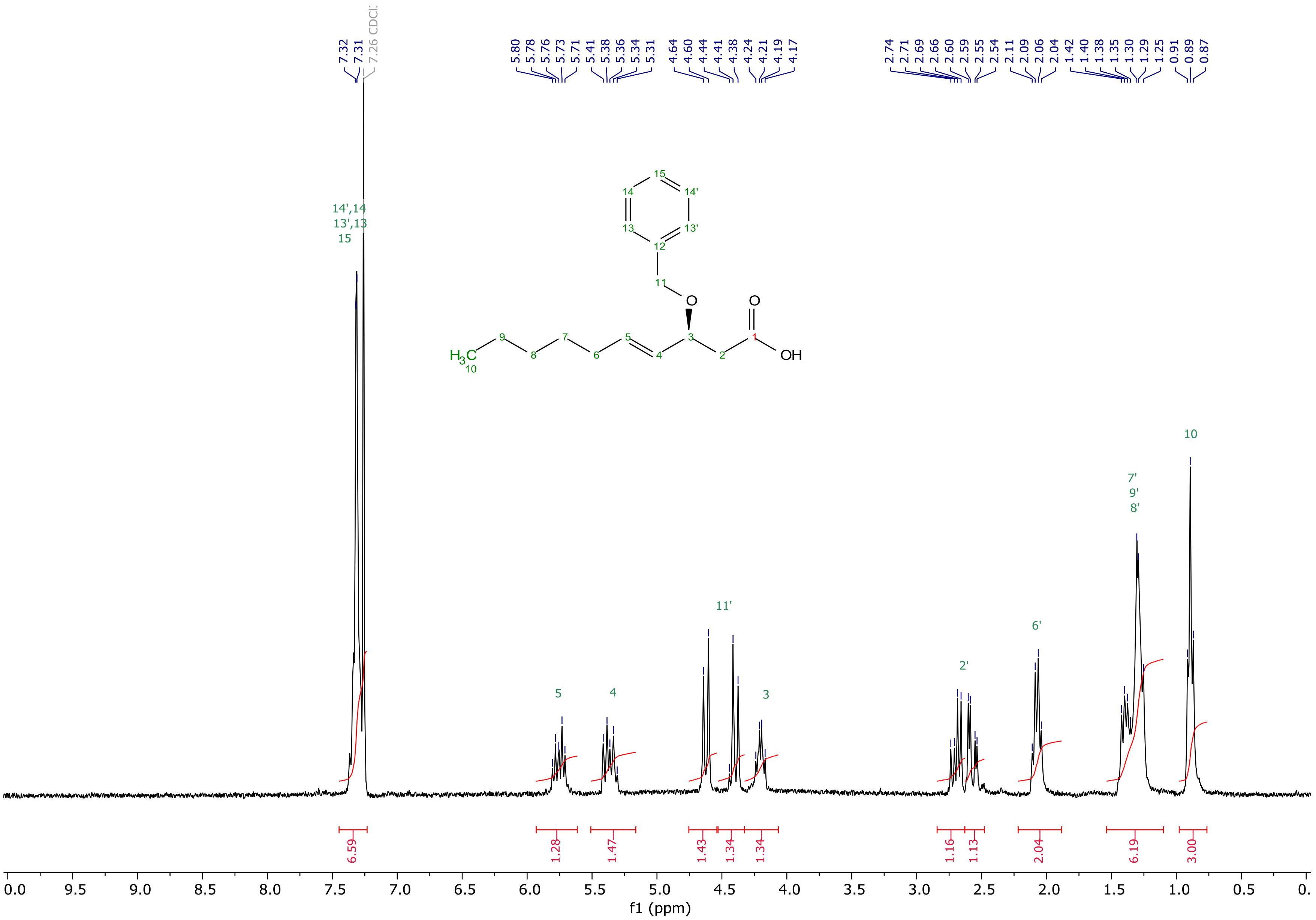

Figure 53: ¹H NMR spectrum of compound 6b

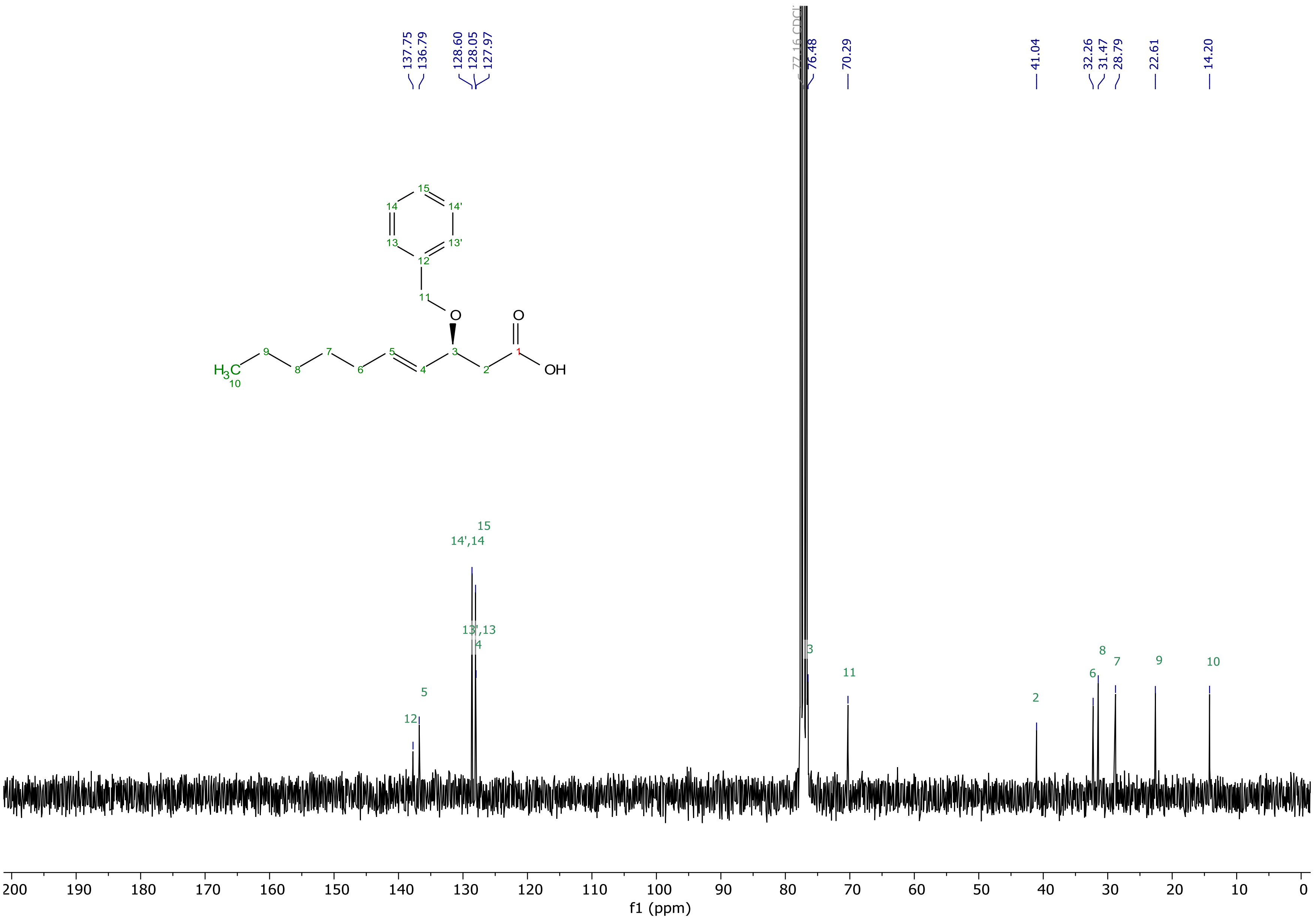

Figure 54: ^{13}C NMR spectrum of compound 6b

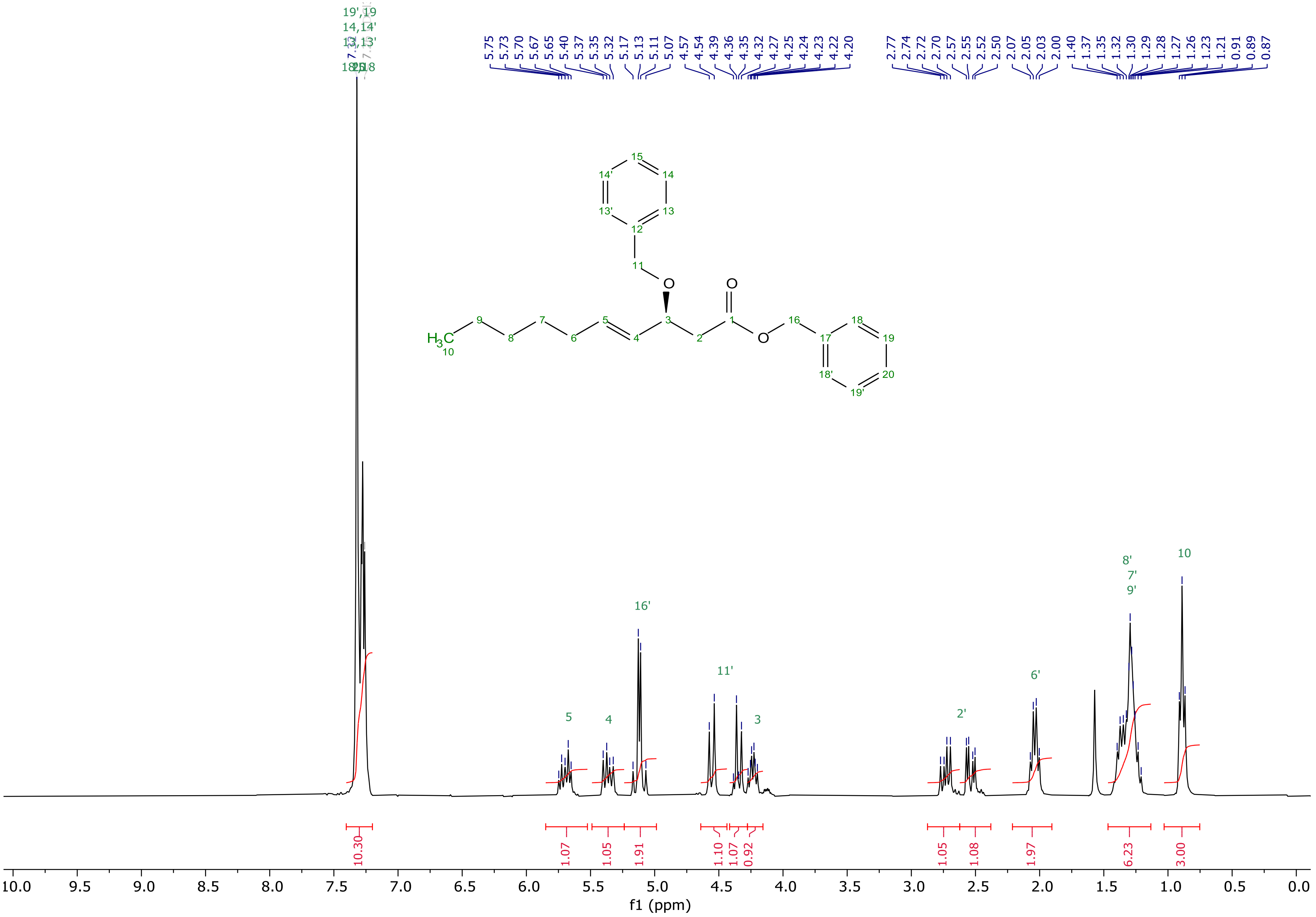

Figure 55: ^{1}H NMR spectrum of compound 8b

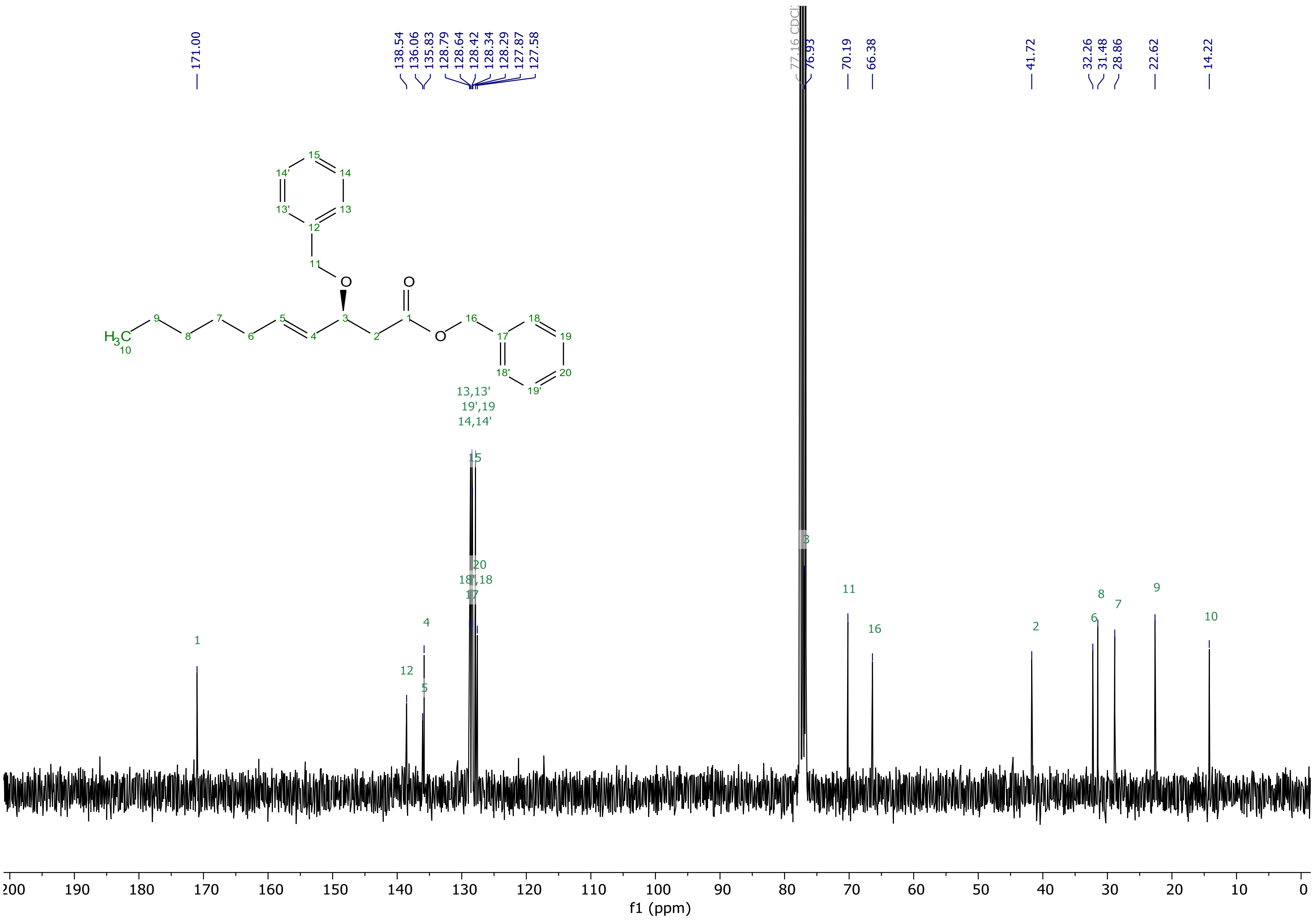

Figure 56: ^{13}C NMR spectrum of compound 8b

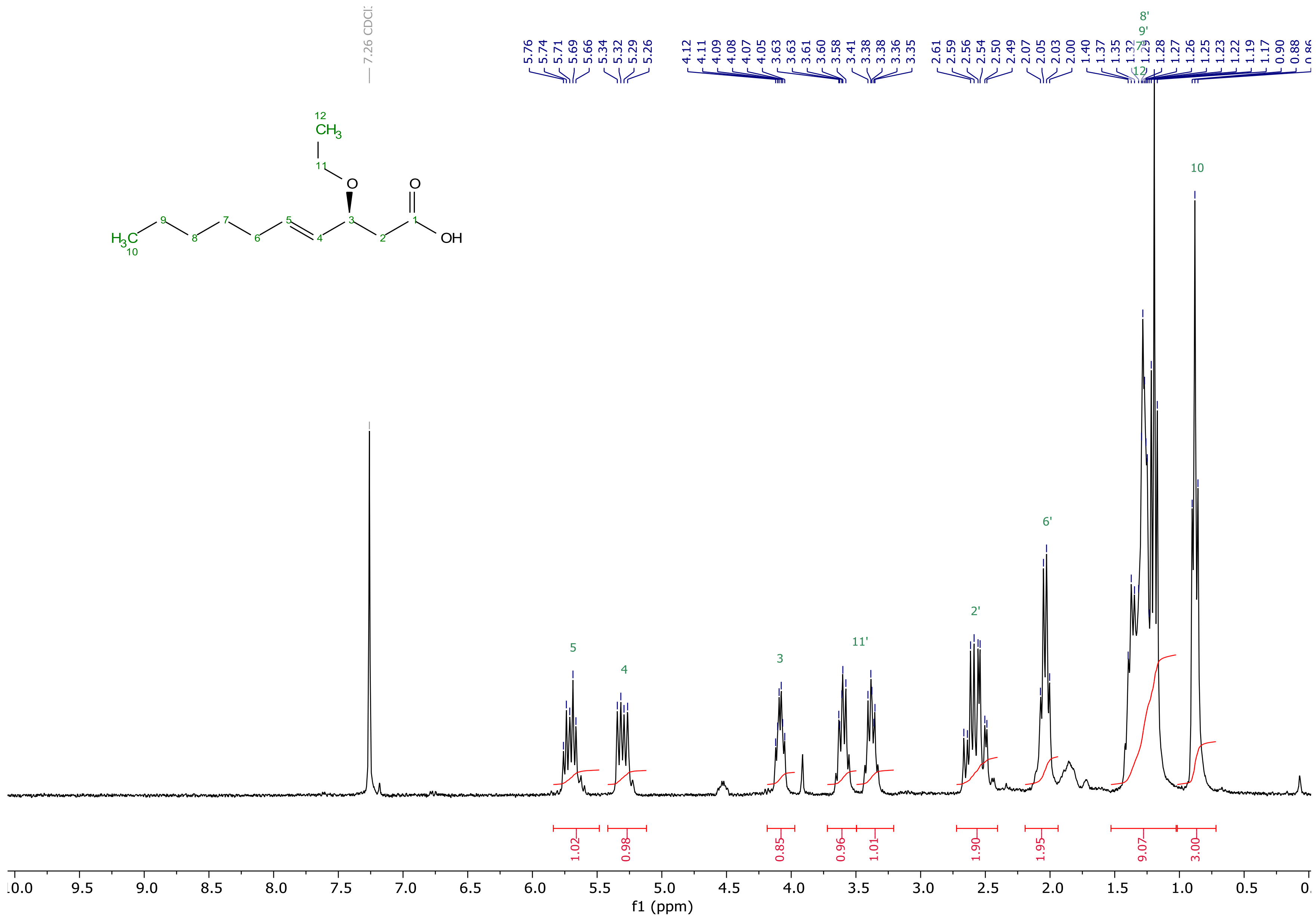

Figure 57: ^{1}H NMR spectrum of compound 6c

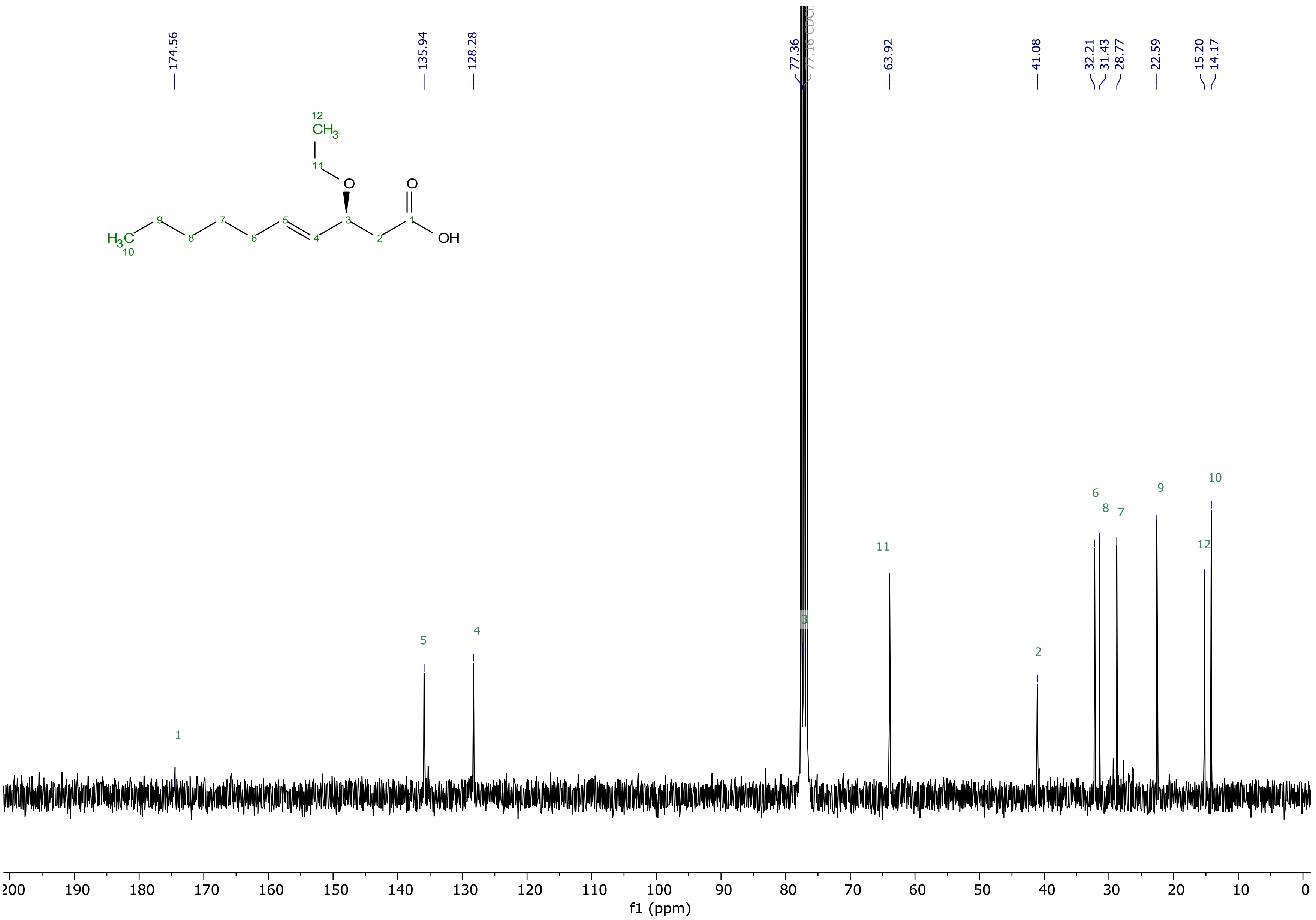

Figure 58: ^{13}C NMR spectrum of compound 6c

2.9. General procedure for hydrogenation

Decenoic acid (or decenoate) derivative was dissolved in ethanol (C= 0.25 M) and the medium was purged under N_2, before addition of palladium on activated charcoal (10% w/w). After another 5 min N_2 purge, the reaction was placed under H_2 flow overnight. Reaction was stopped by replacing H_2 by N_2, then the palladium on charcoal was removed by filtration over PTFE syringe filter which was rinsed with EtOAc. The filtrate was concentrated to dryness.

(*R*)-3-hydroxydecanoic acid 9a, light brown oil (107 mg, 100% yield)

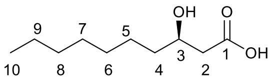

$[\alpha]_D^{22.2}$ = -5.8 (c 1.0, EtOAc) [**$[\alpha]_D^{25}$** = -19.7 (c 1.0, chloroform) lit.[5]]. **^{1}H NMR** ($CDCl_3$, 300 MHz): δ = 4.11-3.91 (m, 1H, H-3), 2.65-2.42 (m, 2H, H-2), 1.64-1.34 (m, 3H, H-5 and H-4a), 1.33-1.14 (m, 9H, H-4b, H-6, H7, H-8 and H-9), 0.94-0.77 (m, 3H, H-10) ppm. **^{13}C NMR** (75 MHz, $CDCl_3$): δ = 177.9 (C-1), 68.1 (C-3), 41.1 (C-2), 36.6 (C-4), 31.9 (C-8), 29.6 (C-6), 29.4 (C-7), 25.6 (C-5), 22.8 (C-9), 14.2 (C-10) ppm. **HRMS** $[M-H]^-$ predicted 187.1339, found 187.1339.

(*R*)-3-acetoxydecanoic acid 9d, colourless oil (76 mg, 95%)

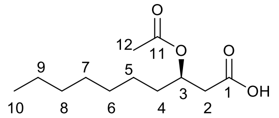

$[\alpha]_D^{24.1}$ = -2.5 (c 1.0, AcOEt) [**$[\alpha]_D^{20}$** = -2.8 (c 0.9, chloroform) lit.[6]]. **^{1}H NMR** ($CDCl_3$, 300 MHz): δ = 5.20 (tt, $J_{3/4}$ = 7.0 Hz and $J_{3/2}$ = 5.6 Hz, 1H, H-3), 2.79-2.51 (m, 2H, H-2), 2.04 (s, 3H, H-12), 1.71-1.54 (m, 2H, H-4), 1.39-1.18 (m, 10H, H-5, H-6, H-7, H-8, H-9), 0.86 (t, $J_{10/9}$=6.6 Hz, 3H, H-10) ppm. **^{13}C NMR** (75 MHz, $CDCl_3$): δ = 176.1 (C-1), 170.7 (C-11), 70.4 (C-3), 38.9 (C-2), 34.1 (C-4), 31.9 (C-8), 29.4 (C-5), 29.3 (C-6), 25.3 (C-7), 22.7 (C-9), 21.3 (C-12), 14.2 (C-10) ppm. **HRMS** $[M+Na]^+$ predicted 253.1410, found 253.1417.

(*R*)-3-(benzyloxy)decanoic acid 9b, black green oil (142 mg, 75%)

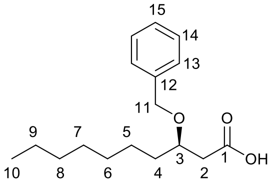

$[\alpha]_D^{24.2}$ = +15.4 (c 0.01, AcOEt). **^{1}H NMR** ($CDCl_3$, 300 MHz): δ = 7.39-7.27 (m, 5H, H-13, H-14, H-15), 5.15 (C-11), 3.92-3.82 (m, 1H, H-3), 2.70-2.50 (m, 2H, H-2), 1.78-1.44 (m, 2H, H-4), 1.41-1.11 (m, 10H, H-5, H-6, H-7, H-8, H-9), 0.94-0.71 (m, 3H, H-10) ppm. **^{13}C NMR** (75 MHz, $CDCl_3$): δ = 173.1 (C-1), 135.7 (C-12), 128.8 (C14), 128.5 (C-13), 128.4 (C-15), 68.2 (C-3), 66.6 (C-11), 41.4 (C-2), 36.6 (C-4), 31.9 (C-8), 29.6 (C-6), 29.4 (C-7), 25.6 (C-5), 22.8 (C-9), 14.2 (C-10) ppm. **HRMS** $[M+Na]^+$ predicted 301.1774, found 301.1784.

(R)-3-ethoxydecanoic acid 9c, grey oil (103 mg, 98% yield)

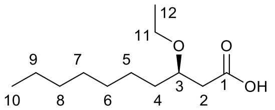

$[\alpha]_D^{23.9}$ = +3.8 (c 0.013, EtOAc). **^{1}H NMR** ($CDCl_3$, 300 MHz): δ = 3.77-3.65 (m, 1H, H-3), 3.64-3.44 (m, 2H, H-11), 1.71-1.43 (m, 2H, H-2), 1.40-1.25 (m, 12H, H-4, H-5, H-6, H-7, H-8, H-9), 1.21 (td, *J* = 7.2, 1.2 Hz, 3H, H-12), 0.92-0.79 (m, 3H, H-10) ppm. **^{13}C NMR** (75 MHz, $CDCl_3$): δ = 175.1 (C-1), 77.4 (C-3), 76.1 (C-11), 65.0 (C-2), 34.2 (C-4), 31.9 (C-8), 29.7 (C-6), 29.4 (C-7), 25.3 (C-5), 22.8 (C-9), 15.6 (C-12), 14.2 (C-10) ppm. **HRMS** $[M-H]^-$ predicted 215.1649, found 215.1649.

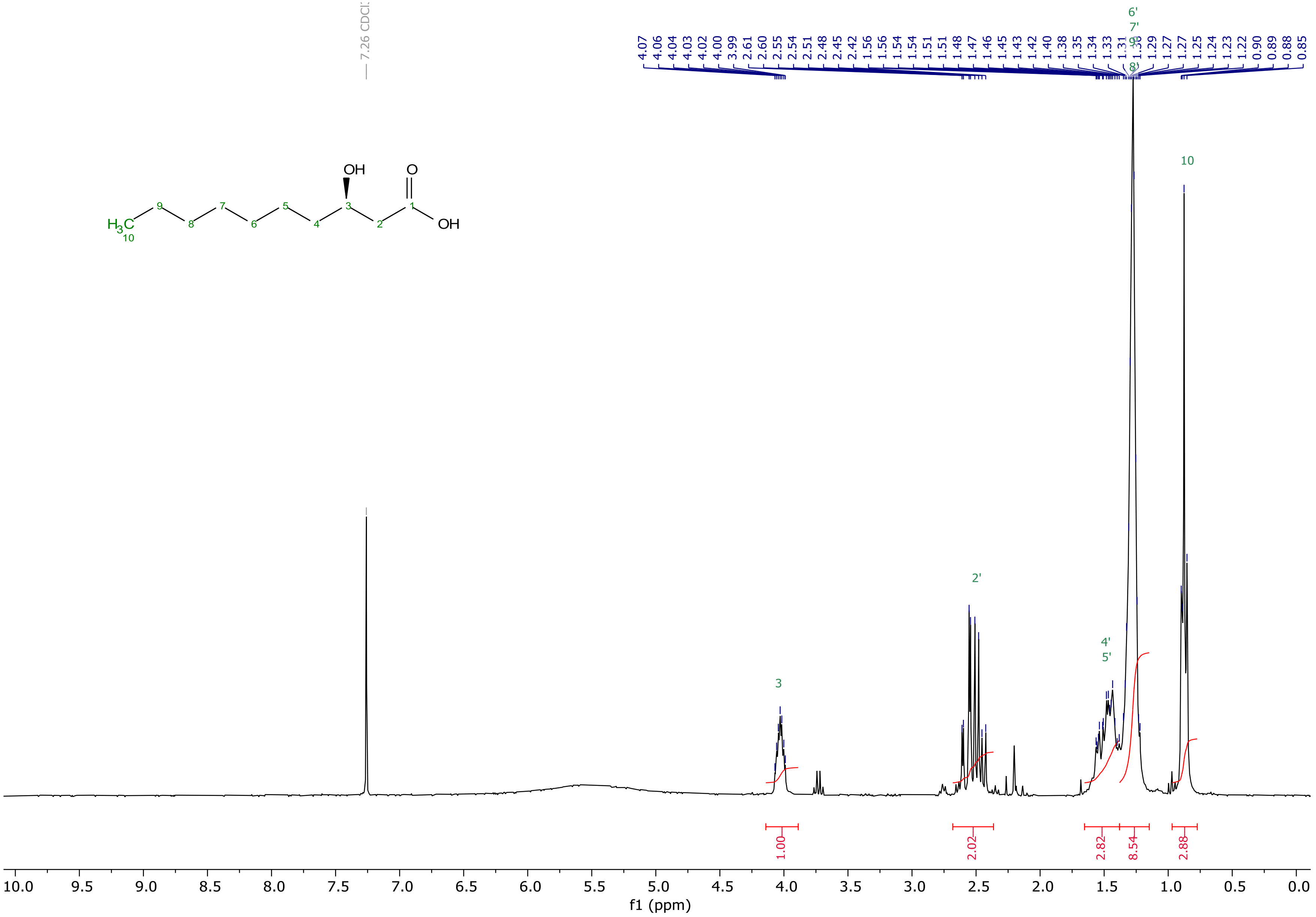

Figure 59: ¹H NMR spectrum of compound 9a

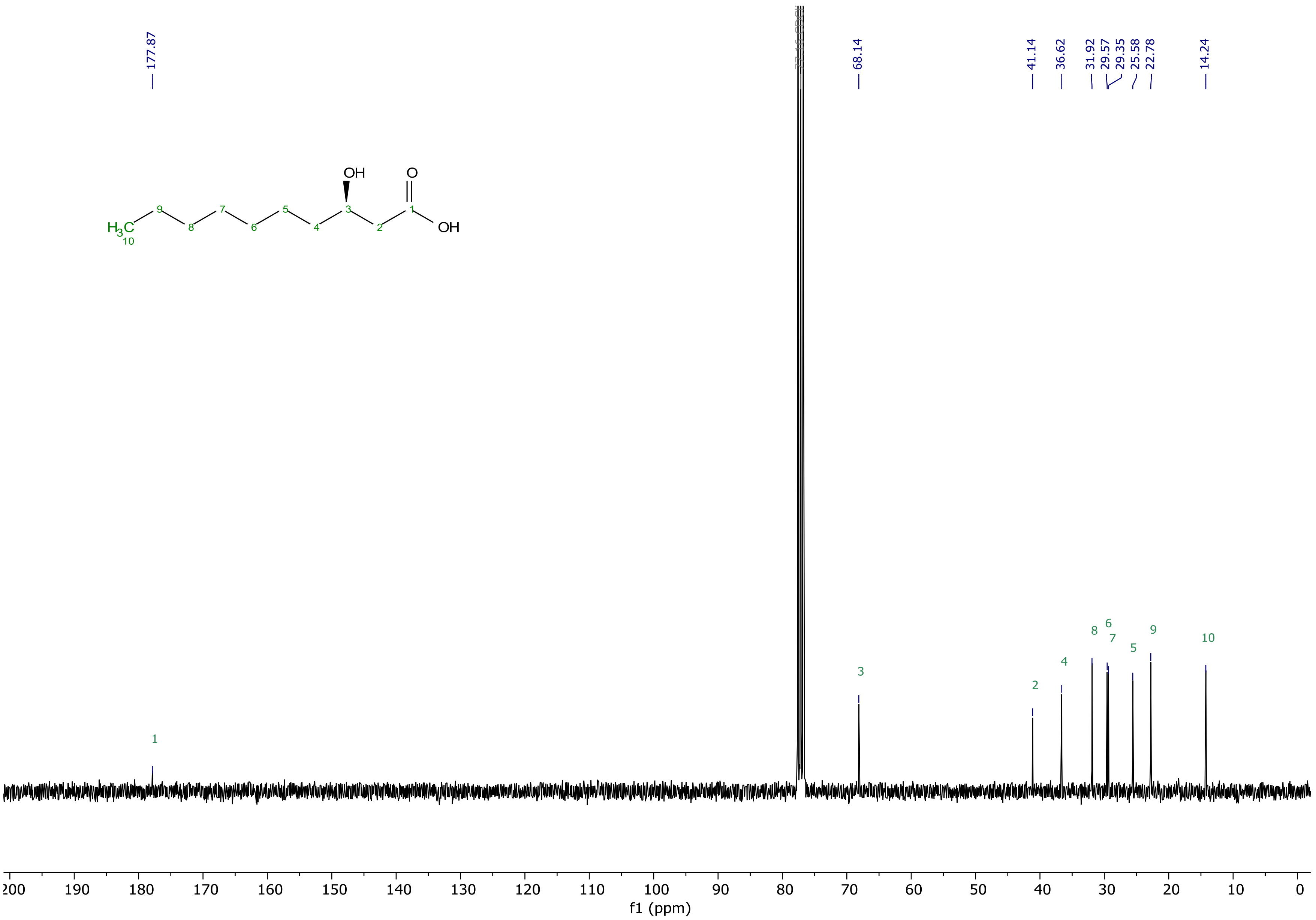

Figure 60: ^{13}C NMR spectrum of compound 9a

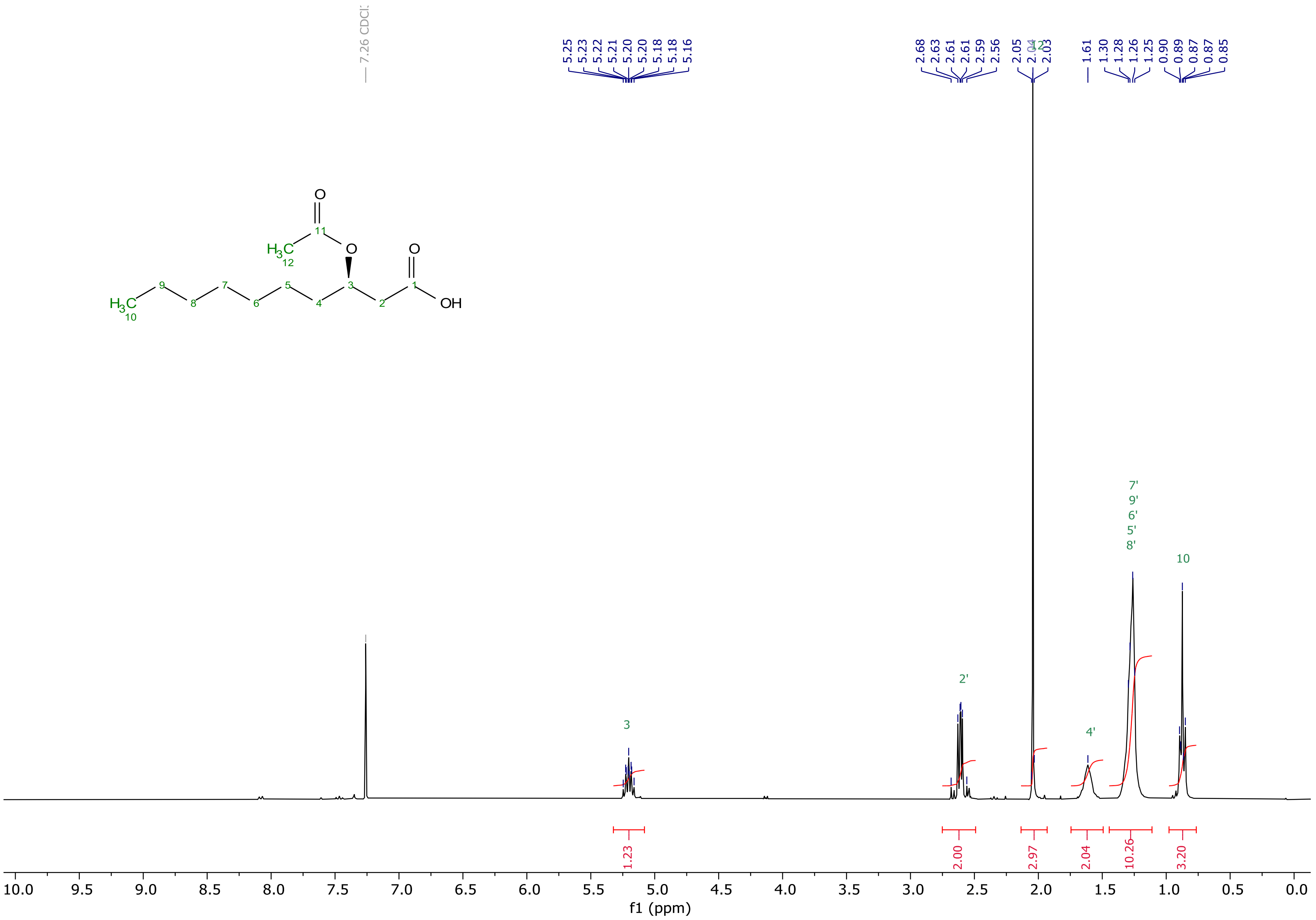

Figure 61: ^{1}H NMR spectrum of compound 9d

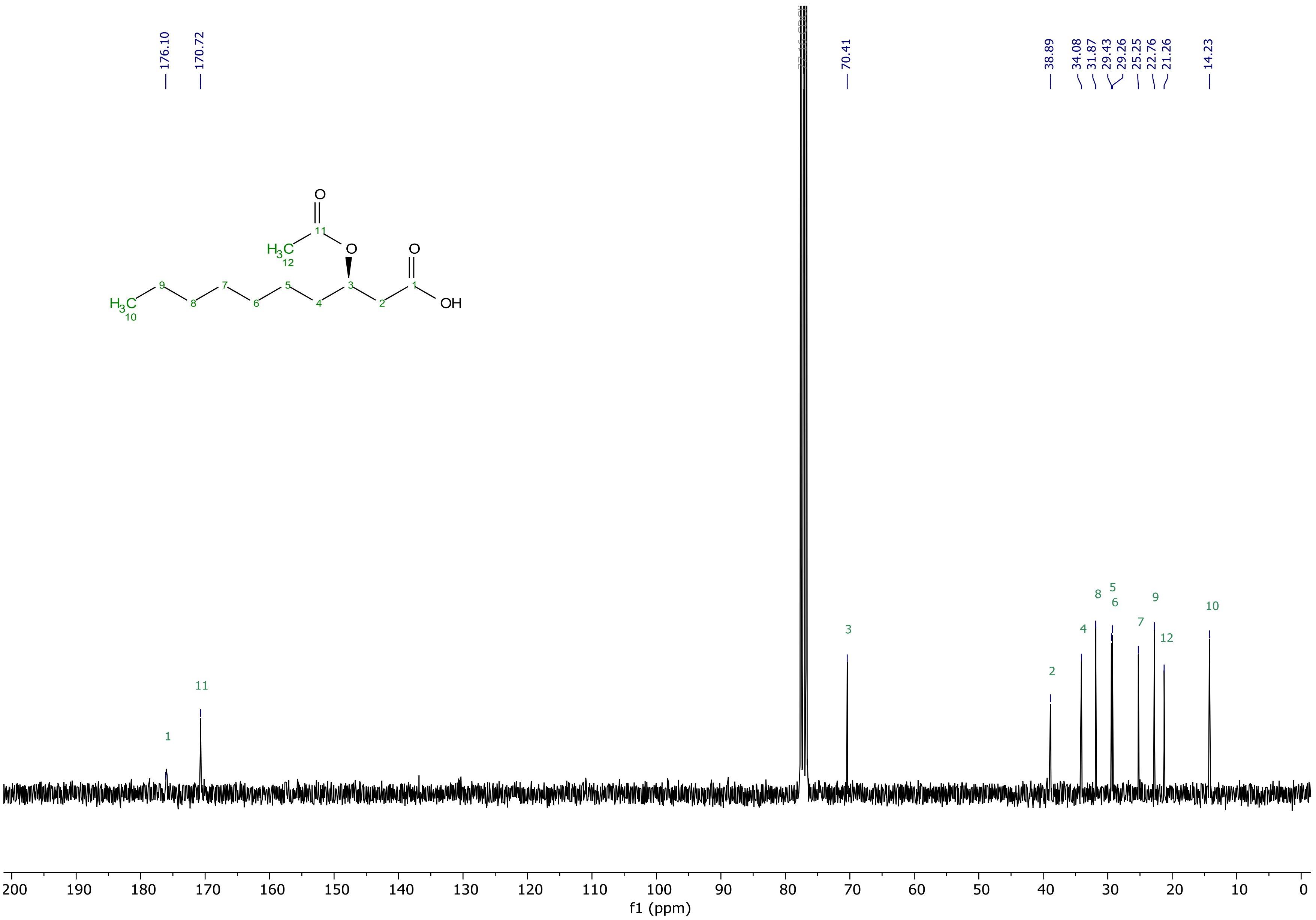

Figure 62: ^{13}C NMR spectrum of compound 9d

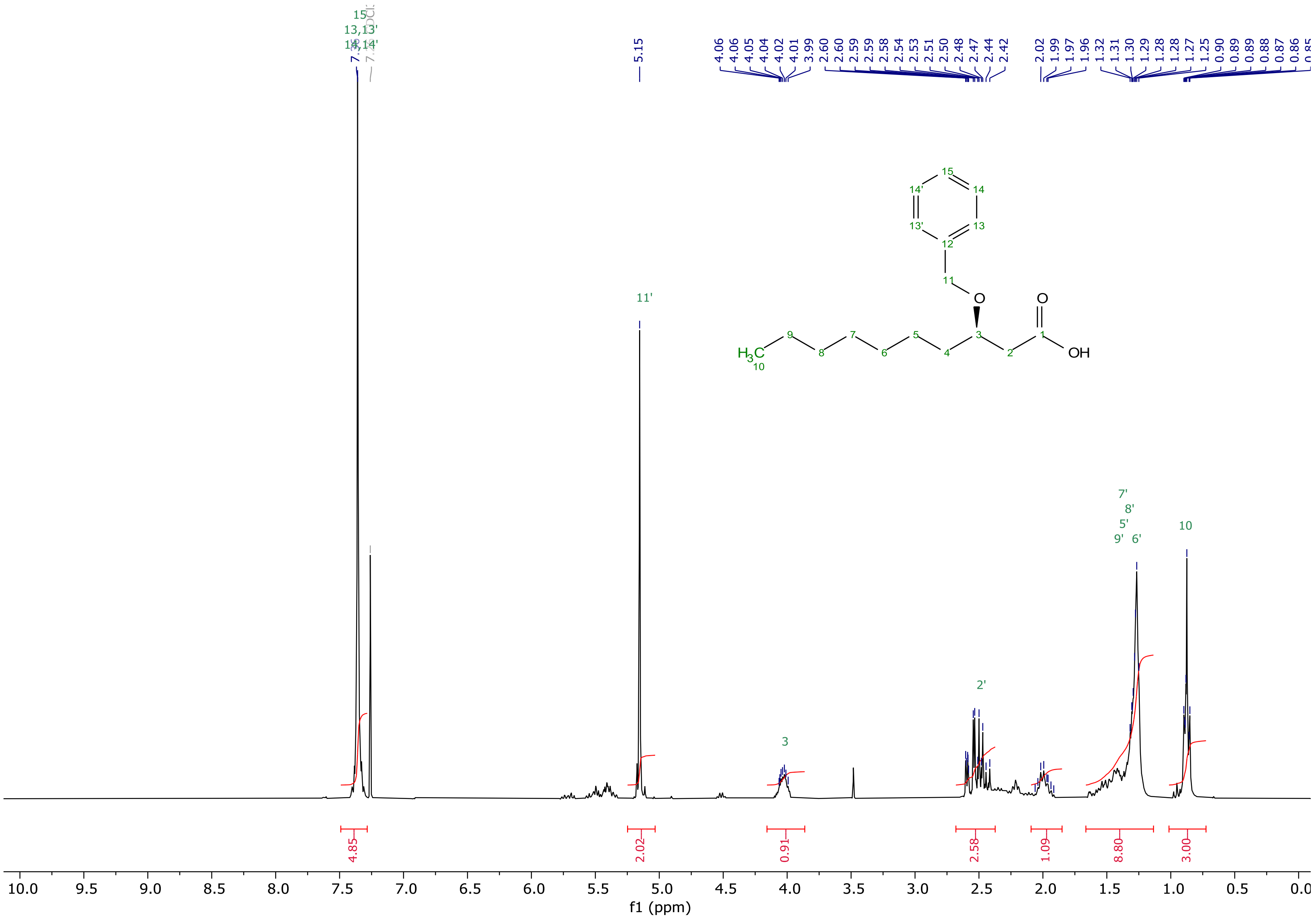

Figure 63: ^{1}H NMR spectrum of compound 9b

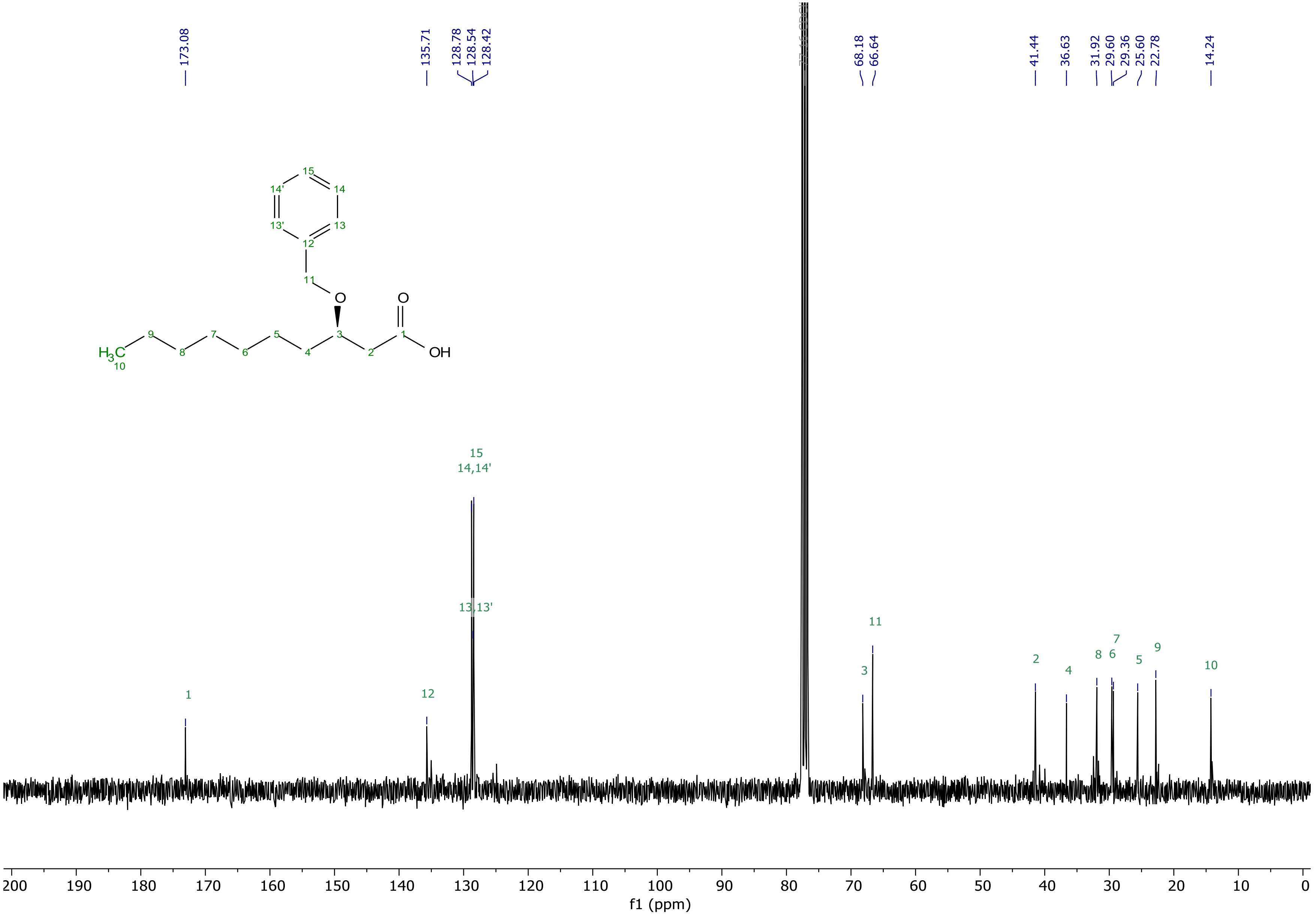

Figure 64: ^{13}C NMR spectrum of compound 9b

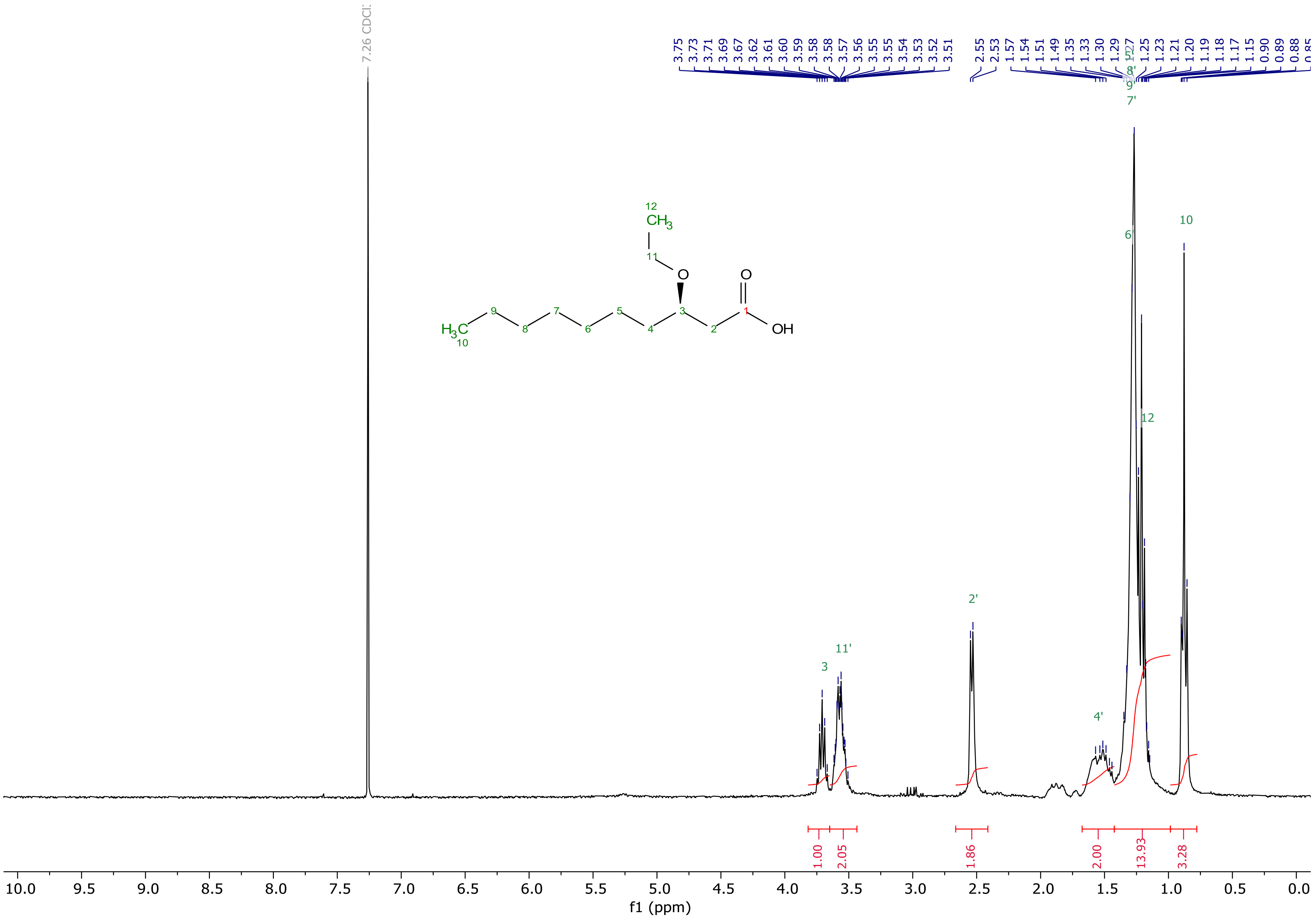

Figure 65: ^{1}H NMR spectrum of compound 9c

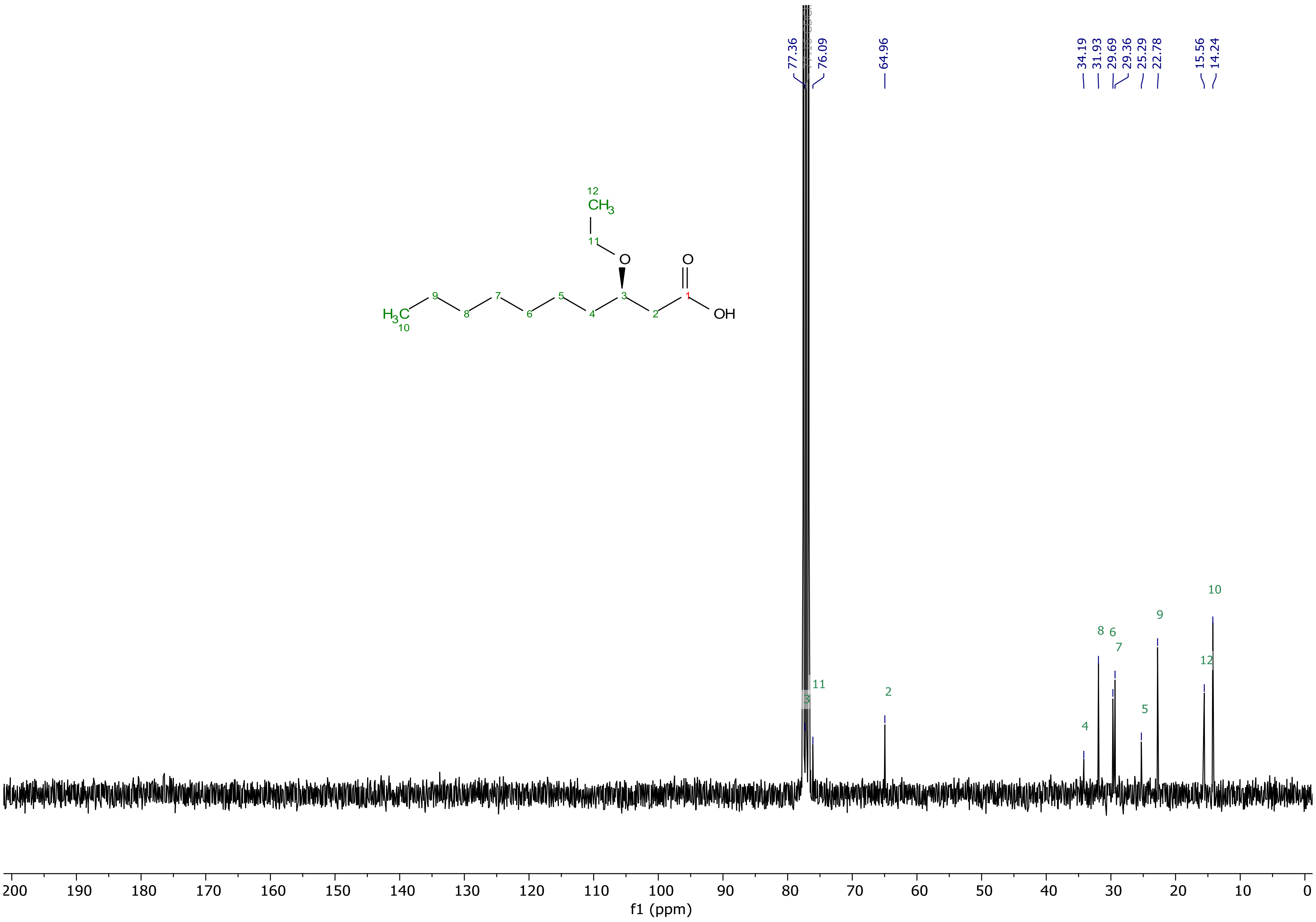

Figure 66: ^{13}C NMR spectrum of compound 9c

3. Design of experiment for optimization of ethanol addition on LGO

A Design of Experiment (DoE) was performed to determine efficient conditions to achieve high yield of levoglucosenone-O-ethyl (LGO-OEt). Modde 12.0 software was used to generate the matrix of the design and analyze the results. Each experiment was conducted on 2 mmol of **LGO** in a 100 mL round bottom flask equipped with a magnetic stirrer (agitation speed 500 rpm) for 72 h.

Table S3.1. Matrix of the design of experiments and experimental results obtained through NMR analysis and given in percentage of product.

Exp Name	Run Order	Incl/Excl	Et_3N	Concentration	LGO	*O*Et-LGO	Dimer
**N1**	7	Incl	0.5	0.08	23.8	74	2.2
**N2**	10	Incl	2	0.08	9.5	86.3	4.2
**N3**	9	Incl	0.5	0.5	13.7	73.1	13.2
**N4**	3	Incl	2	0.5	8	74.4	17.6
**N5**	8	Incl	0.39	0.29	19.3	72.9	7.8
**N6**	6	Incl	2.11	0.29	8.1	79	12.9
**N7**	11	Incl	1.25	0.05	17	81.6	1.4
**N8**	4	Incl	1.25	0.53	6.7	72.9	20.4
**N9**	5	Incl	1.25	0.29	9.8	79	11.2
**N10**	1	Incl	1.25	0.29	8.6	78.4	13
**N11**	2	Incl	1.25	0.29	7.8	78.3	13.9

Figure S3.1. Summary plot for LGO (left), *O*-ethyl Levoglucosenone **2c** (center) and the dimer (right).

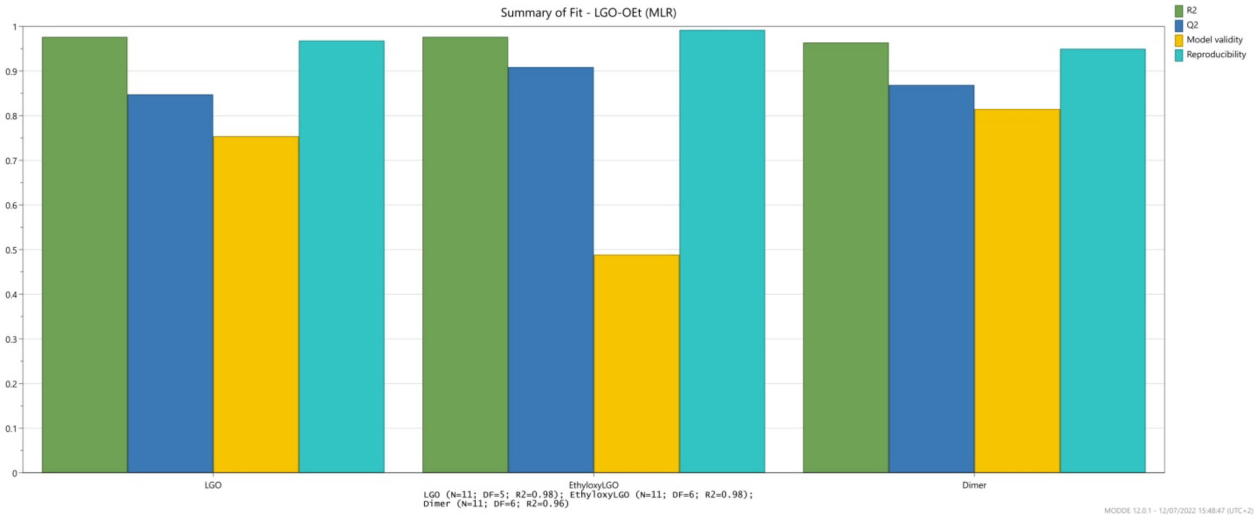

Figure S3.2. Scale and centered coefficients of the model for LGO (left), O-ethyl Levoglucosenone **2c** (center) and the dimer (right).

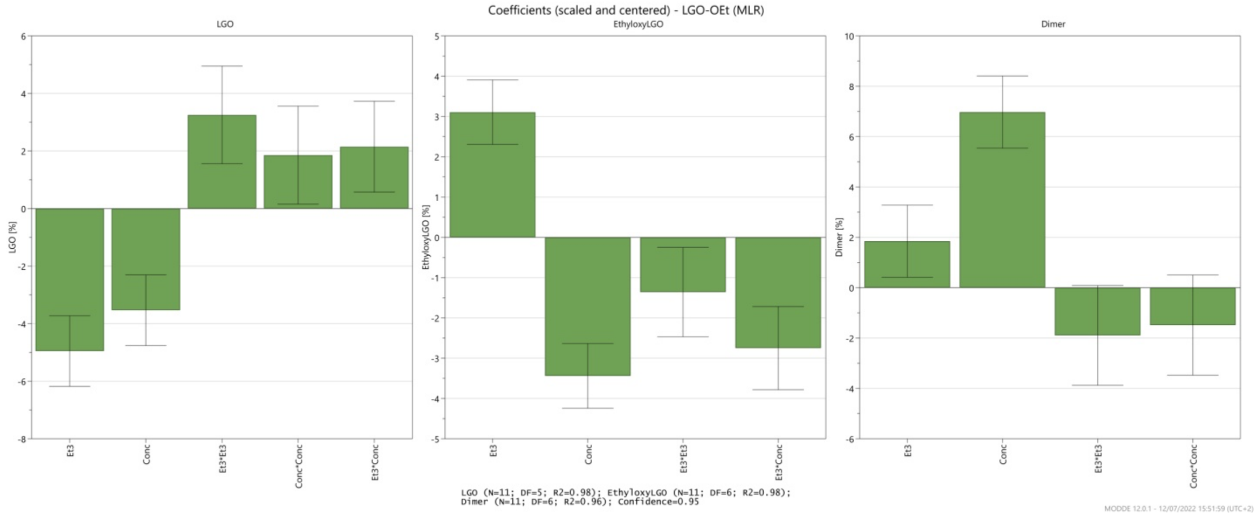

Figure S3.3. Contour plot of LGO (top left), O-ethyl Levoglucosenone **2c** (top right) and the dimer (bottom left).

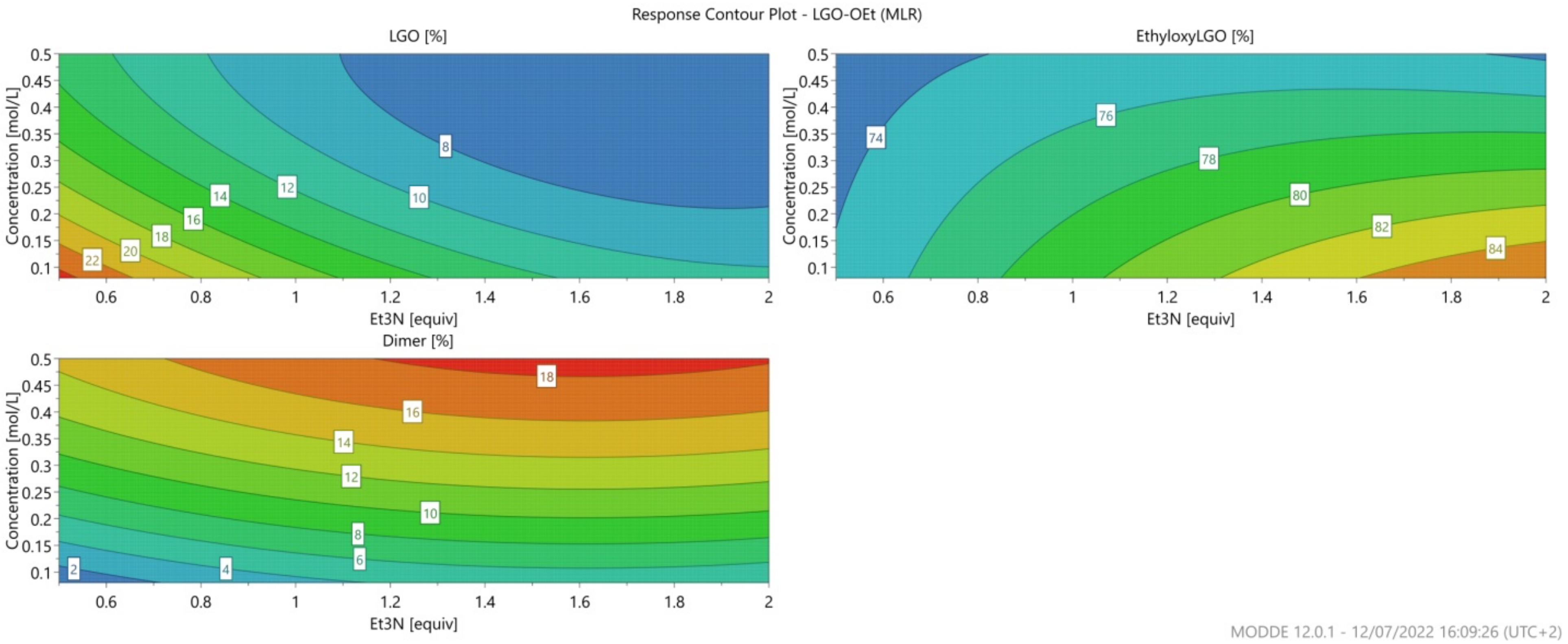

Table S3.2. Experimental validation of the model

LGO	Lower	Upper	Experimental	*O*Et-LGO	Lower	Upper	Experimental	Dimer	Lower	Upper	Experimental
10.5	7.8	13.2	9.0	86.0	84.3	87.7	86.8	4.2	1.7	6.8	4.2%

Table S3.3. ANOVA of the generated models

LGO	DF	SS	MS (variance)	F	p	SD
**Total**	11	1911.21	173.746			
**Constant**	1	1591.21	1591.21			
**Total corrected**	10	320.002	32.0002			5.65687
**Regression**	5	312.454	62.4908	41.3961	**0.000**	7.90511
**Residual**	5	7.5479	1.50958			1.22865
**Lack of Fit**	3	5.52124	1.84041	1.8162	**0.374**	1.35662
**(Model error)**						
**Pure error**	2	2.02667	1.01333			1.00664
**(Replicate error)**						
	N = 11	Q2 =	0.848	Cond. no. =	3.334	
	DF = 5	R2 =	0.976	RSD =	1.229	
		R2 adj. =	0.953			
**EthyloxyLGO**	**DF**	**SS**	**MS (variance)**	**F**	**p**	**SD**
**Total**	11	65849.5	5986.32			
**Constant**	1	65666.4	65666.4			
**Total corrected**	10	183.125	18.3125			4.27932
**Regression**	4	178.855	44.7139	62.8288	**0.000**	6.68684
**Residual**	6	4.27007	0.711678			0.84361
**Lack of Fit**	4	3.9834	0.995851	6.94785	**0.130**	0.997923
**(Model error)**						
**Pure error**	2	0.286665	0.143332			0.378592
**(Replicate error)**						
	N = 11	Q2 =	0.909	Cond. no. =	2.608	
	DF = 6	R2 =	0.977	RSD =	0.8436	
		R2 adj. =	0.961			
**Dimer**	**DF**	**SS**	**MS (variance)**	**F**	**p**	**SD**
**Total**	11	1639.5	149.045			
**Constant**	1	1261.53	1261.53			
**Total corrected**	10	377.969	37.7969			6.14792
**Regression**	4	364.337	91.0841	40.0882	**0.000**	9.5438
**Residual**	6	13.6325	2.27209			1.50735
**Lack of Fit**	4	9.85255	2.46314	1.30325	**0.478**	1.56944
**(Model error)**						
**Pure error**	2	3.78	1.89			1.37477
**(Replicate error)**						
	N = 11	Q2 =	0.869	Cond. no. =	3.334	
	DF = 6	R2 =	0.964	RSD =	1.507	
		R2 adj. =	0.940			

4. Structure elucidation of the unknown compound

To understand how product **10** could be formed, NMRs analysis of **5d** before cross-metathesis has been performed at higher concentration, and traces of the same by-product were detected (Figure S4.1). We assume that **10** was formed during the Bernet-Vasella reaction (Scheme S4.1) but was detected after cross-metathesis because majority of **5d** was consumed during this reaction leading to an increased ratio of the unknown unreacted compound mixed with **5d**. Our hypothesis is that the water, necessary to the Bernet-Vasella reaction, substituted partially the iodine compound, then promoted the rearrangement of the five-membered to a six-membered lactone (Scheme S4.1).

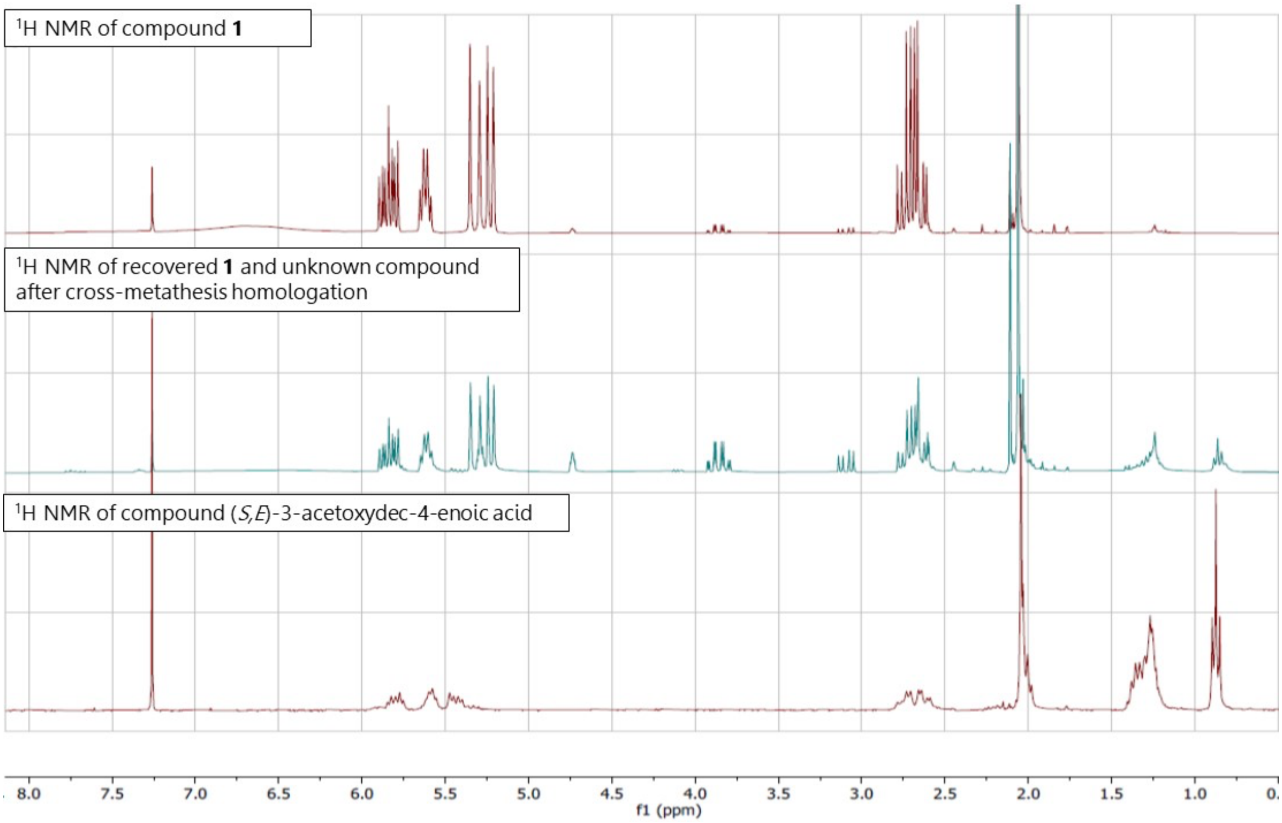

Figure S4.1. Comparison between NMR spectrum of compound **5d** (top), final product (*S,E*)-3-acetoxydec-4-enoic acid (**9d**) (bottom) and the recovered mixture of **5d** and the unknown compound

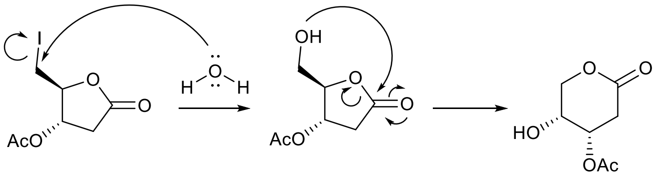

10

Scheme 4.1. Hypothesis for the formation of the unknown compound detected with **5d.**

Table S4.1. NMRs analysis of unknown product hypothesized as **10**

Carbon #	*^{13}C (ppm)*	*^{1}H (ppm)*	*COSY correlation*	*HMBC correlation*
1	*175.1*	*-*	*-*	*-*
2	*44.5*	*3.85*	*H-3*	
3	*83.1*	*4.72*	*H-2*	
4	*35.0*	*3.09+2.62*	*H-5*	
5	*71.8*	*5.30*	*H-4*	*C-1*

5. EcoScale calculations

EcoScale scores were calculated following the methodology described by Van Aken *et al.*[7] and using data available from Bauer *et al.*[8] and Menhour *et al.*[6]

Prices of reagents were taken on the biggest available quantity and for cheaper suppliers between BLD Pharm, TCI, Acros and Sigma Aldrich. Penalty point for Safety was determined from safety data sheet available from Sigma Aldrich website.

5.1. EcoScale of this work

Table S5.1.1. Oxa-Michael addition

Category	Comments	Penalty		
		OH	OBn	OEt
**1. Yield**	75%/94%/86%	12.5	3	7
**2. Price**	No expensive compound	0	0	0
**3. Safety**	K_3PO_4	0	#	#
	Et_3N (F, T+)	#	15	15
	BnOH	#	0	#
	EtOH (F)	#	#	5
**4. Technical setup**	Common setup	0	0	0
**5. Temperature/ Time**	Room temperature >1h	1	1	1
**6. Workup and purification**	Liquid/Liquid Extraction	3	#	#
	Removal solvent bp>150°C	#	2	#
	Removal solvent bp<150°C	0	#	0
	Flash chromatography	0	10	10
**Total**		16.5	31	38
**Score**		**83.5**	**69**	**62**

Table S5.1.2. Baeyer-Villiger oxidation

Category	Comments	Penalty		
		OH	OBn	OEt
**1. Yield**	99%/93%/97%	0.5	3.5	2.5
**2. Price**	No expensive compound	0	0	0
**3. Safety**	H_2O_2 30% in water	0	0	0
	Amberlyst 15 IR	0	0	0
	EtOH (F)	5	5	5
**4. Technical setup**	Instruments for controlled addition of chemicals	1	1	1
**5. Temperature/ Time**	Cooling to 0°C	4	4	4
	Heating >1 h	3	3	3
**6. Workup and purification**	Simple filtration	0	0	0
	Removal solvent bp<150°C	0	0	0
**Total**		13.5	16.5	15.5
**Score**		**86.5**	**83.5**	**84.5**

Table S5.1.3. Activation

Category	Comments	Penalty			
		OH	OAc	OBn	OEt
**1. Yield**	77%/77%/88%/74%	11.5	11.5	6	13
**2. Price**	No expensive compound	0		0	0
**3. Safety**	TsCl	0	0	#	0
	Ac_2O (F, T+)	#	15	#	#
	MsCl (T+)	#	#	10	#
	Pyridine (F)	5	5	5	5
**4. Technical setup**	Common setup	0	0	0	0
**5. Temperature/ Time**	r.t. <24 h	1	1	1	1
**6. Workup and purification**	Liquid/Liquid extraction	3	3	3	3
	Removal solvent bp<150°C	0	0	0	0
**Total**		20.5	35.5	25	22
**Score**		**79.5**	**64.5**	**75**	**78**

Table S5.1.4. Iodination

Category	Comments	Penalty			
		OH	OAc	OBn	OEt
**1. Yield**	100%	0	0	0	0
**2. Price**	No expensive compound	0	0	0	0
**3. Safety**	NaI (N, T+)	15	15	15	15
	Acetone (F)	5	5	5	5
**4. Technical setup**	Common setup	0	0	0	0
**5. Temperature/ Time**	Heating>1h	3	3	3	3
**6. Workup and purification**	None				
**Total**		23	23	23	23
**Score**		**77**	**77**	**77**	**77**

Table S5.1.5. Bernet-Vasella reaction

Category	Comments	Penalty			
		OH	OAc	OBn	OEt
**1. Yield**	55%/89%/95%/78%	22.5	5.5	2.5	11
**2. Price**	No expensive compound	0	0	0	0
**3. Safety**	Zinc	0	0	0	0
	Water	0	0	0	0
**4. Technical setup**	Common setup	0	0	0	0
**5. Temperature/ Time**	Heating<1h	2	2	2	2
**6. Workup and purification**	Simple filtration	0	0	0	0
	L/L extraction	3	3	3	3
	Flash chromatography	10	10	0	10
**Total**		37.5	20.5	7.5	26
**Score**		**62.5**	**79.5**	**92.5**	**74**

Table S5.1.6. Benzylation

Category	Comments	Penalty		
		OH	OAc	OBn
**1. Yield**	84%/82%/85%	8	9	7.5
**2. Price**	No expensive compound	0	0	0
**3. Safety**	K_2CO_3	0	0	0
	BnBr	0	0	0
	DMF (F, T+)	15	15	15
**4. Technical setup**	Inert atmosphere	1	1	1
**5. Temperature/ Time**	r.t. 3 h	1	1	1
**6. Workup and purification**	Simple filtration	0	0	0
	DMF removal	2	2	2
	L/L extraction	3	3	3
**Total**		30	31	29.5
**Score**		**70**	**69**	**70.5**

Table S5.1.7. Cross-metathesis homologation on carboxylic acid compounds

Category	Comments	Penalty			
		OH	OAc	OBn	OEt
**1. Yield**	46%/62%/47%/60%	27	19	26.5	20
**2. Price**	GII[a]	5	3	5	3
**3. Safety**	GII	0	0	0	0
	CuI (N)	5	5	5	5
	Hept-1-ene (F, T+)	15	15	15	15
	CPME (F)	5	5	5	5
**4. Technical setup**	Inert atmosphere	1	1	1	1
	Instruments for controlled addition of chemicals	1	1	1	1
**5. Temperature/ Time**	Heating>1h	3	3	3	3
**6. Workup and purification**	Simple filtration	0	0	0	0
	Flash chromatography	10	10	10	10
**Total**		72	62	71.5	63
**Score**		**28**	**38**	**28.5**	**37**

Table S5.1.8. Cross-metathesis homologation on benzylated compounds

Category	Comments	Penalty	
		OH	OAc
**1. Yield**	59%/38%	20.5	31
**2. Price**	GII[a]	5	5
**3. Safety**	GII	0	0
	CuI (N)	5	5
	Hept-1-ene (F, T+)	15	15
	CPME (F)	5	5
**4. Technical setup**	Inert atmosphere	1	1
	Instruments for controlled addition of chemicals	1	1
**5. Temperature/ Time**	Heating>1h	3	3
**6. Workup and purification**	Simple filtration	0	0
	Flash chromatography	10	10
**Total**		65.5	76
**Score**		**34.5**	**24**

Table S5.1.9. Hydrogenation

Category	Comments	Penalty			
		OH	OAc	OBn	OEt
**1. Yield**	100%/95%/75%/98%	0	2.5	12.5	1
**2. Price**	Pd/C <3€	0	0	0	0
**3. Safety**	Pd/C	0	0	0	0
	H_2 (F)	5	5	5	5
	EtOH (F)	5	5	5	5
**4. Technical setup**	Gas atmosphere	1	1	1	1
**5. Temperature/ Time**	r.t.<24 h	1	1	1	1
**6. Workup and purification**	Simple filtration	0	0	0	0
	Concentrated	0	0	0	0
	Flash chromatography	#	#	10	#
**Total**		12	14.5	34.5	13
**Score**		**88**	**85.5**	**65.5**	**87**

Similar results were obtained from both carboxylic acid or benzylated compounds.

Average score for (*R*)-3-hydroxydecanoic acid 72.1 (from carboxylic acid route) or 64.9 (from benzylation route)

Average score for (*R*)-3-acetoxydecanoic acid 73.5 (from carboxylic acid route) or 71.2 (from benzylation route)

Average score for (*R*)-3-benzyloxydecanoic acid 70.1

Average score for (*R*)-3-ethoxydecanoic acid 71.4

5.2. EcoScale of Bauer's procedure

Table S5.2.1. EcoScale of Bauer's procedure (first step)

Category	Comments	Penalty
**1. Yield**	78%	11
**2. Price**	No expensive compound	0
**3. Safety**	Meldrum's acid (N)	5
	Pyridine (F)	5
	CH_2Cl_2 (T+)	10
	Octanoyl chloride (T+)	10
**4. Technical setup**	addition funnel	1
**5. Temperature/Time**	1 h at 0°C then 2h at r.t.	4
**6. Workup and purification**	Washing HCl (x3), water	3
	Dry over $MgSO_4$	0
	Concentrated	0
**Total**		49

Score		51

Table S5.2.2. EcoScale of Bauer‘s procedure (second step)

Category	Comments	Penalty
1. Yield	100%	0
2. Price	No expensive compound	0
3. Safety	Methanol (F, T+)	15
4. Technical setup	Common setup	0
5. Temperature/Time	Reflux, 3 h	3
6. Workup and purification	Concentration	0
	Classical chromatography	10
Total		28
Score		72

Table S5.2.3. EcoScale of Bauer‘s procedure (third step)

Category	Comments	Penalty
1. Yield	96%	2
2. Price	(R)-Binap (0.95€)	0
	$[RuCl_2(benzene)]_2$ (1.71€)	0
3. Safety	Methanol (F, T+)	15
	(R)-Binap	0
	$[RuCl_2(benzene)]_2$	0
	DMF (F, T+)	15
	H_2 (F)	5
4. Technical setup	Gas atmosphere	1
	Schlenk line	1
	Pressure equipment (5 bars H_2)	3
5. Temperature/Time	100 C, 5 h	3
6. Workup and purification	Concentration	0
	Classical chromatography	10
Total		55
Score		45

Table S5.2.4. EcoScale of Bauer‘s procedure (fourth step)

Category	Comments	Penalty
1. Yield	98%	1
2. Price	No expensive compound	0
3. Safety	Methanol (F, HT)	15
	$LiOH.H_2O$ (T+)	10
	CH_2Cl_2 (T+)	10
4. Technical setup	Common setup	0
5. Temperature/Time	Room temperature 12h	1
6. Workup and purification	Acidification	0
	L/L Extraction	3
	Concentration	0
Total		40
Score		60

Table S5.2.5. EcoScale of Bauer‘s procedure (fifth step)

Category	Comments	Penalty
1. Yield	86%	7
2. Price	Triethylsilyl chloride (1€)	0
3. Safety	Pyridine (F)	5
	Triethylsilyl chloride (F, T+)	15
	$CHCl_3$ (N, T+)	15
4. Technical setup	Controlled addition	1
5. Temperature/Time	60 C, 2h	3
6. Workup and purification	Concentration	0
	L/L Extraction	3
	Classical chromatography	10
Total		59
Score		41

Table S5.2.6. EcoScale of Bauer's procedure (sixth step)

Category	Comments	Penalty
**1. Yield**	81%	9,5
**2. Price**	EDC (10.5€)	3
**3. Safety**	Benzyl alcohol	0
	EDC (N, T+)	15
	CH_2Cl_2 (T+)	10
	DMAP (N, T+)	15
**4. Technical setup**	Common setup	0
**5. Temperature/Time**	Room temperature 12h	1
**6. Workup and purification**	Concentration	0
	Classical chromatography	10
**Total**		63,5
**Score**		**36,5**

Table S5.2.7. EcoScale of Bauer's procedure (seventh step)

Category	Comments	Penalty
**1. Yield**	98%	1
**2. Price**	No expensive compound	0
**3.Safety**	TFA (N)	5
	CH_2Cl_2 (T+)	10
**4. Technical setup**	Common setup	0
**5. Temperature/Time**	Room temperature 30 min	0
**6. Workup and purification**	L/L Extraction	3
	Concentration	0
	Classical chromatography	10
**Total**		29
**Score**		**71**

Average score: 54, acceptable procedure

5.3. EcoScale of Menhour's procedure

Table S5.3.1. EcoScale of Menhour's procedure (first step)

Category	Comments	Penalty
**1. Yield**	100%	0
**2. Price**	Acrolein (not available)	0
	No expensive compound	0
**3. Safety**	Acrolein (N, F, T+)	20
	*t*Bu acetate (F)	5
	*n*BuLi (F, T+)	15
	Diisopropylamine (F, T+)	15
	THF (F)	5
	Et_2O (F+, T)	15
**4. Technical setup**	Dropwise addition for *n*BuLi	1
	Inert atmosphere	1
**5. Temperature/Time**	-78 C, >1h	5
**6. Workup and purification**	Distillation of acrolein	3
	Quench with NH_4Cl	0
	Extract with Et_2O	1
	Dry over $MgSO_4$	0
	Concentrated	0
**Total**		86
**Score**		**14**

Table S5.3.2. EcoScale of Menhour's procedure (second step)

Category	Comments	Penalty
**1. Yield**	42%	29
**2. Price**	PS amano Lipase (11.9€)	3
**3. Safety**	PS amano Lipase	0

	Vinyl acetate (F, T+)	15
	Pentane (N, F, T+)	20
	Et_2O (F+, T)	15
**4. Technical setup**	Common setup	0
**5. Temperature/Time**	30 °C for 16 h	3
**6. Workup and purification**	Filtration	0
	Washed with Et_2O	0
	Concentration	0
	Classical chromatography	10
**Total**		95
**Score**		**5**

Table S5.3.3. EcoScale of Menhour's procedure (third step)

Category	Comments	Penalty
**1. Yield**	79%	10,5
**2. Price**	Grubbs II cat (15.6€)<br>Hept-1-ene (9.4€)	3
**3. Safety**	Grubbs II cat	0
	Copper iodide (N)	5
	Hept-1-ene (F, T+)	15
	Et_2O (F+, T)	15
**4. Technical setup**	Inert atmosphere	1
**5. Temperature/Time**	40 °C for 1h30	3
**6. Workup and purification**	Filtration	0
	Washed with Et_2O	0
	Concentration	0
**Total**		52,5
**Score**		**47.5**

Table S5.3.4. EcoScale of Menhour's procedure (fourth step)

Category	Comments	Penalty
**1. Yield**	100%	0
**2. Price**	PtO_2 (2,9€ estimated)	0
**3. Safety**	PtO_2	0
	EtOAc (F)	5
	H_2 (F)	5
**4. Technical setup**	Gas atmosphere	1
**5. Temperature/Time**	Room temperature 2h	1
**6. Workup and purification**	Filtration	0
	Concentration	0
	Flash chromatography	10
**Total**		22
**Score**		**78**

Table 5.3.5. EcoScale of Menhour's procedure fifth step

Category	Comments	Penalty
**1. Yield**	89%	5,5
**2. Price**	No expensive compound	0
**3. Safety**	TFA (N)	5
	DCM (HT, F)	15
**4. Technical setup**	Common setup	0
**5. Temperature/Time**	Room temperature 45 min	0
**6. Workup and purification**	Quench with water	0
	Extraction with CH_2Cl_2	3
	Dry over $MgSO_4$	0
	Filtration over SiO_2	0
**Total**		28,5
**Score**		**71,5**

Average score: 43, inadequate procedure

6. Summary of PMI and EcoScale

Table S6.1. EcoScale and PMI for each step of the present work.

Reaction step	Product	EcoScale	PMI
**Oxa-Michael**	**2a** (OH)	83.5	1.3
	**2b** (OBn)	69	1.2
	**2c** (OEt)	62	3.5
**Baeyer-Villiger oxidation**	**3a** (OH)	86.5	7.6
	**3b** (OBn)	83.5	5.1
	**3c** (OEt)	84.5	6.4
**Activation**	**4a** (OH)	79.5	6.1
	**4c** (OEt)	78	5.0
	**4d** (OAc)	64.5	6.9
	**4e** (OBn)	75	5.1
**Iodination**[a]		77	#
**Bernet-Vasella reaction**	**5a** (OH)	62.5	14.6
	**5d** (OAc)	79.5	10.2
	**5b** (OBn)	92.5	8.0
	**5c** (OEt)	74	11.1
**Benzylation**	**7a** (OH)	70	8.1
	**7b** (OBn)	70.5	6.3
	**7d** (OAc)	69	7.2
**Cross-metathesis homologation of carboxylic acid compounds**	**6a** (OH)	28	66.9[b]
	**6b** (OBn)	28.5	71.2[b]
	**6c** (OEt)	37	21.8[b]
	**6d** (OAc)	38	65.0[b] (31.4)[c]
**Cross-metathesis homologation of benzyl esters**	**8a** (OH)	34.5	25.2
	**8d** (OAc)	24	36.8
**Hydrogenation of carboxylic acid form compounds**	**9a** (OH)	88	11.5
	**9d** (OAc)	85.5	10.1
	**9b** (OBn)	65.5	10.9
	**9c** (OEt)	87	10.7
**Hydrogenation of benzyl esters**	**9a** (OH)	88	11.0
	**9d** (OAc)	85.5	10.6

**Average score for 9a (acidic route)**	72.1	18
**(benzyl route)**	64.9	9.4
**Average score for 9d (acidic route)**	73.5	16.9/11.3
**(benzyl route)**	71.2	10.5
**Average score for 9b**	70.1	16.9
**Average score for 9c**	71.4	9.8

Table S6.2. EcoScale and PMI for each step of Bauer's procedure

Step	EcoScale	PMI
**1. Condensation**	51	12.4
**2. Methylation**	72	
**3. Selective hydrogenation**	45	5.4
**4. Saponification**	60	31.1
**5. Silylation**	41	23.5
**6. Benzylation**	36.5	18.7
**7. Desilylation**	71	246.6
**Average for free carboxylic acid and free alcohol (1 to 4)**	57	12.2
**Average for free carboxylic acid and protected alcohol (1 to 5)**	61.1	14.5
**Average for protected carboxylic acid and free alcohol (1 to 7)**	53.8	48.2

Table S6.3. EcoScale and PMI for each step of Menhour's procedure

Step	EcoScale	PMI
**1. Condensation**	14	30.4
**2. Enantioselective acetylation**	5	20.7
**3. Cross-metathesis**	47.5	74.4
**4. Hydrogenation**	78	
**5. Deprotection of carboxylic acid**	71.5	327.4
**Average for free carboxylic acid and protected alcohol (1 to 5)**	43.2	90.6
**Average for protected carboxylic acid and alcohol (1 to 4)**	36.1	31.4

References

[1] F. Diot-Néant, L. M. M. Mouterde, J. Couvreur, F. Brunois, S. A. Miller, F. Allais, *Eur. Polym. J.* **2021**, *159*, 1–8.

[2] T. Kawai, M. Isobe, S. C. Peters, *Aust. J. Chem.* **1995**, *48*, 115–131.

[3] a) G. Bonneau, A. A. M. Peru, A. L. Flourat, F. Allais, *Green Chem.* **2018**, *20*, 2455–2458; b) F. Allais, G. Bonneau, A. A. M. Peru, A. L. Flourat, *Method for Converting Levoglucosenone into 4-Hydroxymethyl Butyrolactone or 4-Hydroxymethyl Butenolide without Using Any Organic Solvent and Catalyst*, **2018**, WO 2018/007764 A1. C) R. E. Deriaz, W. G. Overend, M. Stacey, E. G. Teece, L. F. Wiggins *J. Chem. Soc.* **1949**, 1879–1883. d) P. O. Miranda, F. Estevez, J. Quintana, C. I. Garcia, O. I. Brouard, J. I. Padron *J. Med. Chem*. **2004**, *47*, 292–295

[4] D. K. Mohapatra, K. Pulluri, S. Gajula, J. S. Yadav *Tetrahedron Lett.* **2015**, *56*, 6377–6380

[5] F. A. Jaipuri, M. F. Jofre, K. A. Schwarz, N. L. Pohl *Tetrahedron Lett.* **2004**, *45*, 4149–4152

[6] B. Menhour, F. O. Akong, P. Mayon, K. Plé, S. Bouquillon, S. Dorey, C. Clément, M. Deleu, D. Harakat, A. Haudrechy, *Tetrahedron* **2016**, *72*, 7488–7495

[7] K. Van Aken, L. Strekowski, L. Patiny, *Beilstein J. Org. Chem.* **2006**, *2*, 1–7.

[8] J. Bauer, K. Brandenburg, U. Zähringer, J. Rademann, *Chem. Eur. J.* **2006**, *12*, 7116–7124.
